# Socioeconomic status, individual behaviors and risk for mental disorders: A Mendelian randomization study

**DOI:** 10.1192/j.eurpsy.2022.18

**Published:** 2022-04-18

**Authors:** Jiahao Cai, Zixin Wei, Ming Chen, Lei He, Hongxuan Wang, Mei Li, Ying Peng

**Affiliations:** 1 Department of Neurology, Sun Yat-sen Memorial Hospital, Sun Yat-sen University, Guangzhou, China; 2 Department of Pulmonary and Critical Care Medicine, Sun Yat-sen Memorial Hospital, Sun Yat-sen University, Guangzhou, China; 3 Guangdong Provincial Key Laboratory of Malignant Tumor Epigenetics and Gene Regulation, Sun Yat-sen Memorial Hospital, Sun Yat-sen University, Guangzhou, China

**Keywords:** Bipolar disorder, individual behaviors, major depressive disorder, schizophrenia, socioeconomic status

## Abstract

**Background:**

There is increasing attention on the association of socioeconomic status and individual behaviors (SES/IB) with mental health. However, the impacts of SES/IB on mental disorders are still unclear. To provide evidence for establishing feasible strategies on disease screening and prevention, we implemented Mendelian randomization (MR) design to appraise causality between SES/IB and mental disorders.

**Methods:**

We conducted a two-sample MR study to assess the causal effects of SES and IB (dietary habits, habitual physical activity, smoking behaviors, drinking behaviors, sleeping behaviors, leisure sedentary behaviors, risky behaviors, and reproductive behaviors) on three mental disorders, including bipolar disorder, major depressive disorder and schizophrenia. A series of filtering steps were taken to select eligible genetic instruments robustly associated with each of the traits. Inverse variance weighted was used for primary analysis, with alternative MR methods including MR-Egger, weighted median, and weighted mode estimate. Complementary methods were further used to detect pleiotropic bias.

**Results:**

After Bonferroni correction and rigorous quality control, we identified that SES (educational attainment), smoking behaviors (smoking initiation, number of cigarettes per day), risky behaviors (adventurousness, number of sexual partners, automobile speeding propensity) and reproductive behavior (age at first birth) were causally associated with at least one of the mental disorders.

**Conclusions:**

MR study provides robust evidence that SES/IB play broad impacts on mental disorders.

## Introduction

Mental disorders have become the major contributors to overall morbidity and disability across the globe [1]. Approximately 30% of individuals suffer from mental disorders across the lifespan [2]. Standard treatment has no sufficient effect for a substantial proportion of patients with mental disorders [3]. Besides, it has been confirmed that suicide attempt is strongly associated with mental disorders [4]. As such, mental disorders have contributed to a majority of disease burdens, and the need to identify the potential risk factors is urgent for further prevention [5].

Human health has been linked to socioeconomic status (SES) and certain individual behaviors (IB) [6–8]. Determining the effect of SES/IB on human diseases might help us formulate targeted feasible strategies to mitigate disease risk. For example, smoking behavior has been an established high-risk factor for lung cancer, and thus effective tobacco control policies have been carried out by governments to greatly reduce the morbidity of lung cancer [9]. Observational epidemiological studies have attempted to investigate the relationship between SES/IB and mental disorders. Low income, and several behaviors/habits, like smoking, inadequate sleeping, and risky sexual behaviors, are more frequent in the population with mental diseases in existing large-scale meta-analyses and observational studies [10–12]. However, people are inclined to regard these behaviors/habits as the symptoms subsequent to disease development. Also, it is noteworthy that the inherent defects of conventional observational studies, like residual confounding and reverse causality, make it challenging to reveal replicable causes of mental disorders [13–15]. In this context, the imminent challenge is to decipher whether these mental disease-related SES/IB contribute to or are an effect of the diseases using a technique largely free from reverse causality and confounders.

The recently developed Mendelian randomization (MR) design has been implemented to investigate whether a specific exposure exerts a causal effect on a particular outcome [16]. As an analytic approach, MR design utilizes germline genetic variants [single nucleotide polymorphisms (SNPs)] as instrumental variants for exposures of interest [17]. Based on random assortment at meiosis and fixed allocation at conception for genetic variants, the MR study is less vulnerable to bias from confounders and reverse causality. Furthermore, a two-sample MR design using summary statistics from genome-wide association study (GWAS) greatly increases the statistical power of causality inference [18].

We have seen a surge of MR studies on the issue of mental disorders especially exploring the role of biomarkers. Still, the MR study focusing on the causal roles of SES/IB in the development of mental disorders is lacking. Despite a variety of SES/IB investigated in epidemiological research [11], the existing MR studies focused only a limited set of them on the prevention of mental disorders. Without broader investigation, additional underlying risk factors might be overlooked or unrevealed. Recent mental disorders-related MR studies were either limited by modest sample sizes or focusing on only a single trait [19,20]. However, SES/IB phenotypes are usually genetically correlated [21–23]. Causal inference of a single phenotype on the risk of mental disorders might be less reliable owing to shared genetic influence. Here we implemented a two-sample MR study to investigate the causal effect of a total of 28 SES/IB-related traits on three mental disorders, including bipolar disorder (BIP), major depressive disorder (MDD), and schizophrenia (SCZ). Considering potential shared genetic effect among the SES/IB traits, we further pruned all the genetic variants to obtain a smaller set of SNPs specific to each of the traits and then validated the robustness of the observed causality, which has been described by Grover et al. [24].

## Method and Materials

### Study design

**Figure 1.**
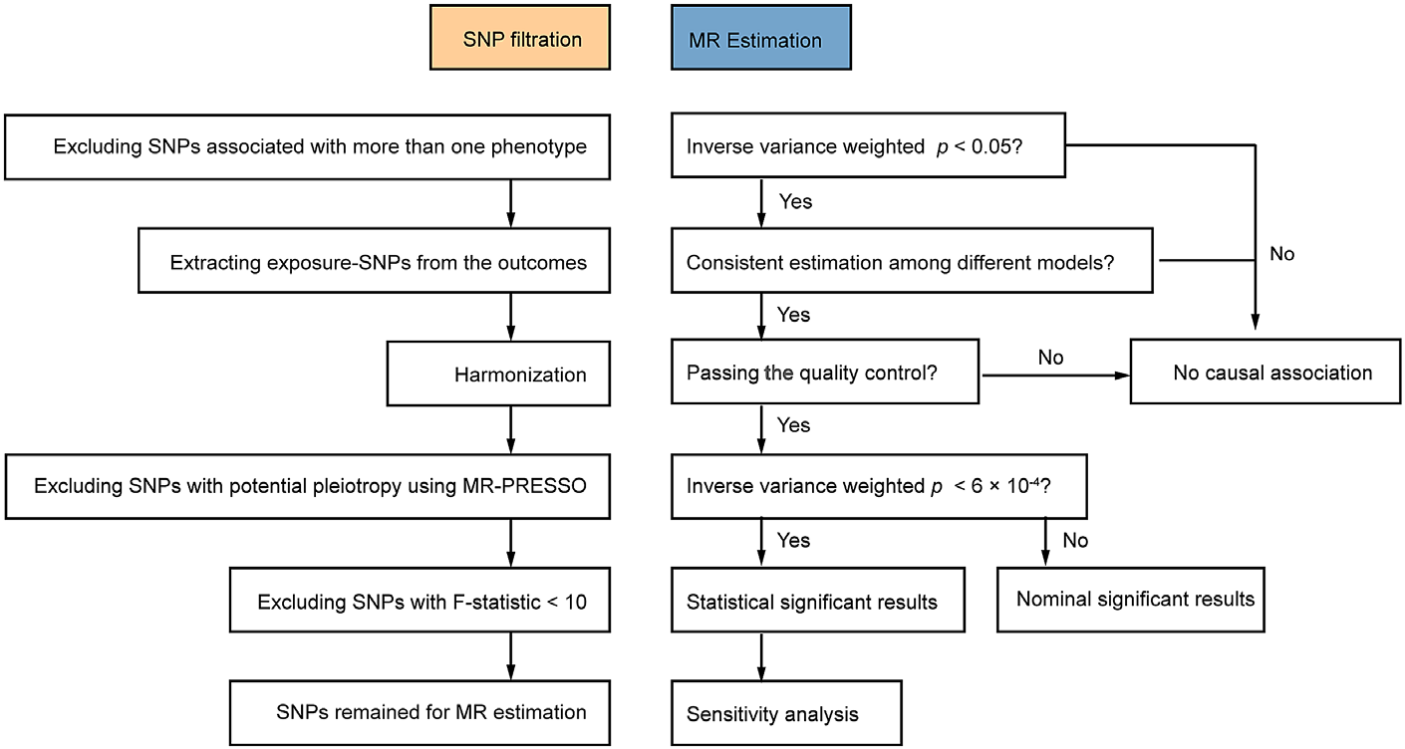
Flowchart of the current MR design. SNP, single nucleotide polymorphism; MR-PRESSO, Mendelian randomization-pleiotropy residual sum and outlier.

Based on previous related investigations on mental disorders (Supplementary Table S1), we curated data on 28 SES/IB-related factors with GWAS summary data available (Supplementary Table S2). We then performed a two-sample MR study to explore their causality with three mental disorders including BIP, MDD, and SCZ. The flowchart of the current MR design was presented in Figure 1. Statistical analysis was performed by the two-sample MR package (version 0.5.4) and MR-PRESSO package (version 1.0) of the R program (version 4.0.0). Only summary-level statistics were used in our study and thus ethical approval was not required.

### GWAS data for SES/IB-related factors

In the present study, the 28 phenotypes were grouped into SES and eight kinds of IB including dietary behaviors (dietary composition), habitual physical activity, smoking behaviors, drinking behaviors, sleeping behaviors, leisure sedentary behaviors, risky behaviors, and reproductive behaviors. Genetic instruments for these phenotypes were obtained from publicly available summary-level data of 11 published GWAS restricted to European descent [21–23,25–32]. See Supplementary Table S2 for detailed definitions and descriptions for each of the phenotypes.

### GWAS data for mental disorders

Summary statistics for mental disorders including BIP, MDD, and SCZ were obtained from the Psychiatric Genomics Consortium (PGC; http://www.med.unc.edu/pgc/). As a two-sample MR design using summary-level statistics assumes independent samples between exposure and outcome, two large cohorts (UK biobank and 23andMe) were removed from the psychiatric phenotypes as they shared significant sample overlapping with SES/IB-related phenotypes. Hence, the final datasets included 53,510 individuals for BIP (20,352 cases and 31,358 controls; European ancestry), 142,646 individuals for MDD (45,396 cases and 97,250 controls; European ancestry) and 79,845 individuals for SCZ (34,241 cases and 45,604 controls; 93.5% of European ancestry) [33–35]. See Supplementary Table S3 for detailed descriptions of the GWAS meta-analysis on three mental disorders.

### Selection of genetic instruments for SES/IB-related phenotypes

We obtained independent genetic variants robustly associated with SES/IB (*p* < 5 × 10^−8^) by clumping SNPs at the threshold of linkage disequilibrium (LD) *r* ^2^ > 0.001 within a 10,000-kilobase window in the 1,000 Genomes European reference panel (detailed in Supplementary Datasets). We then performed SNPs filtering according to a series of procedures: (a) To avoid the pleiotropic effect, SNPs associated with more than one phenotype were discarded. For example, “rs1008078” is significantly associated with both educational attainment and smoking initiation, for which case this SNP was discarded. (b) We then extracted the SNPs from the GWAS data of outcomes (mental disorders). In this step, we removed the SNPs associated with outcomes at genome-wide significance, and for those absent in the outcomes, we identified proxy SNPs at the threshold of LD *r* ^2^ > 0.80 based on the 1,000 Genomes phase 3 data from Single Nucleotide Polymorphisms Annotator (SNiPA) database (https://snipa.helmholtz-muenchen.de/snipa3/). SNPs absent in the outcomes without appropriate proxy SNPs available were then discarded. (c) We harmonized the exposure-outcome datasets to exclude strand-ambiguous SNPs with intermediated allele frequency (AF > 0.42). (d) MR-pleiotropy residual sum and outlier (MR-PRESSO) was then implemented to identify and remove SNPs with potential pleiotropy at the threshold of *p* < 0.05 (detailed in Supplementary Tables S4 and S5). *F*-statistics were finally calculated to quantify the strength of the selected SNPs.

### MR estimates

The random-effects inverse-variance weighted (IVW) method was used for primary MR analysis. This method combines the Wald ratio causal estimates obtained from each of the SNPs and yields a pooled causal effect of the exposure on the outcome using a meta-analysis approach. Compared to standard fixed-effects IVW, the random-effects IVW method obtains a more conservative causal inference accounting for the uncertainty due to pleiotropy [36]. Unless otherwise specified, all "IVW" here refers to random-effects IVW. We considered a statistically significant result using a Bonferroni-corrected threshold of *p* < 6 × 10^−4^ (0.05/28 exposures/three outcomes). Nominal significant results were identified at *p* < 0.05 threshold.

We then compared the pattern of IVW results with other MR methods including weighted median, MR-Egger, and weighted mode estimation except for the phenotype “number of children ever born” due to only two SNPs available, making only the IVW method applicable [37]. A consistent direction of estimates across all the MR methods increases confidence in causal evidence. Detailed assumptions for these MR methods were presented in Supplementary Table S6.

### Quality control of MR estimation

For significant results (*p* < 0.05), we performed quality control using various methods. We first evaluated heterogeneity for the IVW model using Cochran’s *Q* test, quantified with *I* ^2^ statistics [38]. Cochran’s *Q* test *p* < 0.05 and *I* ^2^ > 25% indicated potential heterogeneity. However, the presence of heterogeneity does not necessarily invalidate the random-effects IVW estimate when the overall heterogeneity is balanced. Even though pooled IVW estimate fails to balance heterogeneity, the intercept term of MR-Egger regression could be used to account for this bias [36]. Specifically, MR-Egger regression allows a non-zero intercept, which can be used to detect directional pleiotropy [39]. We also evaluated the heterogeneity of MR-Egger regression using Rucker’s *Q′* test and obtained a difference *Q–Q′* to determine whether IVW was appropriate to interpret a specific causal inference [40]. Finally, we performed a leave-one-out analysis to assess whether there was an individual SNP strongly driving a specific causal estimate.

Based on the MR models and pleiotropy assessments mentioned above, we considered a relatively robust causal inference meeting the following items: (a) MR analyses presented a consistent direction of causal estimates among different methods. (b) Intercept term derived from MR-Egger regression suggested no directional pleiotropy detected (*p* > 0.05). (c) Slight *Q–Q′* difference suggested IVW as an appropriate model for interpretation of the causal inference. (d) Leave-one-out analysis suggested causal estimate was not strongly driven by a single SNP. Considering potential reverse causation that might bias MR estimates, we further conducted a directionality test using MR Steiger to evaluate whether the assumption that exposure causes outcome is valid [41,42].

### Sensitivity analysis

Given that SES/IB-related phenotypes were genetically correlated [21–23,32], MR estimates might be biased owing to shared genetic influence. Hence, we conducted a sensitivity analysis using phenotypes relatively independent to further validate the robustness of the significant results, which have been described by Grover et al [24]. Specifically, we considered the existence of genetic overlapping across different traits at a threshold of *r* ^2^ > 0.80. Such an approach allowed us to include only genetic loci specific to each phenotype and repeat IVW analysis. Furthermore, we set a more conservative threshold of *r* ^2^ > 0.01 across the phenotypes at the cost of reduced power.

We also looked up three databases (Ensembl, GWAS Catalog, and PhenoScanner) for any potential associations of the selected SNPs with other risk factors (confounders) of mental disorders identified by previous MR studies. A specifically confirmed confounder investigated among different MR studies should present consistent results. Once potential confounders were detected, we combined and removed the corresponding SNPs manually and repeated MR analysis.

### Power calculation

We calculated the statistic power using the method described by Brion et al [43]. (https://shiny.cnsgenomics.com/mRnd/). A sufficient power of over 80% was recommended.

## Results

### Study overview

**Figure 2.**
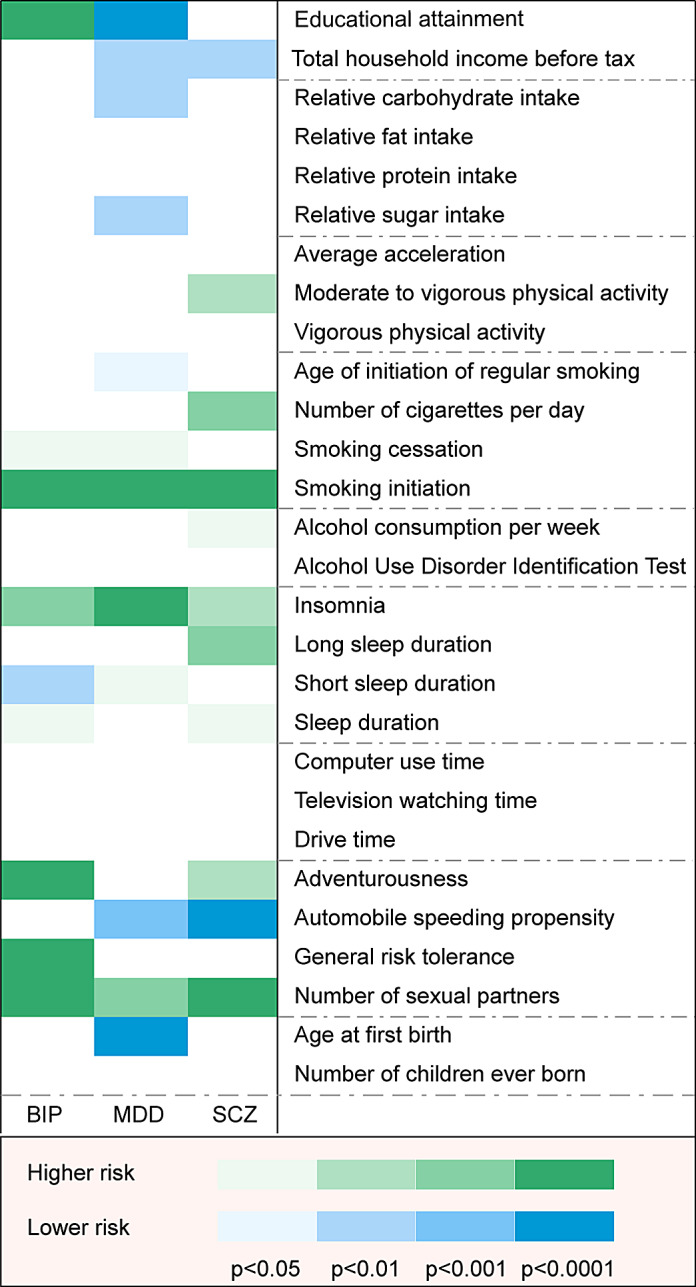
Primary analysis of associations between socioeconomic status/individual behaviors and three mental disorders. BIP, bipolar disorder; MDD, major depressive disorder; SCZ, schizophrenia.

The current study appraised the causal effect of 28 genetically predicted SES/IB-related phenotypes on three mental disorders such as BIP, MDD, and SCZ. After rigorous SNPs filtering procedures, the number of SNPs finally used for each of the phenotypes varied from two to 369 (Supplementary Table S7). The *F*-statistics ranged from 15.74 to 1520.00, suggesting bias owing to the employment of weak instruments unlikely (Supplementary Table S8). In the primary analysis, a total of 32 causative relationship features were identified at *p* < 0.05 (Figure 2). Complete effect estimates derived from different MR models were presented in Supplementary Table S7. Statistical power was presented in Supplementary Table S8.

### Causal effects of SES/IB-related phenotypes on mental disorders

**Figure 3.**
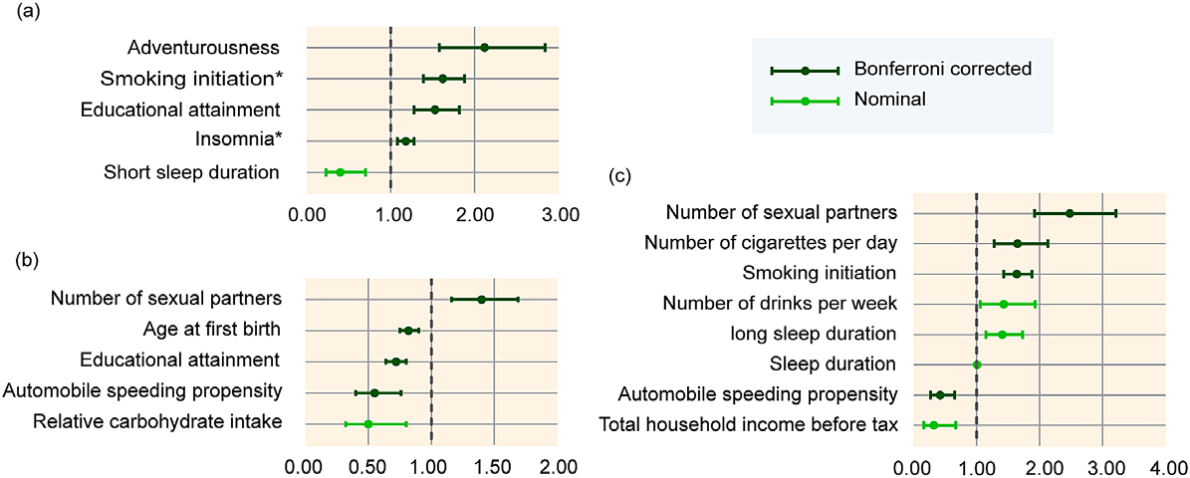
Mendelian randomization estimates that passed quality control. Panels (a)–(c) respectively present inverse variance weighted estimates of risk factors on bipolar disorder, major depressive disorder, and schizophrenia. *False direction from exposure to outcome, which was identified by Mendelian randomization Steiger test.

After Bonferroni correction, we observed several SES/IB-related factors with causal effects on mental disorders in IVW model (Figure 3; Supplementary Figure S1, Supplementary Table S7). We found that educational attainment (odds ratio [OR]: 1.53; 95% confidence interval [CI]: 1.28–1.82; *p* = 2.91 × 10^−6^), smoking initiation (OR: 1.62; 95% CI: 1.39–1.88; *p* = 2.78 × 10^−10^), insomnia (OR: 1.18; 95% CI: 1.08–1.28; *p* = 1.30 × 10^−4^) and adventurousness (OR: 2.12; 95% CI: 1.58–2.84; *p* = 4.22 × 10^−7^) were associated with increased BIP risk. For MDD, educational attainment (OR: 0.72; 95% CI: 0.64–0.80; *p* = 1.43 × 10^−9^), automobile speeding propensity (OR: 0.55; 95% CI: 0.40–0.76; *p* = 2.26 × 10^−4^), and age at first birth (OR: 0.82; 95% CI: 0.75–0.90; *p* = 2.17 × 10^−5^) were identified as protective factors, while the number of sexual partners (OR: 1.40; 95% CI: 1.16–1.69; *p* = 5.04 × 10^−4^) acted as a risk factor. Primary analysis also indicated smoking initiation (OR: 1.64; 95% CI: 1.43–1.88; *p* = 2.08 × 10^−12^), number of cigarettes per day (OR: 1.65; 95% CI: 1.28–2.13; *p* = 1.26 × 10^−4^), and number of sexual partners (OR: 2.48; 95% CI: 1.92–3.21; *p* = 3.17 × 10^−12^) exerted a deleterious effect on SCZ, while automobile speeding propensity (OR: 0.55; 95% CI: 0.40–0.76; *p* = 2.26 × 10^−4^) exerted a protective effect on SCZ. While moderate heterogeneity was detected for some results (*I* ^2^ index 38–57%), causal estimates were consistent among the MR models and MR-Egger intercept indicated no directional pleiotropy detected (Supplementary Tables S7 and S9). Leave-one-out analysis suggested the detected estimate effects were not reliant on specific SNP (Supplementary Figure S2). Furthermore, Rucker framework indicated IVW model with better goodness-of-fit based on slight difference of *Q–Q′* (Supplementary Table S9). Using MR steiger test, we identified that the MR estimates of insomnia and smoking initiating on BIP might be biased by reverse causation (Figure 3).

We also observed that general risk tolerance and the number of sexual partners were associated with increased risk of BIP in the IVW model (*p* < 6 × 10^−4^) (Supplementary Table S7). However, the inconsistent directions of estimates across various MR methods suggested the detected causality might be biased owing to potential pleiotropy (Supplementary Tables S7 and S9). Similarly, while primary analysis also detected causal effects of smoking initiation and insomnia on MDD (*p* < 6 × 10^−4^), quality control suggested association derived from the IVW model was more likely due to pleiotropic bias (Supplementary Tables S7 and S9).

Other nominal results at the *p* < 0.05 threshold were observed but only a subset of them remained robust in pleiotropy assessment, including total household income before tax, relative carbohydrate intake, long sleep duration, short sleep duration, sleep duration, and alcohol consumption (number of drinks) per week (Figures 2 and 3; Supplementary Figures S1 and S2, Supplementary Tables S7 and S9).

### Genetic overlap among SES/IB-related phenotypes

Given two different LD thresholds (*r* ^2^ ≤ 0.80 and *r* ^2^ ≤ 0.01) across all the SES/IB-related phenotypes, we obtained mutually independent phenotypes proxied by a smaller set of SNPs. Even though at a more conservative cut-off of LD *r* ^2^ ≤ 0.01, the causal relationships were still robust except for the association of automobile speeding propensity with MDD (Table 1).

**Table 1.** Effect estimates using independent loci among phenotypic traits for observed statistically significant results (*p* < 6 × 10^−4^).

			*r* ^2^ ≤ 0.80 across phenotypes			*r* ^2^ ≤ 0.01 across phenotypes		
Exposures	Outcomes	Estimates method	SNPs, N	OR (95% CI)	*p*	SNPs, N	OR (95% CI)	*p*
Education attainment	BIP	IVW	360	1.53 (1.28, 1.83)	2.77E-06	338	1.50 (1.25, 1.80)	1.20E-05
Smoking initiation	BIP	IVW	166	1.56 (1.34, 1.82)	1.99E-08	80	1.57 (1.25, 1.97)	9.23E-05
Insomnia	BIP	IVW	141	1.19 (1.09, 1.29)	8.21E-05	104	1.15 (1.05, 1.27)	3.01E-03
Adventurousness	BIP	IVW	91	2.02 (1.50, 2.72)	4.20E-06	56	1.94 (1.39, 2.71)	1.07E-04
Education attainment	MDD	IVW	365	0.72 (0.64, 0.80)	2.06E-09	343	0.71 (0.63, 0.79)	1.52E-09
Automobile speeding propensity	MDD	IVW	25	0.51 (0.36, 0.73)	1.96E-04	8	0.84 (0.54, 1.33)	0.46
Number of sexual partners	MDD	IVW	66	1.41 (1.16, 1.72)	6.46E-04	40	1.30 (1.02, 1.66)	0.03
Age at first birth	MDD	IVW	8	0.83 (0.76, 0.92)	2.56E-04	3	0.73 (0.65, 0.83)	8.39E-07
Number of cigarettes per day	SCZ	IVW	26	1.68 (1.30, 2.17)	7.56E-05	16	1.60 (1.19, 2.14)	1.74E-03
Smoking initiation	SCZ	IVW	159	1.59 (1.37, 1.84)	4.38E-10	81	1.81 (1.45, 2.26)	1.68E-07
Automobile speeding propensity	SCZ	IVW	24	0.40 (0.25, 0.65)	1.85E-04	11	0.36 (0.17, 0.73)	4.80E-03
Number of sexual partners	SCZ	IVW	59	2.42 (1.86, 3.14)	4.13E-11	36	2.67 (1.93, 3.69)	3.40E-09

Abbreviations: BIP, bipolar disorder; CI, confidence interval; IVW, inverse variance weighted; MDD, major depressive disorder; OR, odds ratio; SCZ, schizophrenia; SNP, single nucleotide polymorphism.

### Genetic variants associated with potential confounders

We identified potential confounders for mental disorders from previous MR studies (Supplementary Table S10). We then identified SNPs in the present study that were associated with potential confounders by comprehensively screening three databases, including Ensembl, PhenoScanner, and GWAS Catalog (Supplementary Table S11). The identified genetic variants were combined and then eliminated to check for the overall influence of confounders on the effect estimates. Our sensitivity analysis suggested no overall bias of detected confounders on the observed robust associations (Supplementary Table S12).

## Discussion

To date, the prevention of mental disorders remains a challenge. Despite underlying biomolecular mechanisms have been widely investigated [44], there is a paucity of evidence about the causal role of SES/IB-related phenotypes in mental disorders. To provide feasible strategies for mental disorders prevention, we used a two-sample MR design to research the causal relationships of 28 SES/IB-related factors with BIP, MDD, and SCZ. Our results indicated that socioeconomic status, smoking behaviors, sleeping behaviors, risky behaviors, and reproductive behaviors play critical roles in mental disorder development.

### Socioeconomic status and mental disorders

We observed that socioeconomic status played broad effect on mental disorders. Previous observational studies yielded inconsistent results on the association between educational attainment and mental disorders [13,14,45,46]. However, it should be noted that associations observed in such conventional epidemiological studies were limited by modest sample sizes, reverse causality, and the inability to fully mitigate confounding effects. Intriguingly, using a large-scale MR design, the present work showed that higher educational attainment was causally associated with a higher BIP risk and a lower MDD risk. The opposite effect of educational attainment on these two mental disorders might be confusing. One of the possible explanations for the detrimental effect of higher education achievement on BIP is that this population faces more social competition and challenges. In contrast, the protective role of educational attainment in MDD reflects its benefits from subsequent healthier living habits, like lower BMI, which has been reported to be associated with a lower risk of MDD [47,48]. Indeed, using 74 genetic variants proxying educational attainment obtained from 293,723 individuals, Wray et al have confirmed a causal association between higher educational attainment and a lower MDD risk [35]. In our work, this association was further validated using 369 SNPs obtained from approximately 1.1 million individuals. Of note, our finding revealed the total effect of educational attainment on mental disorders, and further work is warranted to determine the underlying pathways mediating the association between educational attainment and mental disorders.

We also find some evidence of the association between higher total household income before tax and a lower risk of SCZ. Income inequality in SCZ incidence has been noted before [49–51]. Schizophrenia patients are forced to unemployment due to severe symptoms including disability, and on the other hand, unemployment and low income with attendant psychosocial stress might, in turn, contribute to the development of SCZ [49]. Corroboration of causality in our MR analysis further supported an emotional benefit from a higher income level.

### Smoking behaviors and mental disorders

Smoking behaviors were strongly associated with mental disorders, especially with SCZ. Observational studies have found that cigarette smoking was associated with an increased risk of mental disorders [52–54]. Our work suggested individual ever being a regular smoker (smoking initiation) was associated with an increased risk of SCZ. Besides, we validated smoking heaviness (number of cigarettes per day) as a risk factor for SCZ [55]. For the mechanisms underlying a causal role of nicotine on mental disorders incidence, especially SCZ, previous studies have emphasized the effects of nicotine on the dopamine system including increasing dopamine release and D2 receptors super-sensitivity [56,57]. While the present study also observed a causal effect of smoking initiation on higher MDD risk using the IVW method, complementary MR methods suggested the association detected from IVW was more likely due to bias instead of any potential causal mechanisms. We noticed that Howard et al reported a reverse association of MDD with smoking behavior (ever vs., never smoked), thus there existed a possibility that the observed causality of smoking initiation on MDD in the current study might be biased owing to a feedback loop [41,58]. However, a recent MR study reported a causal effect of smoking initiation on MDD using 347 SNPs at the threshold of LD *r* ^2^ ≤ 0.1 [20]. Here we restricted the number of SNPs to 183 at a lower threshold of LD *r* ^2^ ≤ 0.001, increasing confidence to employ independent SNPs for MR analysis. Besides, Yuan et al used summary statistics of MDD from UK biobank individuals, which led to sample-overlapping with smoking initiation [20,21]. More studies are needed to verify the association of cigarette smoking with MDD in a causal fashion.

### Sleeping behaviors and mental disorders

Sleeping behaviors were associated with mental disorders. Our results supported that those with symptoms of insomnia were more likely to suffer from BIP. Previous observational studies have identified insomnia as a risk factor for BIP [59,60]. A recent MR study also confirmed a causal effect of insomnia on BIP using 22 SNPs extracted from GWAS summary data on 386,533 individuals [19], and the present work employed 146 independent SNPs from a GWAS on 1,331,010 individuals of European descent and validated the robustness of causality using unique loci specific to insomnia in avoidance of shared genetic effects with other SES/IB-related phenotypes [29]. However, when testing directionality using MR Steiger, we found that the MR estimation might be biased by reverse causation.

We also observed a suggestive inverse association of short sleep duration (sleeping for 6 h or less) with BIP. This was consistent with a meta-analysis presenting a shorter sleep duration in controls compared to cases with BIP [61]. We also found some evidence for the association of long sleep duration (binary phenotype defined as sleeping for 9 h or more) and sleeping duration (continuous phenotype) with increased risk of SCZ. This was consistent with the previous observational studies suggesting an increased sleep duration in SCZ patients [62,63].

These findings implicated critical and sophisticated roles of sleep patterns in mental disorder development. In terms of mechanisms, although Altena et al have suggested poor sleeping quality might lead to dysregulation of emotional processing [64], pathways from sleep patterns to mental disorders remained elucidated and more work is warranted to further decipher the potential mechanisms.

### Risky behaviors and mental disorders

We also found evidence of wide and strong associations between risk-taking tendency and mental disorders. Compared with individuals with a cautious personality, those with an adventurous personality (adventurousness) were more likely to develop BIP. Among the risky behaviors, an increase in the number of sexual partners was strongly associated with susceptibility to MDD and SCZ. Promiscuity has been noted in patients with mental disorders [65–67]. However, these findings were unable to distinguish between sexual promiscuity causing mental disorders and mental disorders causing sexual promiscuity. In the past, risky sexual behaviors have been mainly considered as a sort of self-medication with sex for patients with mental disorders, especially for MDD patients, who are prone to sexual promiscuity for releasing tension and seeking emotional support when feeling hopeless and worthless [68,69]. Whereas on the other hand, harmful consequences of promiscuity like unintended pregnancy and sexually transmitted diseases may be involved in the episode of mental disorders. The present study highlighted the role of promiscuity in the development of mental disorders, especially for MDD and SCZ.

We also observed an unexpected inverse association of automobile speeding propensity with SCZ. Interpretation should be cautious for such an unexpected beneficial effect. Lower driving speed in SCZ patients has been reported by Fuermaier et al and it tended to be explained that SCZ patients might drive slower to compensate for slower cognitive processing after SCZ [70]. To some extent, driving speed reflects intelligence and cognitive status [71,72]. Previous MR study has reported that higher intelligence was causally associated with lower SCZ risk [73]. The observed association between automobile speeding propensity and SCZ risk might be mediated with intelligence. Latent mechanisms should be further investigated. Besides, although our work suggested a causal effect of automobile speeding propensity on MDD, the sensitivity analysis using unique loci specific to automobile speeding propensity obtained a null estimate, making a conclusive explanation unavailable. To the best of our knowledge, there is a lack of epidemiologic population-based studies exploring the role of risky behaviors on the vulnerability to mental disorders. Therefore, the interpretations of our MR results are less evidence-based and more speculative. More independent lines of evidence are required to validate these results.

### Reproductive behaviors and mental disorders

As for reproductive behaviors, we found a strong causal association between age at first birth and MDD, suggesting a potential emotional benefit by appropriately delaying first birth. This was consistent with previous observational researches suggesting a generally negative association between age at first birth and depression symptoms [74,75]. While it has been proposed that a poor start to family formation, lower socioeconomic attainment and poorer health status subsequent to the early age of first birth might cause depression symptoms, more comprehensive mechanisms underlying the emotional benefit from delaying firth birth should be further explored [74].

### Significance of the current study

Although the current work identified certain causal roles of SES/IB-related phenotypes in the issue of mental disorders, interpretation of the MR results should be cautious. Methodological researchers have cautioned against interpreting the MR causal estimates as the expected impact of interventions for the risk factor in a clinical setting [76]. Indeed, the MR causal estimate reflects the impact of life-long exposure to the risk factors, and it might not be clinically meaningful by intervening in the risk factor within a specific period [41]. Besides, time-dependent impacts should also be taken into account. For example, an MR study has supported the protective role of vitamin D in lowering the risk of multiple sclerosis [77], but this protective effect is limited to childhood and puberty periods, and ignoring this context might mislead clinical interventions [78].

Then how can MR results be more appropriately interpreted? There are a few points worth noting. First, keep in mind that univariable MR analysis only reveals a total effect of exposure on the outcome, instead of a direct effect. The pathways from exposure to an outcome might be complex, and multivariable MR analysis is a strong complement to univariable MR study exploring the potential mediators. Specifically in this study, substance use and risky sexual behaviors are often comorbid, suggesting there might be potential interaction existing between them [79]. As such, there might be the possibility that the effect of substance use on mental disorders is mediated with risky sexual behaviors, or the effect of risky sexual behaviors on mental disorders is mediated with substance use. Second, while extrapolating the MR results to guide clinical intervention might be inappropriate, causal inference using MR design might contribute to screening specific populations who are susceptible to developing diseases. For example, taken together with multivariable MR analysis, our previous work suggested a higher cardiovascular risk in those prescribed with opioids, and enhancing obesity management and disease surveillance is of great importance in this population [80]. Third, for certain established causal associations, they would provide strong support for conducting randomized controlled trials to further determine the impact of an intervention on disease development in the real world, subject to ethical principles.

### Strengths and limitations

Our work has several important strengths. We appraised causality between a broad panel of SES/IB-related factors and mental disorders using an MR framework. By utilizing genetic variants as instrumental variants, MR design can largely reduce residual confounding and mitigate reverse causality that is commonly presented in conventional observational studies [15]. Besides, the employment of complementary methods for pleiotropy and sensitivity analysis allowed us to rigorously evaluate the violation of MR assumptions. By incorporating various SES/IB-related traits into MR analysis, we were able to include genetic variants associated exclusively with a single phenotype to limit bias owing to potential shared genetic effects among the phenotypes. In addition, using datasets restricted to mainly European ancestry limited the bias due to population stratification.

Several limitations should be considered in our study. First, the present study relied largely on self-report phenotypes, which might be subject to report bias. Second, although *F*-statistics of genetic variants indicated no weak instruments were employed, statistic power for certain results was modest. Such modest power might result from insufficient sample size or genetic variants, and hence "false negative" could not be fully ruled out. As such, larger GWAS data were expected for researchers to further verify these results. Third, utilization of summary-level data could not allow us to further perform gender or age-stratified analyses. Fourth, for the lack of full GWAS data for some of the exposures like adventurousness, we did not perform MR study in the other direction. A bidirectional study is warranted when the full GWAS statistics were available in the future. Fifth, the shared genetic influence between SES/IB and mental disorders could not be fully ruled out, which might introduce potential bias to the MR estimates. However, based on an array of statistical methods for quality control, the pleiotropic bias is less likely in our study.

### Conclusion

Our work found that SES/IB-related phenotypes including socioeconomic status, smoking behaviors, sleeping behaviors, risky behaviors, and reproductive behaviors exerted broad impacts on mental disorders (BIP, MDD, SCZ) to different degrees. These findings might have implications for establishing feasible strategies for disease screening and prevention.

## Data Availability

All data used in our study are publicly available. For SES/IB-related phenotypes in our study, corresponding summary statistics were available from each of the original GWAS researches. Summary statistics for three mental disorders (BIP, MDD, and SCZ) were available on the PGC website: http://www.med.unc.edu/pgc/.

## Supplementary material

For supplementary material accompanying this paper visit http://doi.org/10.1192/j.eurpsy.2022.18. click here to view supplementary material
