# Supplementary material for: Socioeconomic status, individual behaviors and risk for mental disorders: A Mendelian randomization study
Source: Eur Psychiatry. 2022 Apr 18;65(1):e28. doi: 10.1192/j.eurpsy.2022.18 (PMC9158396; doi:10.1192/j.eurpsy.2022.18)
Supplement: Supplementary file 1 [file epasup.zip › S0924933822000189sup001.pdf]

# Supplementary material

**Socioeconomic Status, Individual Behaviors and Risk of Mental disorders: A Mendelian Randomization Study**

Corresponding to: Ying Peng. Department of Neurology, Sun Yat-sen Memorial Hospital, Sun Yat-sen University, Guangzhou 510120, China. Email: pengy2@mail.sysu.edu.cn. Telephone: 86-13380051581.

Supplementary Introduction for the Mendelian Randomization Approach ........................................................1

**Table S1.** Previous studies investigating associations between socioeconomic status/individual behaviors and mental disorders.......3

**Table S2.** Detailed definitions and descriptions of exposure phenotypes in the present study........................................5

**Table S3.** Detailed descriptions for GWAS meta-analysis of 3 mental disorders ........................................11

**Table S4.** Details of SNPs filtering procedures........................................12

**Table S5.** List of genetic variants associated with more than one phenotype ........................................14

**Table S6.** Descriptions of the Mendelian randomization (MR) methods........................................15

**Table S7.** Mendelian randomization estimates for the association of socioeconomic status/individual behaviors with 3 mental disorders derived from different models........................................17

**Table S8.** Statistical power calculation for Mendelian randomization effects derived from random-effects inverse variance weighted method........................................22

**Table S9.** Heterogeneity and pleiotropy assessment for significant results ($p < 0.05$)........................................24

**Table S10.** Confirmed confounders for mental disorders (BIP, MDD and SCZ) from previous Mendelian randomization studies......26

**Table S11.** SNPs associated with confirmed confounders at genome-wide significance ($p < 5 \times 10^{-8}$)........................................28

**Table S12.** Inverse variance weighted (IVW) analysis after exclusion of SNPs associated with potential confounders......................36

**Figure S1.** Scatter plots showing robust genetic associations of socioeconomic status/individual behaviors with mental disorders ...37

**Figure S2.** Leave-one-out analysis for all significant causal relationships detected in primary analysis ........................................56

## Supplementary Introduction for the Mendelian Randomization Approach

In recent years, the Mendelian randomization (MR) approach is widely applied to explore the risk factors of diseases (Smith and Ebrahim, 2003). The concept of MR was firstly attributed to Katan in 1986 and subsequently developed by the others, with instrumental variables (IVs) in econometrics introduced (Katan, 1986). In the MR framework, genetic variants are used as IVs to represent the exposure phenotypes of interest. When a certain phenotype is determined by the genotype, and the phenotype leads to the onset of a certain disease, then it can be considered that the genotype affects the disease through the phenotype (Thomas and Conti, 2004). Based on the random assortment at meiosis and fixed allocation at conception for genetic variants, MR design is less vulnerable to bias from confounders and reverse causality. Thus, genetic variations (single nucleotide polymorphisms, SNPs) can be used as IVs to infer causal relationships between phenotypes and the risk of diseases (Burgess et al., 2013). IVs must meet the following three assumptions in the MR framework: i) IVs are robustly associated with the exposure phenotype; ii) IVs are not associated with any potential confounders; iii) IVs influence the outcome only through the exposure phenotype (Emdin et al., 2017). The first assumption is known as the "relevance" assumption. The second (the "exclusiveness" assumption) and third (the "independence" assumption) assumptions are collectively known as independence from horizontal pleiotropy. Horizontal pleiotropy is also called "biological pleiotropy", which refers to an SNP associated with multiple exposure phenotypes and affecting the outcome independent of the risk factor but through other pathways. Violation of the fundamental assumptions can introduce bias in the MR analysis. To meet the first assumption, SNPs should be strongly associated with the exposure phenotype at genome-wide significance ($p < 5 \times 10^{-8}$) with sufficient statistical strength ($F$-statistics > 10) (Burgess and Thompson, 2011). Whereas for the second and third assumptions, an array of sensitivity analysis methods have been established to evaluate any potential pleiotropy. For example, the intercept derived from MR-Egger regression can be used to detect horizontal pleiotropy. More detailed, the intercept with a p > 0.05 suggests the rejection of the "non-zero intercept" hypothesis, indicating no pleiotropy detected (Burgess and Thompson, 2017).

The present work performed a two-sample MR (TSMR) analysis based on summary-level statistics from genome-wide association studies (GWAS). The minimum information required for TSMR analysis includes SNP identification (ID), effect-allele (EA), effect size (BETA), and standard error (SE) of BETA. Other non-essential but useful information for MR analysis includes other-allele (OA), effect allele frequency (EAF), p-value, sample size (N), and so on. In our study, four models were used to estimate the causality including inverse weighted variance (IVW), MR-Egger regression, weighted median and weighted mode estimation. Detailed instructions for each of the models were presented in Table S6. Rigorous SNPs quality control and complementary statistical methods were conducted to detect and correct any potential pleiotropy.

**Reference**

Burgess, S., Butterworth, A., Thompson, S.G., 2013. Mendelian randomization analysis with multiple genetic variants using summarized data. Genet. Epidemiol. 37, 658-665.

Burgess, S., Thompson, S.G., 2011. Avoiding bias from weak instruments in Mendelian randomization studies. Int. J. Epidemiol. 40, 755-764.

Burgess, S., Thompson, S.G., 2017. Interpreting findings from Mendelian randomization using the MR-Egger method. Eur. J. Epidemiol. 32, 377-389.

Emdin, C.A., Khera, A.V., Kathiresan, S., 2017. Mendelian Randomization. JAMA 318, 1925-1926.

Katan, M.B., 1986. Apolipoprotein E isoforms, serum cholesterol, and cancer. Lancet i, 507-508.

Smith, G.D., Ebrahim, S., 2003. 'Mendelian randomization': can genetic epidemiology contribute to understanding environmental determinants of disease? Int. J. Epidemiol. 32, 1-22.

Thomas, D.C., Conti, D.V., 2004. Commentary: the concept of 'Mendelian Randomization'. Int. J. Epidemiol. 33, 21-25.

Table S1. Previous studies investigating associations between socioeconomic status/individual behaviors and mental disorders.

| Exposures | Mental disorders | Type of study | PMID | Primary Results |
|---|---|---|---|---|
| Education level | Major depressive disorder | Cohort study | 25605025 | Low educational level was associated with increased risk of major depressive disorder. |
| Educational attainment | Bipolar disorder | Case-control | 16524631 | Increased educational attainment was associated with increased risk of bipolar disorder. |
| Family income | Major depressive disorder | Cohort study | 29450462 | Low family income level was associated with increased risk of major depressive disorder. |
| Sugar-sweetened beverage consumption | Clinical depression or depressive symptoms | Meta-analysis | 30419536 | Regular consumption of sugar-sweetened beverage was associated with increased risk of depression. |
| Physical activity levels | Clinical depression or depressive symptoms | Meta-analysis | 29690792 | Higher physical activity level was associated with reduced risk of depression. |
| Physical activity level | Anxiety symptoms | Meta-analysis | 31542132 | Higher physical activity level was associated with reduced risk of anxiety symptoms. |
| Tobacco smoking behaviors | Bipolar disorder | Case-control | 19267698 | Bipolar disorder was associated with higher prevalence of tobacco smoking behaviors. |
| Smoking status (current, former and never) | Depression | Meta-analysis | 24935795 | Smoking was associated with increased risk of depression relative to both never smokers and former smokers |
| Smoking status (smoker or nonsmoker) | Schizophrenia | Meta-analysis | 30102383 | Smokers had an increased risk of schizophrenia compared with nonsmokers |
| Alcohol consumption (abstinence, light, moderate and heavy drinking) | Depressive symptoms | Meta-analysis | 31837230 | Light drinking was associated with lower risk of depressive symptoms while heavy drinking was not associated with depressive symptoms. |
| Sleep quality | Bipolar disorder | Cohort study | 26228404 | Poor sleep quality was associated with increased risk of bipolar disorder |
| Sedentary behaviors | Clinical depression or | Meta-analysis | 25183627 | Higher sedentary behavior at baseline was associated with |

| | | | | |
|---|---|---|---|---|
| | depressive symptoms | | | higher risk of depression |
| Number of sexual partners | Depressive symptoms | Cohort study | 21590465 | Increased number of sexual partners was associated with increased risk of depressive symptoms. |
| Driving speed | Schizophrenia | Case-control | 30219605 | Patients with schizophrenia drove slower than healthy controls. |
| Age at first birth | Depressive symptoms | Cohort study | 11989963 | Delaying first birth was associated with decreased risk of depressive symptoms. |

Table S2. Detailed description of exposure phenotypes in the present study.

| Phenotypes | PMID | Sample size | Ancestry | Definition |
|---|---|---|---|---|
| **Socioeconomic status** | | | | |
| Education attainment | 30038396 | 1,131,881 | European | Education attainment was measured as number of years of schooling completed. Mean education years 16.8 ± 4.2. (SSGAC: 10k lead SNPs in the sample including 23andMe; full GWAS data was available only from the sample excluding 23andMe.) |
| Total household income before tax | 31844048 | 286,301 (Male 48.35%) | European | Using a 5-point scale corresponding to the total household income before tax: 1 being less than £18,000, 2 being £18,000-£29,999, 3 being £30,000-£51,999, 4 being £52,000-£100,000 and 5 being greater than £100,000. This 5-point scale was analyzed by treating the categories of income as a continuous variable. (UKB: top SNPs reported in the original GWAS; full GWAS data was no available.) |
| **Dietary composition** | | | | |
| Relative carbohydrate intake | 32393786 | 268,922 (Male 39.44%) | European | Carbohydrate included intake from all saccharides, while sugar included intake from mono- and disaccharides only.<br>The four dietary composition phenotypes were measured as relative intake, which could be described as an “adjusted macronutrients density”. Specifically, phenotype definition of the energy-corrected macronutrient intakes is given by<br>$corrected\ intake = \frac{energy\ from\ macronutrient}{total\ energy^{\beta}}$<br>Here, the macronutrient and total energy intake was measured in the same unit (e.g., kilocalories). β is a correction factor and detailed estimated procedure was described by Meddens et al. |
| Relative fat intake | 32393786 | 268,922 (Male 39.44%) | European | |
| Relative protein intake | 32393786 | 268,922 (Male 39.44%) | European | |
| Relative sugar intake | 32393786 | 235,391 (Male 43.32%) | European | |
| **Habitual physical activity** (UKB: top SNPs reported in the original GWAS; full GWAS data was not available.) | | | | |
| Average acceleration | 29899525 | 91,084 | European | Average acceleration was derived from up to seven days of accelerometer wear (27.98±8.14 milli-gravities). Genetic variants used in our studied were obtained from a recent MR study (PMID: 32680943). |

| | | | | |
|---|---|---|---|---|
| Moderate to vigorous physical activity (MVPA) | 29899525 | 377,234 | European | This is a self-reported phenotype measured as a continuous variable (metabolic-equivalent minutes per week: 1,650±2,084 minutes). Specifically, it was measured by taking the sum of total minutes/week of moderate physical activity (MPV) multiplied by four and the total minutes/week of VPA multiplied by eight, corresponding to their metabolic equivalents. Genetic variants proxied for MVPA were obtained from the full adjusted model without standard error (se) released. Hence, we calculated the standard error using the following formula: $se = 1/\sqrt{2N * EAF(1 - EAF)}$, where N is the sample size and EAF is the effect allele frequency of SNP (PMID: 29892602). |
| Vigorous physical activity (VPA) | 29899525 | 261,055 (98,060 cases vs. 162,995 controls) | European | This is a self-reported phenotype measured as a binary phenotype (98,060 cases with VPA for ≥ 3 days per week vs. 162,995 controls with VPA for 0 day per week). Participants were asked: "In a typical week, how many days did you do 10 minutes or more of vigorous physical activity? (These are activities that make you sweat or breathe hard such as fast cycling, aerobics, heavy lifting)". Genetic variants proxied for VPA were obtained from the full adjusted model without standard error (se) released. Hence, we calculated the standard error using the following formula: $se = \sqrt{[p \times (1 - p)]}/\sqrt{2N \times EAF(1 - EAF)}$, where p is the proportion of cases with VPA for ≥ 3 days per week, N is the sample size and EAF is the effect allele frequency of SNP (PMID: 29892602). |
| **Smoking behaviors** | | | | |
| Age of initiation of regular smoking | 30643251 | 341,427 | European | Participants were asked "At what age did you begin smoking regularly?" or "what is your current age and how long have you smoked?". |
| Cigarettes per day | 30643251 | 337,334 | European | This phenotype was measured as the average number of cigarettes smoked per day. Participants were asked "How many cigarettes do you smoke per day?" or "How many cigarettes did you smoke per day?". |
| Smoking cessation | 30643251 | 547,219 | European | This phenotype was measured as binary variable with current smokers |

| | | | | |
|---|---|---|---|---|
| | | | | coded as "2" and former smokers coded as "1". Participants were asked "Do you currently smoke? And have you ever smoked regularly?", "Do you smoke? And have you smoked over 100 cigarettes in your entire life?" |
| Smoking initiation | 30643251 | 1,232,091 | European | This phenotype was measured as binary variable with ever being a regular smoker in their life (current or former) coded as "2" and never being a regular smoker in their life coded as "1". Participants were asked "Have you smoked over 100 cigarettes over the course of your life?", "Have you ever smoked every day for at least a month?" or "Have you ever smoked regularly?". |
| **Drinking behaviors** | | | | |
| Alcohol consumption per week | 30643251 | 941,280 | European | This phenotype was measured as the average number of drinks a participant reported drinking each week, aggregated across all types of alcohol. Participants were asked "In the past week, how many alcoholic beverages did you have?" or "Thinking about the past year, on the average how many drinks did you have each week?". |
| AUDIT | 30336701 | 121,604 (Male 43.8%) | European | The Alcohol Use Disorder Identification Test (AUDIT) total score was created by taking the sum of items 1-10 for all participants. (UKB + 23andMe: 10k SNPs; full GWAS data was not publicly available) |
| **Sleeping behaviors** | | | | |
| Insomnia | 30804565 | 1,331,010 (Male 48.98%; 397,956 cases vs. 933,054 controls) | European | UK biobank phenotype:<br>Participants were asked "Do you have trouble falling asleep at night or do you wake up in the middle of the night?" and were able to choose one of the following four answers: "never/rarely", "sometimes", "usually", or "prefer not to answer". Insomnia cases were defined as participants who answered this question with "usually", while participants answering "never/rarely" or "sometimes" were defined as controls.<br>23andMe phenotype: |

| | | | | |
|---|---|---|---|---|
| | | | | Insomnia cases affirmed at least one of the following questions: "Have you ever been diagnosed with, or treated for: Insomnia?"; "Have you ever been diagnosed with, or treated for, any of the following conditions: Insomnia but not Narcolepsy, Sleep apnea or Restless leg syndrome"; "Has a doctor ever told you that you have any of these conditions: Insomnia (difficulty getting to sleep or staying asleep)?"; "Have you ever been diagnosed by a doctor with any of the following neurological conditions: Sleep disturbance"; "Do you routinely have trouble getting to sleep at night?"; "What sleep disorders have you been diagnosed with? Please select all that apply: Insomnia, trouble falling or staying asleep"; "Have you ever taken these medications? Prescription sleep aids"; "In the last 2 years, have you taken any of these medications? Prescription sleep aids"._ (UKB + 23andMe: top SNPs reported in the original GWAS; full GWAS data was only available from the sample excluding 23andMe.) |
| Long sleep duration | 30846698 | 339,926 (Male 43.9%; 34,184 case vs. 305,742 controls) | European | Long sleep duration was defined as sleeping for 9h or more relative to 7-8h sleep duration (set as controls). |
| Short sleep duration | 30846698 | 411,934 (Male 46.9%; 106,192 cases vs. 305,742 controls). | European | Short sleep duration was defined as sleeping for 6h or less relative to 7-8h sleep duration (set as controls). |
| Sleep duration | 30846698 | 446,118 (Male 45.9%) | European | Sleep duration was treated as a continuous variable. Participants were asked: About how many hours sleep do you get in every 24h? (please include naps), with responses in hour increments. Extreme responses of |

| | | | | |
|---|---|---|---|---|
| | | | | less than 3 h or more than 18h were excluded and "Do not know" or "Prefer not to answer responses" were set to missing. Mean sleep duration was 7.2±1.1 hours. |
| **Leisure sedentary behaviors** (UKB: top SNPs reported in the original GWAS; full GWAS data was not publicly available) | | | | |
| Computer use time | 32317632 | 422,218 (Male 45.7%) | European | Participants were asked "In a typical day, how many hours do you spend using the computer?". Average time using the computer was 1.0 ± 1.2h. |
| Television watching time | 32317632 | 422,218 (Male 45.7%) | European | Participants were asked "In a typical day, how many hours do you spend watching TV?". Average time watching TV was 2.8 ± 1.5h. |
| Drive time | 32317632 | 422,218 (Male 45.7%) | European | Participants were asked "In a typical day, how many hours do you spend driving?". Average time driving was 0.9 ± 1.0h. |
| **Risky behaviors** (SSGAC: top SNPs reported in the original GWAS; full GWAS data for adventurousness was not available) | | | | |
| Adventurousness | 30643258 | 557,923 | European | Participants were asked "if forced to choose, would you consider yourself to be more cautious or more adventurous? 1) Very cautious /2) Somewhat cautious /3) Neither /4) Somewhat adventurous /5) Very adventurous". |
| Automobile speeding propensity | 30643258 | 404,291 | European | Participants were asked "How often do you drive faster than the speed limit on the motorway?". Response options: 1) Never/rarely, 2) Sometimes, 3) Often, 4) Most of the time, and also 5) Do not drive on the motorway. Average normalized automobile speeding propensity was 0.04 ± 0.82. Those not driving on the motorway were dropped. |
| General risk to tolerance | 30643258 | 939,908 | European | The UKB measurement of general risk tolerance was based on the question "Would you describe yourself as someone who takes risks? Yes/No". The 23andMe measurement was based on a question about overall comfort taking risks, with five response options ranging from "very comfortable'" to "very uncomfortable". |
| Number of sexual partners | 30643258 | 370,711 | European | Participants were asked "About how many sexual partners have you had in your lifetime?". If respondents reported more than 99 lifetime sexual partners, they were asked to confirm their responses. Average number of |

| | | | | |
|---|---|---|---|---|
| | | | | sexual partners was 0.03 ± 0.94. |
| **Reproductive behaviors** (SSGAC: top SNPs reported in the original GWAS; full GWAS data was only available from the sample excluding 23andMe.) | | | | |
| Age at first birth (AFB) | 27798627 | 251,151 (Male 20.33%) | European | Age at first birth (AFB):<br>Self-reported age when subjects had their first child. In most cohorts this was asked directly like "How old were you when you had your first child?". Besides, it could also be calculated based on survey questions such as the date of birth of the subject and date of birth of the first child. The average age was 26.8 ± 4.78 years.<br>Number of children ever born (NEB):<br>Self-reported number of children. Participants were asked directly (e.g. "How many children do you have?" or "How many natural (biological) children have you ever had, that is, all children who were born alive?", or "How many children have you had - not counting any step, adopted, or foster children, or any who were stillborn?") . Besides, it could also be calculated based on survey questions such as pregnancy histories and outcomes, number of deliveries and so on. The average number was 2.3 ± 1.43 children.<br>Standard error (se) of estimated genetic effect on AFB and NEB was not released by Barban et al. As AFB and NEB were analyzed as continuous variables, standard error was calculated using the following formula: $se = 1/\sqrt{2N * EAF(1 - EAF)}$, where N is the sample size and EAF is the effect allele frequency of SNP (PMID: 29892602). |
| Number of children ever born (NEB) | 27798627 | 343,072 (Male 31.57%) | European | |

SSGAC, Social Science Genetic Association Consortium; SNP, single nucleotide polymorphism; GWAS, genome-wide association study; UKB, United Kingdom Biobank;

Table S3. Detailed descriptions for GWAS meta-analysis of 3 mental disorders.

| **Mental disorders** | Bipolar disorder | Major depressive disorder | Schizophrenia |
|---|---|---|---|
| **Samples** | PGC1 and PGC2 (totally 32 samples) | PGC29, deCODE, Generation Scotland, GERA, and iPSYCH (totally 33 samples) | PGC1, new CC and East Asia (totally 49 samples) |
| **Total sample sizes** | 20,352 cases/31,358 controls | 45,396 cases/97,250 controls | 34,241 cases/45,604 controls |
| **Percentage of European** | 100% | 100% | 93.5% |
| **Diagnosis criteria** | Using structured diagnostic instruments, a lifetime diagnosis of bipolar disorder was established on the basis of international consensus criteria (DSM-IV or ICD-9/10). Assessments came from trained interviewers, clinician-administered checklists or medical record review. | Cases ascertainment included structured diagnostic interviews, national inpatient electronic records and Kaiser Permanente Northern California Healthcare electronic medical records (1995-2013). Lifetime major depressive disorder was diagnosed according to DSM-III/IV or ICD-9/10. | Diagnosis of schizophrenia was established on the basis of DSM-IV or ICD-10. All participating cohorts were further included for GWAS meta-analysis based on a questionnaire covering assessment protocol and associated quality control procedures. |

GWAS, genome-wide association study; PGC, Psychiatric Genomics Consortium. New CC, case-control samples not part of a discovery portion of a prior PGC mega-analysis; iPSYCH, Integrative Psychiatric Research; DSM, Diagnostic and Statistical Manual of Mental Disorders; ICD, International Classification of Diseases.

Table S4. Details of SNPs filtering procedures.

| Exposure phenotypes | Number of SNPs available[1] | SNP filtering (number of SNPs remained[2]) | | | | | | | | | |
|---|---|---|---|---|---|---|---|---|---|---|---|
| | | | BIP | | | MDD | | | SCZ | | |
| | | Step 1 | Step 2 | Step 3 | Step 4 | Step 2 | Step 3 | Step 4 | Step 2 | Step 3 | Step 4 |
| **Socioeconomic status** | | | | | | | | | | | |
| Educational attainment | 393 | 386 | 385 | 371 | 364 | 385 | 371 | 369 | 380 | 367 | 347 |
| Total household income before tax | 27 | 25 | 25 | 24 | 21 | 25 | 24 | 24 | 24 | 23 | 19 |
| **Dietary composition** | | | | | | | | | | | |
| Relative carbohydrate intake | 11 | 7 | 6 | 6 | 5 | 6 | 6 | 6 | 6 | 6 | 6 |
| Relative fat intake | 4 | 3 | 3 | 3 | 3 | 3 | 3 | 3 | 3 | 3 | 3 |
| Relative protein intake | 7 | 7 | 7 | 7 | 5 | 7 | 7 | 7 | 7 | 7 | 5 |
| Relative sugar intake | 8 | 5 | 5 | 4 | 4 | 5 | 4 | 4 | 5 | 4 | 4 |
| **Habitual physical activity** | | | | | | | | | | | |
| Average acceleration | 8 | 8 | 8 | 7 | 7 | 8 | 7 | 7 | 8 | 7 | 5 |
| Moderate to vigorous physical activity | 7 | 6 | 5 | 5 | 4 | 5 | 5 | 4 | 5 | 5 | 4 |
| Vigorous physical activity | 4 | 4 | 4 | 4 | 3 | 4 | 4 | 4 | 4 | 4 | 3 |
| **Smoking behaviors** | | | | | | | | | | | |
| Age of initiation of regular smoking | 9 | 9 | 9 | 7 | 7 | 9 | 7 | 7 | 9 | 7 | 6 |
| Number of cigarettes per day | 38 | 37 | 37 | 37 | 34 | 37 | 37 | 37 | 35 | 35 | 27 |
| Smoking cessation | 15 | 14 | 14 | 14 | 13 | 14 | 14 | 13 | 13 | 13 | 11 |
| Smoking initiation | 202 | 195 | 195 | 188 | 184 | 195 | 188 | 183 | 190 | 183 | 174 |
| **Drinking behaviors** | | | | | | | | | | | |
| Alcohol consumption per week | 71 | 66 | 66 | 64 | 62 | 66 | 65 | 63 | 64 | 63 | 56 |
| Alcohol Use Disorder Identification Test (AUDIT) | 6 | 3 | 3 | 3 | 3 | 3 | 3 | 3 | 3 | 3 | 3 |
| **Sleeping behaviors** | | | | | | | | | | | |
| Insomnia | 158 | 155 | 155 | 148 | 146 | 155 | 148 | 147 | 152 | 145 | 135 |
| Long sleep duration | 8 | 8 | 7 | 7 | 7 | 7 | 7 | 7 | 7 | 7 | 7 |
| Short sleep duration | 25 | 21 | 21 | 19 | 4 | 21 | 19 | 17 | 21 | 19 | 16 |

| | | | | | | | | | | | |
|---|---|---|---|---|---|---|---|---|---|---|---|
| Sleep duration | 63 | 61 | 60 | 56 | 54 | 60 | 56 | 54 | 59 | 55 | 50 |
| **Leisure sedentary behaviors** | | | | | | | | | | | |
| Computer use time | 22 | 20 | 20 | 20 | 16 | 20 | 20 | 19 | 19 | 19 | 18 |
| Television watching time | 95 | 91 | 89 | 84 | 81 | 90 | 85 | 85 | 87 | 82 | 79 |
| Drive time | 4 | 4 | 4 | 4 | 3 | 4 | 4 | 4 | 3 | 3 | 3 |
| **Risky behaviors** | | | | | | | | | | | |
| Adventurousness | 108 | 103 | 102 | 97 | 96 | 102 | 97 | 97 | 102 | 97 | 90 |
| Automobile speeding propensity | 31 | 28 | 28 | 28 | 24 | 28 | 28 | 28 | 27 | 27 | 27 |
| General risk tolerance | 80 | 73 | 71 | 68 | 66 | 71 | 68 | 67 | 67 | 64 | 59 |
| Number of sexual partners | 77 | 73 | 73 | 71 | 68 | 73 | 71 | 69 | 69 | 67 | 62 |
| **Reproductive behaviors** | | | | | | | | | | | |
| Age at first birth | 9 | 9 | 9 | 9 | 9 | 9 | 9 | 9 | 9 | 9 | 7 |
| Number of children ever born | 2 | 2 | 2 | 2 | 2 | 2 | 2 | 2 | 2 | 2 | 2 |

SNP, single nucleotide polymorphisms; BIP, bipolar disorder; MDD, major depressive disorder; SCZ, schizophrenia.

[1] Number of independent SNPs ($p < 5 \times 10^{-8}$) within each phenotype at a cut-off of linkage disequilibrium $r^2 \leq 0.001$.

[2] SNPs filtering steps:

Step 1, we excluded SNPs associated with more than one phenotype.

Step 2, we extracted exposure-SNPs from the outcomes and excluded the SNPs meeting the following items: 1) SNPs associated with outcomes at genome-wide significance; 2) SNPs absent in the outcome GWAS data without appropriate proxies available.

Step 3, we harmonized the SNPs of from exposure and outcome to exclude SNPs being palindromic with intermediate allele frequencies based on the allele frequency over 0.42 using the harmonization function in the "Two-sample MR" R package.

Step 4, we conducted MR-PRESSO to exclude SNPs with potential pleiotropy using "MR-PRESSO" R package.

Table S5. List of genetic variants associated with more than one phenotype.

| SNPs | Exposures |
|---|---|
| rs1008078 | educational attainment, smoking initiation |
| rs10189857 | educational attainment, television watching time |
| rs1104608 | relative carbohydrate intake, alcohol consumption per week |
| rs11076962 | educational attainment, smoking initiation |
| rs11128203 | smoking initiation, automobile speeding propensity |
| rs11588857 | educational attainment, total household income before tax |
| rs11940694 | alcohol consumption per week, Alcohol Use Disorder Identification Test |
| rs12151248 | educational attainment, total household income before tax |
| rs12567114 | short sleep duration, sleep duration |
| rs1260326 | alcohol consumption per week, Alcohol Use Disorder Identification Test |
| rs13107325 | alcohol consumption per week, television watching time, short sleep duration |
| rs1531518 | general risk tolerance, number of sexual partners |
| rs17005118 | insomnia, short sleep duration |
| rs205024 | short sleep duration, sleep duration |
| rs2239030 | adventurousness, general risk tolerance |
| rs2279829 | smoking initiation, number of sexual partners |
| rs2472297 | relative carbohydrate intake, alcohol consumption per week |
| rs42210 | educational attainment, television watching time |
| rs429358 | moderate to vigorous physical activity, insomnia |
| rs4702 | insomnia, number of sexual partners |
| rs55745410 | adventurousness, general risk tolerance |
| rs56113850 | number of cigarettes per day, smoking cessation |
| rs58400863 | smoking initiation, general risk tolerance |
| rs62062288 | alcohol Use Disorder Identification Test, automobile speeding propensity |
| rs62519839 | adventurousness, general risk tolerance |
| rs67361341 | adventurousness, general risk tolerance |

| | |
|---|---|
| rs6874731 | smoking initiation, general risk tolerance |
| rs7012814 | relative fat intake, Relative sugar intake |
| rs72780746 | smoking initiation, number of sexual partners |
| rs8097672 | relative carbohydrate intake, relative sugar intake |
| rs838144 | relative carbohydrate intake, relative sugar intake |
| rs9372625 | computer use time, automobile speeding propensity |
| rs9556958 | educational attainment, adventurousness |
| rs984409 | computer use time, television watching time |

SNP, single nucleotide polymorphisms.

Table S6. Descriptions of the Mendelian randomization (MR) methods.

| Mendelian randomization methods | Intercept | Breakdown levels | Assumptions and comments |
|---|---|---|---|
| Inverse variance weighted (IVW) | Zero | 0% | Instrumental variants (IVs) should meet the Instrument Strength Independent of Direct Effects (InSIDE) assumption. The method assumes that all the variants are valid. Even one invalid IV can bias the effect estimate. This method is the most powerful and precise. |
| MR-Egger regression | Non-zero | 100% | IVs should meet the InSIDE assumption. This method allows all IVs are invalid but it is substantially less efficient than IVW and median-based methods. A non-zero intercept derived from Egger regression can be used to determine whether there is directional pleiotropy detected. |
| Weighted median | Zero | 50% | This method allows no more than 50% of invalid IVs and the efficiency is similar to that of IVW. |
| Weighted-mode based estimation | Zero | 50-100% | This method allows more than 50% of invalid IVs. The efficiency is smaller the IVW and weighted median methods, but is larger than that of MR Egger regression. |

Table S7. Mendelian randomization estimates for the association of socioeconomic status/individual behaviors with 3 mental disorder derived from different models.

| Exposures | Outcomes | Number of SNPs | Number of proxies[1] | Inverse variance weighted | | MR-Egger | | Weighted median | | Weighted mode | |
|---|---|---|---|---|---|---|---|---|---|---|---|
| | | | | OR (95% CI) | *p* | OR (95% CI) | *p* | OR (95% CI) | *p* | OR (95% CI) | *p* |
| Educational attainment | BIP | 364 | 0 | 1.53 (1.28, 1.82) | 2.91E-06 | 1.06 (0.55,1.74) | 0.87 | 1.38 (1.10,3.01) | 4.64E-03 | 1.11 (0.60,1.81) | 0.75 |
| Total household income before tax | BIP | 21 | 3 | 1.06 (0.61, 1.83) | 0.85 | 0.31 (2.46E-04, 399.18) | 0.75 | 1.12 (0.52, 2.40) | 0.78 | 1.04 (0.25, 4.36) | 0.95 |
| Relative carbohydrate intake | BIP | 5 | 0 | 1.29 (0.64, 2.62) | 0.48 | 0.92 (8.81E-05, 9.63E+03) | 0.99 | 1.66 (0.65, 4.22) | 0.29 | 1.78 (0.48, 6.64) | 0.44 |
| Relative fat intake | BIP | 3 | 0 | 0.70 (0.30, 1.61) | 0.40 | 1.01 (0.17, 5.98) | 0.99 | 1.05 (0.57, 1.96) | 0.87 | 1.06 (0.52, 2.17) | 0.89 |
| Relative protein intake | BIP | 5 | 0 | 1.76 (0.78, 3.95) | 0.17 | 7.65 (0.06, 910.90) | 0.47 | 2.21 (0.97, 5.05) | 0.06 | 2.11 (0.73, 6.06) | 0.24 |
| Relative sugar intake | BIP | 4 | 0 | 0.71 (0.33, 1.55) | 0.39 | 3.77E-08 (9.61E-21, 1.48E+05) | 0.37 | 0.63 (0.25, 1.58) | 0.33 | 0.60 (0.16, 2.23) | 0.50 |
| Average acceleration | BIP | 7 | 1 | 1.04 (0.98, 1.12) | 0.21 | 0.97 (0.71, 1.31) | 0.85 | 1.04(0.97,1.11) | 0.24 | 1.03 (0.92, 1.14) | 0.64 |
| Moderate to vigorous physical activity | BIP | 4 | 0 | 3.64 (0.89, 14.80) | 0.07 | 3.65E+08 (1.38, 9.68E+16) | 0.18 | 2.02 (0.51, 8.06) | 0.32 | 1.29 (0.17, 9.58) | 0.82 |
| Vigorous physical activity | BIP | 3 | 0 | 0.94 (0.63, 1.39) | 0.75 | 2.45 (0.23, 26.55) | 0.59 | 1.03 (0.63, 1.71) | 0.90 | 1.11 (0.60, 2.10) | 0.77 |
| Age of initiation of regular smoke | BIP | 7 | 0 | 0.64 (0.33, 1.24) | 0.19 | 0.78 (0.03, 17.83) | 0.88 | 0.68 (0.29, 1.56) | 0.36 | 0.43 (0.12, 1.49) | 0.23 |
| Number of cigarettes per day | BIP | 34 | 0 | 1.23 (0.90, 1.68) | 0.19 | 1.29 (0.76, 2.21) | 0.35 | 1.29 (0.98, 1.71) | 0.07 | 1.32 (0.99, 1.75) | 0.07 |
| Smoking cessation | BIP | 13 | 0 | 1.44 (1.04, 2.00) | 0.03 | 1.33 (0.47, 3.75) | 0.60 | 1.31 (0.87, 1.98) | 0.19 | 1.24 (0.69, 2.21) | 0.48 |
| Smoking initiation | BIP | 184 | 0 | 1.62 (1.39, 1.88) | 2.78E-10 | 1.16 (0.65, 2.09) | 0.61 | 1.60 (1.34, 1.92) | 3.44E-07 | 2.25 (1.24, 4.07) | 8.29E-03 |
| Alcohol consumption per week | BIP | 62 | 0 | 1.31 (0.81, 2.13) | 0.27 | 1.04 (0.47, 2.29) | 0.93 | 1.17 (0.75, 1.83) | 0.49 | 1.08 (0.67, 1.75) | 0.74 |
| Alcohol Use Disorders Identification Test | BIP | 3 | 0 | 0.61 (0.04, 10.24) | 0.73 | 1.65 (3.83E-03, 711.72) | 0.90 | 1.24 (0.13, 11.72) | 0.85 | 1.59 (0.13, 19.11) | 0.75 |
| Insomnia | BIP | 146 | 0 | 1.18 (1.08, 1.28) | 1.30E-04 | 1.21 (0.88, 1.66) | 0.24 | 1.17 (1.06, 1.28) | 2.08E-03 | 1.20 (0.91, 1.60) | 0.20 |
| Long sleep duration | BIP | 7 | 1 | 0.99 (0.81, 1.20) | 0.89 | 1.12 (0.62, 2.02) | 0.72 | 1.01 (0.79, 1.28) | 0.94 | 1.02 (0.72, 1.42) | 0.93 |
| Short sleep duration | BIP | 4 | 0 | 0.40 (0.23,0.70) | 1.23E-03 | 0.42 (0.02, 9.30) | 0.64 | 0.48 (0.25, 0.93) | 0.03 | 0.58 (0.22, 1.50) | 0.34 |
| Sleep duration | BIP | 54 | 1 | 1.007 (1.001, 1.013) | 0.02 | 0.993 (0.972, 1.013) | 0.49 | 1.007 (0.999, 1.015) | 0.06 | 1.006 (0.995, 1.017) | 0.31 |

| | | | | | | | | | | | |
|---|---|---|---|---|---|---|---|---|---|---|---|
| Computer use time | BIP | 16 | 0 | 1.57 (0.91, 2.70) | 0.11 | 480.30 (7.03, 3.28E+04) | 0.01 | 1.13 (0.57, 2.21) | 0.73 | 0.87 (0.21, 3.58) | 0.85 |
| Television watching time | BIP | 81 | 0 | 0.87 (0.67, 1.14) | 0.31 | 0.72 (0.18, 2.87) | 0.64 | 0.98 (0.71, 1.33) | 0.88 | 1.56 (0.62, 3.94) | 0.35 |
| Drive time | BIP | 3 | 0 | 0.50 (0.17, 1.49) | 0.21 | 2.49 (1.23E-04, 5.04E+04) | 0.89 | 0.62 (0.16, 2.40) | 0.49 | 0.72 (0.16, 3.26) | 0.71 |
| Adventurousness | BIP | 96 | 0 | 2.12 (1.58, 2.84) | 4.22E-07 | 2.23 (0.70, 7.10) | 0.18 | 2.13 (1.49, 3.04) | 3.05E-05 | 2.55 (1.30, 4.97) | 7.39E-03 |
| Automobile speeding propensity | BIP | 24 | 0 | 0.78 (0.49, 1.23) | 0.29 | 0.37 (0.04, 3.18) | 0.37 | 0.80 (0.46, 1.39) | 0.43 | 0.77 (0.31, 1.93) | 0.58 |
| General risk tolerance | BIP | 66 | 0 | 3.63 (2.36, 5.58) | 4.03E-09 | 27.61 (3.61, 208.25) | 2.14E-03 | 3.42 (1.97, 5.93) | 1.24E-05 | 10.44 (2.46, 44.35) | 2.27E-03 |
| Number of sexual partners | BIP | 68 | 2 | 1.92 (1.45, 2.53) | 4.08E-06 | 0.89 (0.23, 3.48) | 0.87 | 1.51 (1.07, 2.12) | 0.02 | 1.13 (0.50, 2.57) | 0.77 |
| Age at first birth | BIP | 9 | 0 | 1.03 (0.89, 1.21) | 0.67 | 1.86 (0.39, 8.91) | 0.46 | 1.00 (0.85, 1.16) | 0.96 | 0.99 (0.75, 1.31) | 0.94 |
| Number of children ever born | BIP | 2 | 0 | 0.89 (0.13, 5.61) | 0.87 | NA | NA | NA | NA | NA | NA |
| Educational attainment | MDD | 369 | 0 | 0.72 (0.64, 0.80) | 1.43E-09 | 0.73 (0.4, 1.08) | 0.11 | 0.73 (0.63, 0.84) | 8.70E-06 | 0.61 (0.40, 0.92) | 0.02 |
| Total household income before tax | MDD | 24 | 3 | 0.61 (0.42, 0.87) | 7.41E-03 | 1.19 (0.09, 15.31) | 0.90 | 0.67 (0.42, 1.07) | 0.09 | 0.73 (0.33, 1.60) | 0.44 |
| Relative carbohydrate intake | MDD | 6 | 0 | 0.50 (0.32, 0.80) | 4.02E-03 | 0.48 (9.82E-04, 230.89) | 0.83 | 0.40 (0.23, 0.71) | 1.82E-03 | 0.36 (0.14, 0.92) | 0.09 |
| Relative fat intake | MDD | 3 | 0 | 1.11(0.76,1.62) | 0.59 | 0.92 (0.42, 2.00) | 0.86 | 1.04 (0.70, 1.55) | 0.85 | 1.02 (0.66, 1.60) | 0.92 |
| Relative protein intake | MDD | 7 | 0 | 1.19 (0.80, 1.76) | 0.40 | 0.35 (0.08, 1.50) | 0.22 | 1.04 (0.67, 1.62) | 0.87 | 0.95 (0.54, 1.65) | 0.86 |
| Relative sugar intake | MDD | 4 | 0 | 0.50 (0.31, 0.82) | 5.97E-03 | 0.98 (3.02E-09, 3.21E+08) | 0.999 | 0.53 (0.29, 0.95) | 0.03 | 0.56 (0.24, 1.35) | 0.29 |
| Average acceleration | MDD | 7 | 1 | 0.97 (0.94, 1.00) | 0.056 | 0.93 (0.82, 1.06) | 0.31 | 0.97 (0.94, 1.01) | 0.19 | 1.00 (0.94, 1.06) | 0.95 |
| Moderate to vigorous physical activity | MDD | 4 | 0 | 0.50 (0.25, 1.01) | 0.053 | 1.01E-03 (1.92E-08, 53.78) | 0.34 | 0.50 (0.22, 1.13) | 0.10 | 0.36 (0.12, 1.11) | 0.17 |
| Vigorous physical activity | MDD | 4 | 0 | 0.96 (0.78, 1.19) | 0.72 | 0.33 (0.08, 1.27) | 0.25 | 0.94 (0.74, 1.20) | 0.63 | 0.85 (0.61, 1.20) | 0.42 |
| Age of initiation of regular smoke | MDD | 7 | 0 | 0.50 (0.30, 0.85) | 0.01 | 3.69 (0.44, 30.63) | 0.28 | 0.69 (0.38, 1.26) | 0.23 | 0.80 (0.34, 1.86) | 0.62 |
| Number of cigarettes per day | MDD | 37 | 1 | 1.13 (0.94, 1.36) | 0.20 | 0.83 (0.61, 1.12) | 0.23 | 0.92 (0.77, 1.10) | 0.36 | 0.96 (0.79, 1.15) | 0.64 |
| Smoking cessation | MDD | 13 | 0 | 1.26 (1.03, 1.55) | 0.03 | 1.05 (0.57, 1.93) | 0.89 | 1.19 (0.91, 1.55) | 0.20 | 1.17 (0.83, 1.65) | 0.38 |
| Smoking initiation | MDD | 183 | 1 | 1.52 (1.39,1.66) | 3.28E-19 | 0.92 (0.64, 1.31) | 0.63 | 1.44 (1.28,1.61) | 1.39E-10 | 1.43 (1.06, 1.94) | 0.02 |

| | | | | | | | | | | | |
|---|---|---|---|---|---|---|---|---|---|---|---|
| Alcohol consumption per week | MDD | 63 | 0 | 1.12 (0.89,1.43) | 0.34 | 0.93 (0.62, 1.41) | 0.73 | 0.94 (0.69,1.30) | 0.72 | 0.95 (0.70, 1.28) | 0.72 |
| Alcohol Use Disorders Identification Test | MDD | 3 | 0 | 2.40 (0.67, 8.58) | 0.18 | 3.26 (0.39, 27.44) | 0.47 | 2.96 (0.67, 13.05) | 0.15 | 3.23 (0.57, 18.32) | 0.32 |
| Insomnia | MDD | 147 | 0 | 1.26 (1.20, 1.32) | 1.01E-22 | 1.08 (0.91, 1.29) | 0.37 | 1.28 (1.20, 1.36) | 3.79E-15 | 1.29 (1.08, 1.53) | 5.27E-03 |
| Long sleep duration | MDD | 7 | 1 | 0.95 (0.79, 1.14) | 0.57 | 1.20 (0.70, 2.06) | 0.54 | 0.98 (0.82, 1.18) | 0.84 | 1.03 (0.78, 1.35) | 0.84 |
| Short sleep duration | MDD | 17 | 1 | 1.22 (1.00, 1.49) | 0.049 | 0.69 (0.28, 1.68) | 0.43 | 1.25 (1.00, 1.56) | 0.051 | 1.27 (0.87, 1.85) | 0.23 |
| Sleep duration | MDD | 54 | 0 | 0.999 (0.995, 1.004) | 0.80 | 0.999 (0.98, 1.01) | 0.95 | 1.001 (0.996, 1.005) | 0.78 | 1.002 (0.994, 1.009) | 0.61 |
| Computer use time | MDD | 19 | 0 | 1.23 (0.82, 1.85) | 0.31 | 0.82 (0.03, 26.67) | 0.94 | 1.22 (0.79, 1.86) | 0.37 | 1.34 (0.69, 2.61) | 0.39 |
| Television watching time | MDD | 85 | 2 | 1.04 (0.89, 1.23) | 0.61 | 0.70 (0.30, 1.63) | 0.41 | 1.02 (0.84, 1.25) | 0.83 | 1.07 (0.61, 1.88) | 0.81 |
| Drive time | MDD | 4 | 0 | 0.75 (0.40, 1.41) | 0.37 | 7.81 (0.03, 1961.79) | 0.54 | 0.83 (0.39, 1.77) | 0.63 | 1.16 (0.42, 3.16) | 0.79 |
| Adventurousness | MDD | 97 | 0 | 1.04 (0.88, 1.23) | 0.62 | 0.66 (0.35, 1.25) | 0.21 | 0.93 (0.74, 1.18) | 0.56 | 0.93 (0.64, 1.33) | 0.68 |
| Automobile speeding propensity | MDD | 28 | 0 | 0.55 (0.40, 0.76) | 2.26E-04 | 0.22 (0.05, 0.92) | 0.049 | 0.61 (0.42, 0.89) | 0.01 | 0.72 (0.37, 1.42) | 0.35 |
| General risk tolerance | MDD | 67 | 0 | 1.25 (0.96, 1.62) | 0.09 | 1.47 (0.42, 5.20) | 0.55 | 1.25 (0.88, 1.77) | 0.22 | 1.12 (0.49, 2.59) | 0.79 |
| Number of sexual partners | MDD | 69 | 1 | 1.40 (1.16, 1.69) | 5.04E-04 | 1.15 (0.44, 3.00) | 0.78 | 1.42 (1.13, 1.78) | 2.35E-03 | 1.36 (0.74, 2.50) | 0.33 |
| Age at first birth | MDD | 9 | 0 | 0.82 (0.75, 0.90) | 2.17E-05 | 0.52 (0.21, 1.30) | 0.20 | 0.79 (0.72, 0.88) | 7.40E-06 | 0.76 (0.65, 0.90) | 0.01 |
| Number of children ever born | MDD | 2 | 0 | 2.43 (0.79, 7.52) | 0.12 | NA | NA | NA | NA | NA | NA |
| Educational attainment | SCZ | 347 | 0 | 1.06 (0.90, 1.26) | 0.46 | 0.79 (0.43, 1.45) | 0.45 | 1.05 (0.86, 1.27) | 0.64 | 1.38 (0.67, 2.86) | 0.39 |
| Total household income before tax | SCZ | 19 | 2 | 0.32 (0.16, 0.67) | 2.28E-03 | 0.42 (8.83E-04, 201.58) | 0.79 | 0.29 (0.15, 0.56) | 2.54E-04 | 0.26 (0.10, 0.66) | 0.01 |
| Relative carbohydrate intake | SCZ | 6 | 0 | 1.48 (0.77, 2.85) | 0.24 | 4.20 (8.19E-04, 2.15E+04) | 0.76 | 1.98 (1.01, 3.89) | 0.04 | 2.61 (0.79, 8.69) | 0.18 |
| Relative fat intake | SCZ | 3 | 0 | 0.63 (0.18, 2.17) | 0.46 | 0.38 (0.03, 4.94) | 0.59 | 0.53 (0.34, 0.82) | 5.10E-03 | 0.51 (0.32, 0.81) | 0.10 |
| Relative protein intake | SCZ | 5 | 0 | 0.75 (0.45, 1.26) | 0.28 | 2.01 (3.88E-03, 1.04E+03) | 0.84 | 0.73 (0.40, 1.34) | 0.31 | 0.74 (0.33, 1.62) | 0.49 |
| Relative sugar intake | SCZ | 4 | 0 | 1.30 (0.72, 2.34) | 0.39 | 9.89 (9.22E-10, 1.06E+11) | 0.86 | 1.38 (0.70, 2.69) | 0.35 | 1.49 (0.59, 3.72) | 0.46 |
| Average acceleration | SCZ | 5 | 0 | 0.99 (0.95, 1.04) | 0.76 | 0.93 (0.78, 1.10) | 0.44 | 0.99 (0.93, 1.05) | 0.74 | 0.97 (0.90, 1.06) | 0.57 |
| Moderate to vigorous physical activity | SCZ | 4 | 0 | 3.01 (1.30, 6.95) | 9.83E-03 | 0.25 (6.86E-08, | 0.87 | 2.72 (1.03, 7.17) | 0.04 | 2.52 (0.70, 9.01) | 0.25 |

| | | | | | | | | | | | |
|---|---|---|---|---|---|---|---|---|---|---|---|
| | | | | | | 9.04E+05) | | | | | |
| Vigorous physical activity | SCZ | 3 | 0 | 1.16 (0.88, 1.53) | 0.29 | 0.85 (0.14, 5.16) | 0.89 | 1.21 (0.89, 1.63) | 0.22 | 1.26 (0.85, 1.86) | 0.37 |
| Age of initiation of regular smoke | SCZ | 6 | 0 | 0.89 (0.50, 1.56) | 0.68 | 1.00 (5.10E-03, 196.05) | 0.9999 | 0.75 (0.36, 1.57) | 0.44 | 0.48 (0.16, 1.43) | 0.25 |
| Number of cigarettes per day | SCZ | 27 | 0 | 1.65 (1.28, 2.13) | 1.26E-04 | 1.67 (1.08, 2.58) | 0.03 | 1.71 (1.36, 2.14) | 3.06E-06 | 1.67 (1.33, 2.09) | 1.47E-04 |
| Smoking cessation | SCZ | 11 | 0 | 1.18 (0.94, 1.50) | 0.16 | 0.97 (0.52, 1.79) | 0.92 | 1.18 (0.85, 1.63) | 0.33 | 1.20 (0.76, 1.87) | 0.45 |
| Smoking initiation | SCZ | 174 | 1 | 1.64 (1.43, 1.88) | 2.08E-12 | 1.39 (0.78, 2.48) | 0.26 | 1.53 (1.30, 1.80) | 2.01E-07 | 1.25 (0.71, 2.21) | 0.45 |
| Alcohol consumption per week | SCZ | 56 | 0 | 1.43 (1.06, 1.93) | 0.02 | 1.28 (0.81, 2.02) | 0.29 | 1.34 (0.99, 1.81) | 0.06 | 1.28 (0.95, 1.73) | 0.11 |
| Alcohol Use Disorders Identification Test | SCZ | 3 | 0 | 0.61 (0.14, 2.62) | 0.51 | 0.59 (0.05, 6.27) | 0.74 | 0.60 (0.11, 3.26) | 0.56 | 0.59 (0.09, 3.73) | 0.63 |
| Insomnia | SCZ | 135 | 0 | 1.11 (1.03, 1.20) | 4.56E-03 | 0.77 (0.58, 1.02) | 0.07 | 1.07 (0.99, 1.17) | 0.10 | 0.88 (0.67, 1.17) | 0.39 |
| Long sleep duration | SCZ | 7 | 1 | 1.41 (1.15, 1.73) | 9.79E-04 | 1.34 (0.69, 2.60) | 0.42 | 1.33 (1.04, 1.68) | 0.02 | 1.29 (0.93, 1.78) | 0.18 |
| Short sleep duration | SCZ | 16 | 0 | 0.81 (0.59, 1.10) | 0.18 | 0.42 (0.11, 1.65) | 0.24 | 0.70 (0.53, 0.93) | 0.01 | 0.67 (0.43, 1.06) | 0.11 |
| Sleep duration | SCZ | 50 | 1 | 1.006 (1.000, 1.012) | 0.02 | 1.01 (0.99, 1.03) | 0.32 | 1.008 (1.002, 1.014) | 6.68E-03 | 1.005 (0.996, 1.014) | 0.33 |
| Computer use time | SCZ | 18 | 0 | 0.72 (0.44, 1.18) | 0.19 | 3.19 (0.04, 228.42) | 0.60 | 1.08 (0.62, 1.87) | 0.79 | 1.41 (0.47, 4.27) | 0.55 |
| Television watching time | SCZ | 79 | 2 | 0.88 (0.68, 1.14) | 0.34 | 0.52 (0.14, 1.99) | 0.34 | 0.81 (0.61, 1.07) | 0.13 | 0.83 (0.41, 1.67) | 0.60 |
| Drive time | SCZ | 3 | 0 | 0.18 (0.03, 1.19) | 0.08 | 4.61E+20 (7.43E-10, 2.86E+50) | 0.40 | 0.25 (0.07, 0.97) | 0.045 | 0.32 (0.05, 2.02) | 0.35 |
| Adventurousness | SCZ | 90 | 0 | 1.45 (1.11, 1.89) | 6.73E-03 | 0.85 (0.30, 2.42) | 0.76 | 1.25 (0.93, 1.67) | 0.13 | 0.85 (0.49, 1.46) | 0.55 |
| Automobile speeding propensity | SCZ | 27 | 0 | 0.42 (0.27, 0.65) | 9.88E-05 | 0.14 (0.02, 1.05) | 0.07 | 0.52 (0.33, 0.83) | 6.22E-03 | 0.51 (0.22, 1.16) | 0.12 |
| General risk tolerance | SCZ | 59 | 0 | 1.54 (1.00, 2.37) | 0.0502 | 3.92 (0.52, 29.55) | 0.19 | 1.61 (0.99, 2.61) | 0.054 | 1.67 (0.42, 6.65) | 0.47 |
| Number of sexual partners | SCZ | 62 | 1 | 2.48 (1.92, 3.21) | 3.17E-12 | 3.61 (0.92, 14.24) | 0.07 | 2.17 (1.62, 2.92) | 2.72E-07 | 1.73 (0.84, 3.55) | 0.14 |
| Age at first birth | SCZ | 7 | 0 | 1.06 (0.95, 1.20) | 0.30 | 3.30 (1.10, 9.88) | 0.09 | 1.09 (0.95, 1.26) | 0.23 | 1.06 (0.84, 1.32) | 0.65 |
| Number of children ever born | SCZ | 2 | 0 | 1.64 (0.77, 3.47) | 0.20 | NA | NA | NA | NA | NA | NA |

BIP, bipolar disorder; MDD, major depressive disorder; SCZ, schizophrenia; NA, not applicable.

[1] Proxies details:

Total household income before tax to BIP/MDD: rs12647647 was proxied by rs12649553 [linkage disequilibrium (LD) $r^2 = 1.00$]; rs17652520 was proxied by rs113520245 (LD $r^2 = 0.98$); rs146301099 was proxied by rs957557 (LD $r^2 = 0.83$).

Total household income before tax to SCZ: rs12647647 was proxied by rs12649553 (LD $r^2 = 1.00$); rs146301099 was proxied by rs957557 (LD $r^2 = 0.83$).

Average acceleration to BIP/MDD: rs56194509 was proxied by rs113093579 (LD $r^2 = 1.00$).

Number of cigarettes per day to MDD: rs28813180 was proxied by rs9867617 (LD $r^2 = 1.00$).

Smoking initiation to MDD/SCZ: rs10698713 was proxied by rs10447365 (LD $r^2 = 0.98$).

Long sleep duration to BIP/MDD/SCZ: rs17688916 was proxied by rs62057112 (LD $r^2 = 0.90$).

Sleep duration to BIP/MDD/SCZ: rs1991556 was proxied by rs112310745 (LD $r^2 = 0.98$).

Television watching time to BIP/MDD/SCZ: rs7834121 was proxied by rs7834248 (LD $r^2 = 0.96$); rs111901094 was proxied by rs28720066 (LD $r^2 = 0.98$).

Number of sexual partners to BIP: rs62063281 was proxied by rs112385572 (LD $r^2 = 0.98$); rs6908726 was proxied by rs13208096(LD $r^2 = 0.86$).

Number of sexual partners to MDD/SCZ: rs62063281 was proxied by rs112385572 (LD $r^2 = 0.98$).

Table S8. Statistical power calculation for Mendelian randomization effects derived from random-effects inverse variance weighted method.

| Exposures | BIP | | | MDD | | | SCZ | | |
|---|---|---|---|---|---|---|---|---|---|
| | $R^2$ (%) | *F*-statistics | Power (%) | $R^2$ (%) | *F*-statistics | Power (%) | $R^2$ (%) | *F*-statistics | Power (%) |
| Educational attainment | 1.87 | 28.44-373.78 | 100 | 1.90 | 28.44-373.78 | 100 | 1.77 | 28.44-373.78 | 19 |
| Total household income before tax | 0.26 | 30.10-52.35 | 6 | 0.33 | 30.10-72.87 | 100 | 0.25 | 30.19-72.17 | 100 |
| Relative carbohydrate intake | 0.07 | 30.47-48.73 | 12 | 0.08 | 30.47-48.73 | 87 | 0.08 | 30.47-48.73 | 36 |
| Relative fat intake | 0.10 | 31.70-121.73 | 23 | 0.10 | 31.70-121.73 | 9 | 0.10 | 31.70-121.73 | 51 |
| Relative protein intake | 0.08 | 32.06-59.47 | 50 | 0.14 | 32.06-111.53 | 22 | 0.08 | 32.06-59.47 | 20 |
| Relative sugar intake | 0.06 | 25.00-44.44 | 15 | 0.06 | 25.00-44.44 | 76 | 0.06 | 25.00-44.44 | 15 |
| Average acceleration | 0.26 | 30.12-47.42 | 6 | 0.26 | 30.12-47.42 | 6 | 0.18 | 30.12-36.00 | 5 |
| Moderate to vigorous physical activity | 0.03 | 25.70-31.76 | 92 | 0.03 | 25.70-36.44 | 47 | 0.03 | 25.70-36.44 | 87 |
| Vigorous physical activity | 0.92 | 793.52-814.67 | 10 | 1.48 | 793.52-1461.92 | 14 | 1.17 | 798.17-1461.92 | 62 |
| Age of initiation of regular smoking | 0.08 | 31.80-63.50 | 27 | 0.08 | 31.80-63.50 | 87 | 0.07 | 31.80-63.50 | 7 |
| Number of cigarettes per day | 0.84 | 29.80-1309.99 | 59 | 0.89 | 29.80-1309.99 | 55 | 0.77 | 29.80-1309.99 | 100 |
| Smoking cessation | 0.13 | 30.90-155.00 | 34 | 0.13 | 30.90-155.00 | 34 | 0.11 | 30.90-155.00 | 12 |
| Smoking initiation | 0.74 | 29.80-211.00 | 100 | 0.72 | 29.80-211.00 | 100 | 0.67 | 29.80-211.00 | 100 |
| Alcohol consumption per week | 0.43 | 29.80-1520.00 | 54 | 0.44 | 29.80-1520.00 | 27 | 0.41 | 29.80-1520.00 | 91 |
| Alcohol Use Disorder Identification Test | 0.13 | 49.00-64.00 | 48 | 0.13 | 49.00-64.00 | 100 | 0.13 | 49.00-64.00 | 68 |
| Insomnia | 0.48 | 25.53-216.93 | 26 | 0.48 | 25.53-216.93 | 84 | 0.44 | 25.53-216.93 | 16 |
| Long sleep duration | 0.06 | 15.74-47.84 | 5 | 0.06 | 15.74-47.84 | 6 | 0.06 | 15.74-47.84 | 23 |
| Short sleep duration | 0.02 | 18.09-26.12 | 27 | 0.10 | 16.89-55.73 | 21 | 0.10 | 16.89-55.73 | 15 |
| Sleep duration | 0.50 | 29.63-220.78 | 5 | 0.50 | 29.63-220.78 | 5 | 0.46 | 29.63-220.78 | 5 |
| Computer use time | 0.16 | 34.45-52.06 | 58 | 0.19 | 33.00-79.56 | 38 | 0.18 | 33.00-79.56 | 48 |
| Television watching time | 0.89 | 33.63-144.19 | 31 | 0.94 | 33.63-144.19 | 10 | 0.87 | 33.63-144.19 | 38 |
| Drive time | 0.03 | 34.13-45.01 | 24 | 0.04 | 34.13-45.01 | 16 | 0.03 | 34.13-40.90 | 98 |
| Adventurousness | 0.77 | 29.72-375.97 | 100 | 0.79 | 29.72-375.97 | 9 | 0.73 | 29.72-375.97 | 100 |
| Automobile speeding propensity | 0.23 | 30.82-59.14 | 26 | 0.27 | 29.91-87.18 | 100 | 0.27 | 30.59-87.18 | 100 |
| General risk tolerance | 0.30 | 30.04-177.05 | 100 | 0.31 | 30.04-177.05 | 63 | 0.27 | 30.04-177.05 | 90 |

| | | | | | | | | | |
|---|---|---|---|---|---|---|---|---|---|
| Number of sexual partners | 0.74 | 29.80-79.58 | 100 | 0.75 | 29.80-79.58 | 100 | 0.67 | 29.80-79.58 | 100 |
| Age at first birth | 3.21 | 689.24-1409.50 | 9 | 3.21 | 689.24-1409.50 | 100 | 2.30 | 689.24-935.80 | 24 |
| Number of children ever born | 0.04 | 62.81-64.78 | 6 | 0.04 | 62.81-64.78 | 97 | 0.04 | 62.81-64.78 | 31 |

BIP, bipolar disorder; MDD, major depressive disorder; SCZ, schizophrenia.

$R^2$ presented the proportion of variance in exposure phenotypes explained by single nucleotide polymorphisms (SNPs) using the formula $R^2 = [2 \times BETA^2 \times EAF \times (1 - EAF)]/[2 \times BETA^2 \times EAF \times (1 - EAF) + 2 \times SE(BETA)^2 \times N \times EAF \times (1 - EAF)]$. Here, BETA is the genetic effects on exposures; EAF is effect allele frequency; SE(BETA) is standard error of the genetic effects; N is the sample size. *F*-statistic quantified the strength of each selected SNP using the formula $R^2 \times (N - 2)/(1 - R^2)$.

Table S9. Heterogeneity and pleiotropy assessment for significant results ($p < 0.05$).

| Exposures | Outcomes | Directional pleiotropy | | Cochran's $Q$ test | | | Rucker's $Q'$ test | | $Q - Q'$ | $p$ |
|---|---|---|---|---|---|---|---|---|---|---|
| | | MR-Egger intercept | $p$ | $I^2$ | $Q$ statistic | $p$ | $Q'$ statistic | $p$ | | |
| Educational attainment | BIP | 0.0047 | 0.25 | 46% | 675.0 | 4.76e-21 | 672.5 | 6.23e-21 | 2.5 | 0.11 |
| Smoking cessation | BIP | 0.0031 | 0.87 | 34% | 18.2 | 0.11 | 18.2 | 0.08 | 0 | 0.83 |
| Smoking initiation | BIP | 0.0069 | 0.26 | 46% | 342.5 | 8.80e-12 | 340.0 | 1.14e-11 | 2.5 | 0.19 |
| Insomnia | BIP | -0.0013 | 0.86 | 55% | 319.4 | 3.77e-15 | 319.3 | 2.57e-15 | 0.1 | 0.79 |
| Short sleep duration | BIP | -0.0017 | 0.98 | 23% | 3.89 | 0.27 | 3.89 | 0.14 | 0 | 0.97 |
| Sleep duration | BIP | 0.0156 | 0.16 | 50% | 106.5 | 1.84e-05 | 102.5 | 3.68e-05 | 4.0 | **0.046** |
| Adventurousness | BIP | -0.0007 | 0.93 | 49% | 186.5 | 6.39e-08 | 186.5 | 4.49e-08 | 0 | 0.90 |
| General risk tolerance | BIP | -0.0221 | **0.05** | 39% | 107.4 | 7.42e-04 | 101.1 | 2.16e-03 | 6.3 | **0.01** |
| Number of sexual partners | BIP | 0.0134 | 0.27 | 40% | 112.0 | 4.74e-04 | 109.9 | 5.74e-04 | 2.1 | 0.15 |
| Educational attainment | MDD | -0.0002 | 0.94 | 38% | 588.9 | 1.92e-12 | 588.9 | 1.51e-12 | 0 | 0.92 |
| Total household income before tax | MDD | -0.0088 | 0.61 | 28% | 32.1 | 0.10 | 31.7 | 0.08 | 0.4 | 0.53 |
| Relative carbohydrate intake | MDD | 0.0009 | 0.99 | 16.5% | 5.99 | 0.31 | 5.99 | 0.20 | 0 | 0.98 |
| Relative sugar intake | MDD | -0.0132 | 0.95 | 0% | 2.09 | 0.55 | 2.08 | 0.35 | 0.1 | 0.95 |
| Age of initiation of regular smoke | MDD | -0.0381 | 0.12 | 32% | 8.87 | 0.18 | 5.18 | 0.39 | 3.69 | 0.055 |
| Smoking cessation | MDD | 0.0070 | 0.54 | 23% | 15.5 | 0.21 | 15.0 | 0.18 | 0.5 | 0.46 |
| Smoking initiation | MDD | 0.0104 | **0.004** | 35% | 281.1 | 3.33e-06 | 268.7 | 2.49e-05 | 12.4 | **4.27E-04** |
| Insomnia | MDD | 0.0069 | 0.08 | 34% | 221.8 | 5.24e-05 | 217.1 | 9.87e-05 | 4.7 | **0.03** |
| Short sleep duration | MDD | 0.0200 | 0.22 | 49% | 31.1 | 0.01 | 28.0 | 0.02 | 3.1 | 0.08 |
| Automobile speeding propensity | MDD | 0.0146 | 0.21 | 43% | 47.2 | 0.01 | 44.5 | 0.01 | 2.7 | 0.09 |
| Number of sexual partners | MDD | 0.0034 | 0.68 | 45% | 122.7 | 5.40e-05 | 122.4 | 4.2e-05 | 0.3 | 0.58 |
| Age at first birth | MDD | 0.0411 | 0.36 | 43% | 14.1 | 0.08 | 12.4 | 0.09 | 1.7 | 0.19 |
| Total household income before tax | SCZ | -0.0034 | 0.93 | 65% | 51.1 | 5.11e-05 | 51.1 | 2.85e-05 | 0 | 0.88 |
| Moderate to vigorous physical activity | SCZ | 0.0325 | 0.78 | 0% | 0.5 | 0.92 | 0.4 | 0.82 | 0.1 | 0.75 |
| Number of cigarettes per day | SCZ | -0.0003 | 0.96 | 57% | 60.2 | 1.60e-04 | 60.1 | 9.99e-05 | 0.1 | 0.94 |

| | | | | | | | | | | |
|---|---|---|---|---|---|---|---|---|---|---|
| Smoking initiation | SCZ | 0.0033 | 0.57 | 57% | 401.0 | 3.82e-20 | 400.2 | 3.10e-20 | 0.8 | 0.38 |
| Alcohol consumption per week | SCZ | 0.0022 | 0.52 | 46% | 101.2 | 1.50e-04 | 100.4 | 1.30e-04 | 0.8 | 0.38 |
| Insomnia | SCZ | 0.0167 | **0.01** | 61% | 346.9 | 8.55e-21 | 329.4 | 1.15e-18 | 17.5 | **2.89E-05** |
| Long sleep duration | SCZ | 0.0034 | 0.88 | 40% | 10.0 | 0.12 | 10.0 | 0.08 | 0 | 0.83 |
| Sleep duration | SCZ | -0.0038 | 0.71 | 61% | 126.2 | 9.77e-09 | 125.8 | 6.68e-09 | 0.4 | 0.54 |
| Adventurousness | SCZ | 0.0080 | 0.31 | 60% | 220.3 | 4.00e-13 | 217.7 | 5.61e-13 | 2.6 | 0.10 |
| Automobile speeding propensity | SCZ | 0.0179 | 0.28 | 57% | 59.9 | 1.71e-04 | 57.2 | 2.52e-04 | 2.7 | 0.10 |
| Number of sexual partners | SCZ | -0.0064 | 0.59 | 52% | 126.6 | 1.69e-06 | 126.0 | 1.35e-06 | 0.6 | 0.43 |

BIP, bipolar disorder; MDD, major depressive disorder; SCZ, schizophrenia.

Table S10. Confirmed confounders for mental disorders (BIP, MDD and SCZ) from previous Mendelian randomization studies.

| Outcomes | Confounders |
|---|---|
| BIP | Except for adventurousness, insomnia, smoking initiation and educational attainment identified in the present study, following factors have been confirmed:<br>C-Reactive Protein (PMID: 27327646)<br>Magnesium, copper (PMID: 27327646) |
| MDD | Except for age at first birth, automobile speeding propensity, number of sexual partners and educational attainment identified in the present study, following factors have been confirmed:<br>Trunk fat percentage, Arm fat percentage (right), Arm fat percentage (left), Leg fat percentage (right), Leg fat percentage (left), Trunk fat mass, Arm fat mass (right), Arm fat mass (left), Leg fat mass (right), Leg fat mass (left), body mass index (BMI), weight, Body fat percentage, Body fat mass (PMID: 31383844)<br>statin and PCSK9 inhibitor therapy (PMID: 29986042)<br>neuroticism (PMID: 30697695)<br>Bacilli (PMID: 33008395)<br>intra-cellular volume fraction (PMID: 32385265)<br>serum morning plasma cortisol level (PMID: 31474942)<br>triglycerides, interleukin-6 (IL-6), and C-reactive protein (CRP) (PMID: 30886334)<br>ADHD (PMID: 32249726)<br>confiding in others, daytime napping (PMID: 32791893)<br>testosterone (PMID: 32610558)<br>Aminoacyl-tRNA biosynthesis (PMID: 31919502) |
| SCZ | Except for automobile speeding propensity, number of sexual partners, cigarettes per day and smoking initiation identified in the present study, following factor have been confirmed:<br>Cannabis use (PMID: 28115737)<br>C-reactive protein, sIL-6R (PMID: 29094161)<br>fasting insulin levels (PMID: 30100396)<br>neuroticism (PMID: 32578352)<br>plasma total homocysteine (PMID: 26208850) |

| | |
|---|---|
| | plays computer games (PMID: 31766499)<br><br>2-methoxyacetaminophen sulfate, glycine, serine, threonine metabolism (PMID: 31919502)<br><br>Enterobacteriaceae family, Enterobacteriales order, Gammaproteobacteria class (PMID: 33008395) |

BIP, bipolar disorders; MDD, major depressive disorders; SCZ, schizophrenia; PMID: PubMed identifier.

Table S11. SNPs associated with confirmed confounders at genome-wide significance ($p < 5 \times 10^{-8}$).

| Phenotype in the present study | Outcome | SNP | Confounders |
|---|---|---|---|
| Adventurousness | BIP | rs10789436 | Years of educational attainment |
| Adventurousness | BIP | rs12023775 | Years of educational attainment |
| Adventurousness | BIP | rs12534625 | Years of educational attainment |
| Adventurousness | BIP | rs734073 | Serum magnesium |
| insomnia | BIP | rs11090039 | Neuroticism |
| insomnia | BIP | rs13010288 | Years of educational attainment |
| insomnia | BIP | rs13135092 | Adventurousness |
| insomnia | BIP | rs1620977 | Years of educational attainment |
| insomnia | BIP | rs28582096 | Years of educational attainment |
| insomnia | BIP | rs34967082 | Years of educational attainment |
| insomnia | BIP | rs3774751 | Years of educational attainment |
| insomnia | BIP | rs6119267 | Years of educational attainment |
| Smoking initiation | BIP | rs2526390 | Years of educational attainment |
| Smoking initiation | BIP | rs2710634 | Years of educational attainment |
| Smoking initiation | BIP | rs329124 | Years of educational attainment |
| Smoking initiation | BIP | rs76608582 | Years of educational attainment |
| Smoking initiation | BIP | rs951740 | Years of educational attainment |
| Number of sexual partners | MDD | rs10786721 | Weight |
| Number of sexual partners | MDD | rs12042107 | Years of educational attainment |
| Number of sexual partners | MDD | rs13093086 | Drive faster than motorway speed limit |
| Number of sexual partners | MDD | rs17149632 | Neuroticism |
| Number of sexual partners | MDD | rs2163971 | Drive faster than motorway speed limit; Weight |
| Number of sexual partners | MDD | rs2422136 | Body Mass Index, Leg fat mass left, Leg fat mass right, Leg fat percentage right, Leg fat percentage left, Weight, Arm fat mass left, Whole body fat mass, Arm fat mass right |
| Number of sexual partners | MDD | rs273512 | Body fat percentage, Trunk fat percentage, Leg fat percentage right, |

| | | | |
|---|---|---|---|
| | | | Trunk fat mass, Leg fat mass right, Arm fat percentage left, Whole body fat mass, Leg fat percentage left, Leg fat mass left, Arm fat percentage right, Arm fat mass left, Arm fat mass right, Body mass index, Weight |
| Number of sexual partners | MDD | rs539096 | Years of educational attainment |
| Number of sexual partners | MDD | rs62063281 | Neuroticism |
| Number of sexual partners | MDD | rs6504568 | Trunk fat mass; Trunk fat percentage; Body fat percentage; Whole body fat mass; Arm fat percentage left; Leg fat mass left; Leg fat mass right; Arm fat mass left; Weight; Arm fat percentage right; Arm fat mass right; Leg fat percentage right; Leg fat percentage left; Body mass index |
| Number of sexual partners | MDD | rs7942078 | Body mass index |
| Number of sexual partners | MDD | rs9922596 | Weight; Arm fat mass left; Arm predicted mass left; Whole body fat mass; Arm fat mass right; Trunk fat mass |
| Educational attainment | MDD | rs10761251 | Leg fat percentage right; Leg fat percentage left; Leg fat mass right; Leg fat mass left |
| Educational attainment | MDD | rs10773208 | Weight; Trunk fat mass; Whole body fat mass; Arm fat mass right; Arm fat mass left; Leg fat mass left; Leg fat mass right; Trunk fat percentage; Body fat percentage; Arm fat percentage left; Arm fat percentage right; Body mass index |
| Educational attainment | MDD | rs10798888 | Leg fat mass right; Leg fat mass left; Whole body fat mass; Leg fat percentage right; Body fat percentage; Trunk fat mass; Leg fat percentage left; Arm fat mass right; Arm fat mass left; Trunk fat percentage; Arm fat percentage left; Weight; Arm fat percentage right; Body mass index |
| Educational attainment | MDD | rs10805383 | Arm fat percentage left; Arm fat mass left; Arm fat percentage right; Body mass index; Arm fat mass right; Leg fat mass right; Leg fat mass left; Leg fat percentage left; Leg fat percentage right; Body fat |

| | | | |
|---|---|---|---|
| | | | percentage; Trunk fat mass; Whole body fat mass; Trunk fat percentage |
| Educational attainment | MDD | rs10875121 | Arm fat percentage left; Arm fat mass left; Arm fat percentage right; Body mass index; Arm fat mass right; Leg fat mass right; Leg fat mass left; Leg fat percentage left; Leg fat percentage right; Body fat percentage; Trunk fat mass; Whole body fat mass; Trunk fat percentage |
| Educational attainment | MDD | rs11030102 | Arm fat percentage left; Arm fat mass left; Arm fat percentage right; Body mass index; Arm fat mass right; Leg fat mass right; Leg fat mass left; Leg fat percentage left; Leg fat percentage right; Body fat percentage; Trunk fat mass; Whole body fat mass; Trunk fat percentage |
| Educational attainment | MDD | rs11082011 | Neuroticism |
| Educational attainment | MDD | rs1167827 | Body mass index; Arm fat mass right; Arm fat percentage left; Arm fat mass left; Arm fat percentage right; Leg fat mass right; Leg fat mass left; Weight; Body fat percentage; Leg fat percentage left; Leg fat percentage right<br>Trunk fat mass; Trunk fat percentage |
| Educational attainment | MDD | rs12375949 | Leg fat percentage right; Leg fat percentage left; Leg fat mass right; Body fat percentage; Leg fat mass left |
| Educational attainment | MDD | rs12981405 | Leg fat percentage left; Leg fat percentage right |
| Educational attainment | MDD | rs13018640 | Body mass index; Arm fat mass right; Arm fat percentage left; Arm fat mass left; Arm fat percentage right; Leg fat mass right; Leg fat mass left; Weight; Body fat percentage; Leg fat percentage left; Leg fat percentage right; Trunk fat mass; Trunk fat percentage |
| Educational attainment | MDD | rs1334297 | Body mass index; Arm fat mass right; Arm fat percentage left; Arm fat mass left; Arm fat percentage right; Leg fat mass right; Leg fat mass left; Body fat percentage; Leg fat percentage left; Leg fat percentage right; Whole body fat mass |
| Educational attainment | MDD | rs1564347 | Leg fat percentage right; Leg fat percentage left |
| Educational attainment | MDD | rs1689510 | Weight; Body mass index; Arm fat mass right |

| | | | |
|---|---|---|---|
| Educational attainment | MDD | rs17411339 | Neuroticism |
| Educational attainment | MDD | rs2034631 | Trunk fat mass |
| Educational attainment | MDD | rs2183271 | Body mass index; Arm fat mass right; Arm fat percentage left; Arm fat mass left; Arm fat percentage right; Leg fat mass right; Leg fat mass left; Body fat percentage; Leg fat percentage left; Leg fat percentage right; Whole body fat mass |
| Educational attainment | MDD | rs2725370 | Leg fat percentage left; Leg fat mass left; Leg fat percentage right; Body mass index; Leg fat mass right; Whole body fat mass; Body fat percentage; Trunk fat mass; Arm fat mass right; Weight; Trunk fat percentage; Arm fat percentage left; Arm fat percentage right |
| Educational attainment | MDD | rs2838006 | Body mass index |
| Educational attainment | MDD | rs303752 | Weight; Trunk fat mass; Whole body fat mass; Leg fat mass left; Leg fat mass right; Arm fat mass right; Body fat percentage; Trunk fat percentage; Arm fat mass left |
| Educational attainment | MDD | rs34720381 | Body Mass Index |
| Educational attainment | MDD | rs3809634 | Weight |
| Educational attainment | MDD | rs3948495 | Leg fat mass left; Leg fat mass right; Leg fat percentage left; Leg fat percentage right |
| Educational attainment | MDD | rs42302 | Weight; Body mass index; Leg fat mass right; Leg fat mass left; Arm fat mass right; Arm fat mass left; Whole body fat mass; Trunk fat mass; Arm fat percentage left; Body fat percentage; Leg fat percentage left; Leg fat percentage right; Arm fat percentage right; Trunk fat percentage |
| Educational attainment | MDD | rs4500930 | Weight; Body mass index; Leg fat mass right; Leg fat mass left; Arm fat mass right; Arm fat mass left; Whole body fat mass; Trunk fat mass; Arm fat percentage left; Body fat percentage; Leg fat percentage left; Leg fat percentage right; Arm fat percentage right; Trunk fat percentage |

| | | | |
|---|---|---|---|
| Educational attainment | MDD | rs4787457 | Weight; Body mass index; Leg fat mass right; Leg fat mass left; Arm fat mass right; Arm fat mass left; Whole body fat mass; Trunk fat mass; Arm fat percentage left; Body fat percentage; Leg fat percentage left; Leg fat percentage right; Arm fat percentage right; Trunk fat percentage |
| Educational attainment | MDD | rs4899012 | Weight; Trunk fat mass |
| Educational attainment | MDD | rs56319902 | Neuroticism |
| Educational attainment | MDD | rs56391344 | Weight; Body mass index; Leg fat mass right; Leg fat mass left; Arm fat mass right; Arm fat mass left; Whole body fat mass; Trunk fat mass; Body fat percentage; Leg fat percentage left; Leg fat percentage right; Arm fat percentage right |
| Educational attainment | MDD | rs62155873 | Body mass index |
| Educational attainment | MDD | rs66568921 | Weight; Body mass index; Leg fat mass right; Leg fat mass left; Arm fat mass right; Arm fat mass left; Whole body fat mass; Trunk fat mass; Arm fat percentage left; Body fat percentage; Leg fat percentage left; Leg fat percentage right; Arm fat percentage right |
| Educational attainment | MDD | rs72828517 | Drive faster than motorway speed limit |
| Educational attainment | MDD | rs746839 | Leg fat mass left; Leg fat mass right; Weight; Whole body fat mass; Trunk fat mass; Arm fat mass left; Body mass index |
| Educational attainment | BIP | rs75177132 | Smoking initiation |
| Educational attainment | MDD | rs7650602 | Weight; Trunk fat mass; Leg fat mass right; Leg fat mass left; Arm fat mass right; Arm fat mass left; Trunk fat percentage |
| Educational attainment | MDD | rs7692359 | Trunk fat percentage; Body fat percentage |
| Educational attainment | MDD | rs78648104 | Weight; Body mass index; Leg fat mass right; Leg fat mass left; Arm fat mass right; Arm fat mass left; Whole body fat mass; Trunk fat mass; Arm fat percentage left; Arm fat percentage right |
| Educational attainment | MDD | rs7924036 | Body mass index |
| Educational attainment | MDD | rs7974852 | Leg fat percentage right |

| | | | |
|---|---|---|---|
| Educational attainment | MDD | rs8024 | Weight; Body mass index; Leg fat mass right; Leg fat mass left; Arm fat mass right; Arm fat mass left; Whole body fat mass; Trunk fat mass; Arm fat percentage left; Body fat percentage; Leg fat percentage left; Leg fat percentage right; Arm fat percentage right |
| Educational attainment | MDD | rs9375188 | Leg fat percentage right; Body fat percentage; Trunk fat percentage; Leg fat percentage left; Arm fat percentage left; Leg fat mass right; Leg fat mass left; Whole body fat mass; Arm fat percentage right; Trunk fat mass; Arm fat mass left; Arm fat mass right; Body mass index |
| Educational attainment | MDD | rs9859556 | Leg fat percentage right; Body fat percentage; Trunk fat percentage; Leg fat percentage left; Arm fat percentage left; Leg fat mass right; Leg fat mass left; Whole body fat mass; Arm fat percentage right; Trunk fat mass; Arm fat mass left; Arm fat mass right; Body mass index |
| Educational attainment | MDD | rs9927137 | Leg fat percentage right; Body fat percentage; Trunk fat percentage; Leg fat percentage left; Arm fat percentage left; Leg fat mass right; Leg fat mass left; Whole body fat mass; Arm fat percentage right; Trunk fat mass; Arm fat mass left; Arm fat mass right; Body mass index |
| Age at first birth | MDD | rs10056247 | Body mass index |
| Age at first birth | MDD | rs1160544 | Years of educational attainment |
| Age at first birth | MDD | rs2777888 | Years of educational attainment; Leg fat percentage right; Body fat percentage; Trunk fat percentage; Leg fat percentage left; Arm fat percentage left; Leg fat mass right; Leg fat mass left; Whole body fat mass; Arm fat percentage right; Trunk fat mass; Arm fat mass left; Arm fat mass right; Body mass index; Weight |
| Automobile speeding propensity | MDD | rs10465231 | Arm fat percentage right; Body fat percentage; Arm fat percentage left; Body mass index; Leg fat percentage right; Leg fat percentage left; |

| | | | |
|---|---|---|---|
| | | | Trunk fat percentage; Whole body fat mass; Arm fat mass left; Arm fat mass right; Leg fat mass right; Leg fat mass left; Trunk fat mass |
| Automobile speeding propensity | MDD | rs10858922 | Body fat percentage; Body mass index; Leg fat percentage right; Leg fat percentage left; Trunk fat percentage; Whole body fat mass; Arm fat mass left; Arm fat mass right; Leg fat mass right; Leg fat mass left; Trunk fat mass |
| Automobile speeding propensity | MDD | rs12325727 | Years of educational attainment |
| Automobile speeding propensity | MDD | rs13083798 | Body mass index |
| Automobile speeding propensity | MDD | rs17516256 | Weight; Body mass index; Leg fat mass left; Leg fat mass right; Arm fat mass left; Arm fat mass right |
| Automobile speeding propensity | MDD | rs185819 | Weight; Neuroticism |
| Automobile speeding propensity | MDD | rs362307 | Years of educational attainment; Arm fat percentage right; Arm fat percentage left; Body mass index; Leg fat percentage right; Leg fat percentage left; Arm fat mass left; Arm fat mass right; Leg fat mass right; Leg fat mass left |
| Automobile speeding propensity | MDD | rs619466 | Neuroticism |
| Automobile speeding propensity | MDD | rs2409691 | Neuroticism; Body mass index; Leg fat mass right; Arm fat mass right; Leg fat mass left; Arm fat mass left |
| Number of sexual partners | SCZ | rs13093086 | Drive faster than motorway speed limit |
| Number of sexual partners | SCZ | rs17149632 | Neuroticism |
| Number of sexual partners | SCZ | rs17785382 | Schizophrenia |
| Number of sexual partners | SCZ | rs2163971 | Drive faster than motorway speed limit |
| Number of sexual partners | SCZ | rs35219418 | Smoking initiation |
| Number of sexual partners | SCZ | rs62063281 | Neuroticism |
| Smoking initiation | SCZ | rs1549979 | Drive faster than motorway speed limit |
| Smoking initiation | SCZ | rs2155646 | Neuroticism |
| Smoking initiation | SCZ | rs62007780 | Neuroticism |
| Automobile speeding propensity | SCZ | rs185819 | Neuroticism |

| | | | |
|---|---|---|---|
| Automobile speeding propensity | SCZ | rs2409691 | Neuroticism |
| Automobile speeding propensity | SCZ | rs619466 | Neuroticism |

SNP, single nucleotide polymorphisms; BIP, bipolar disorder; MDD, major depressive disorder; SCZ, schizophrenia

Table S12. Inverse variance weighted (IVW) analysis after exclusion of SNPs associated with potential confounders.

| Exposures | Outcomes | IVW analysis | | |
|---|---|---|---|---|
| | | OR | 95% CI | *p* values |
| Smoking initiation | BIP | 1.59 | 1.37-1.86 | 2.34E-09 |
| Insomnia | BIP | 1.17 | 1.08-1.28 | 2.53E-04 |
| Adventurousness | BIP | 1.93 | 1.44-2.59 | 1.01E-05 |
| Educational attainment | MDD | 0.73 | 0.65-0.82 | 1.80E-07 |
| Automobile speeding propensity | MDD | 0.56 | 0.38-0.82 | 2.78E-03 |
| Number of sexual partners | MDD | 1.39 | 1.13-1.71 | 1.49E-03 |
| Age at first birth | MDD | 0.83 | 0.72-0.96 | 1.02E-02 |
| Smoking initiation | SCZ | 1.64 | 1.42-1.88 | 8.20E-12 |
| Automobile speeding propensity | SCZ | 0.42 | 0.28-0.65 | 1.04E-04 |
| Number of sexual partners | SCZ | 2.33 | 1.79-3.04 | 4.28E-10 |

SNP, single nucleotide polymorphisms; BIP, bipolar disorder; MDD, major depressive disorder; SCZ, schizophrenia; OR, odds ratio; CI, confidence interval.

**Figure S1.** Scatter plots showing robust genetic associations of socioeconomic status/individual behaviors with mental disorders.

Each of the SNPs associated with exposures is represented by a black dot with the error bar depicting the standard error of its association with a specific exposure (horizontal) and the target mental disorder (vertical). The slopes of each line represent the causal association for each Mendelian randomization method.

BIP, bipolar disorder; MDD, major depression; SCZ, schizophrenia.

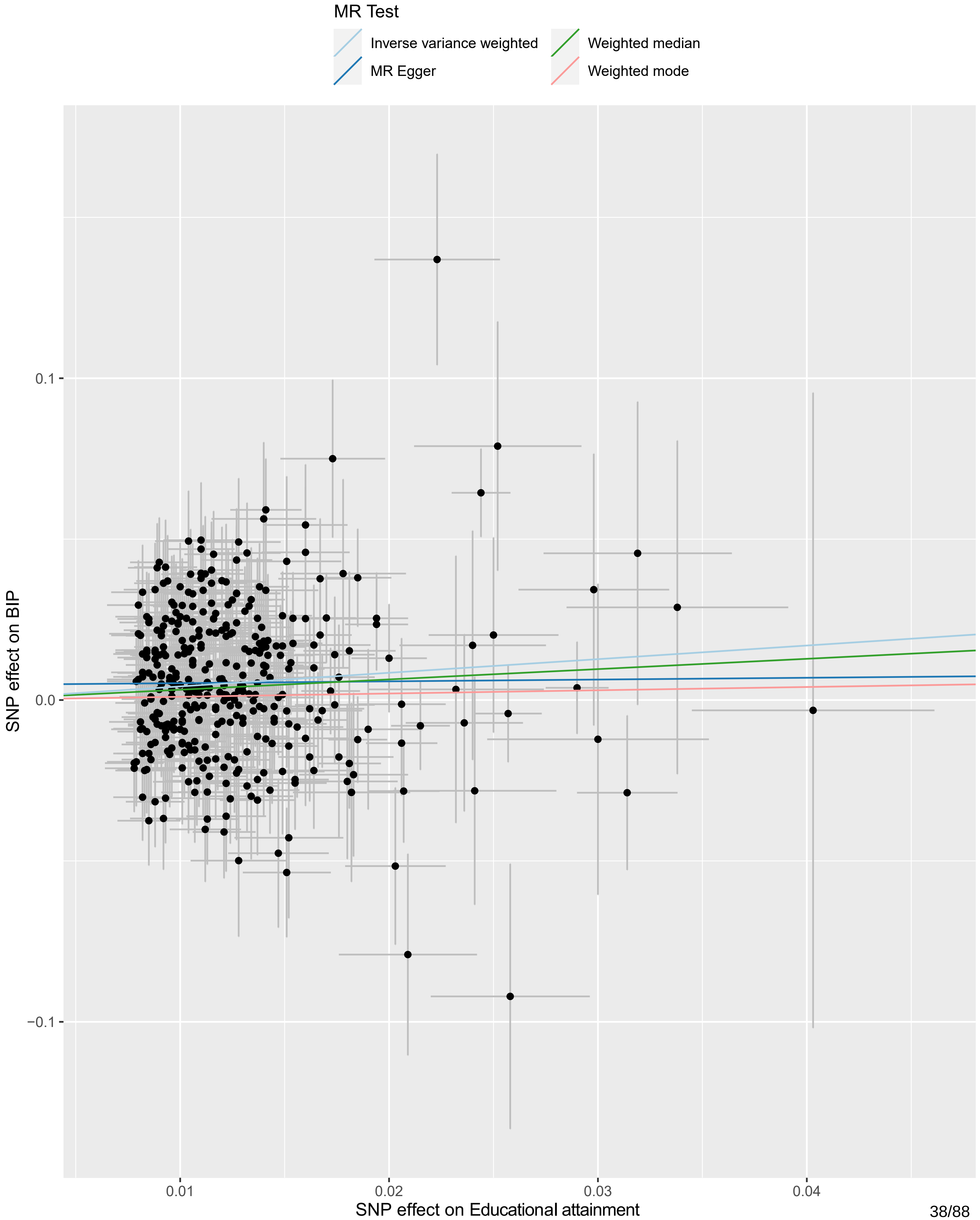

MR Test
Inverse variance weighted
MR Egger
Weighted median
Weighted mode
0.1
0.0
−0.1
0.01
0.02
0.03
0.04
SNP effect on BIP
SNP effect on Educational attainment

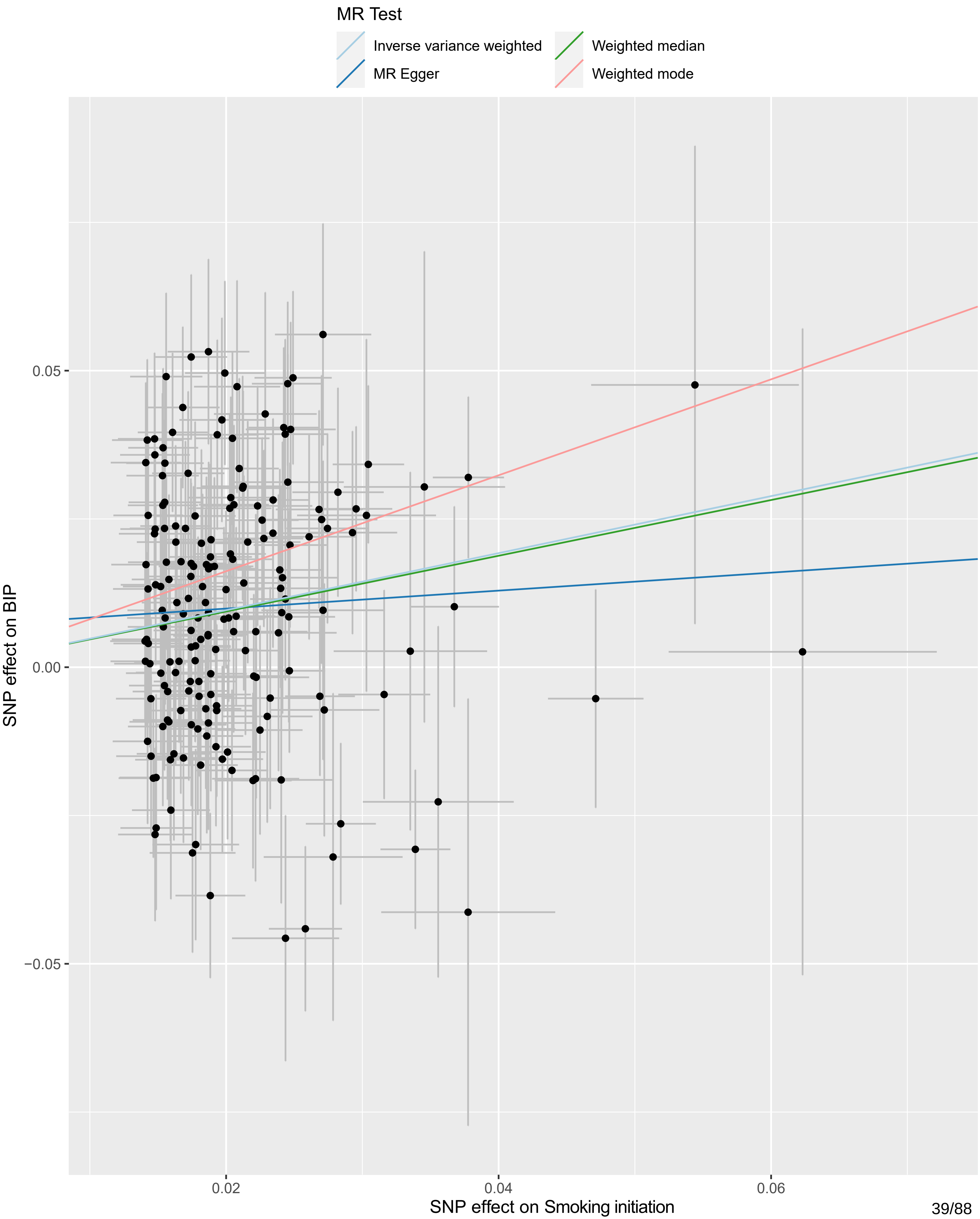

MR Test
Inverse variance weighted
MR Egger
Weighted median
Weighted mode
SNP effect on BIP
0.05
0.00
−0.05
0.02
0.04
0.06
SNP effect on Smoking initiation

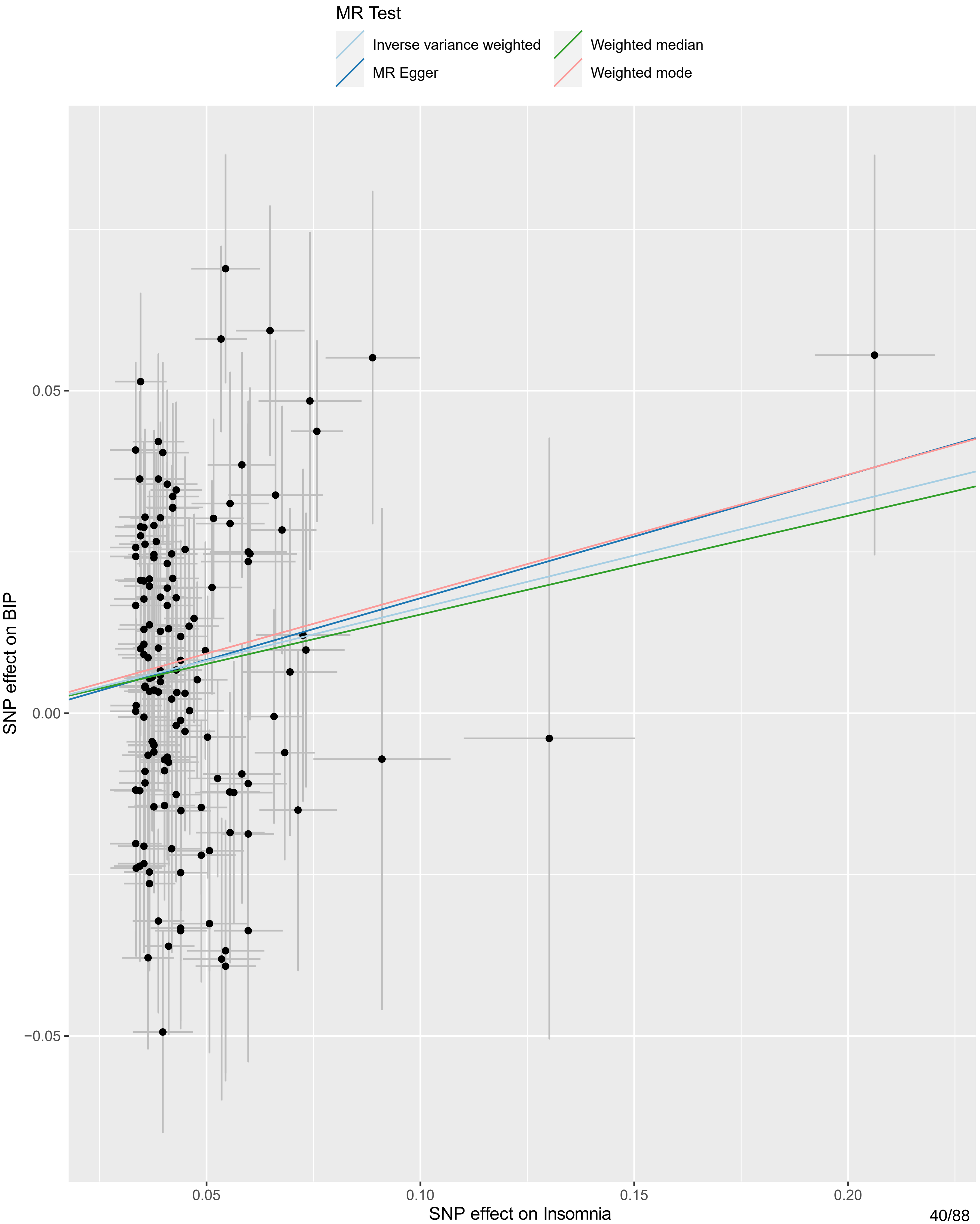

MR Test
Inverse variance weighted
MR Egger
Weighted median
Weighted mode
SNP effect on BIP
0.05
0.00
−0.05
0.05
0.10
0.15
0.20
SNP effect on Insomnia

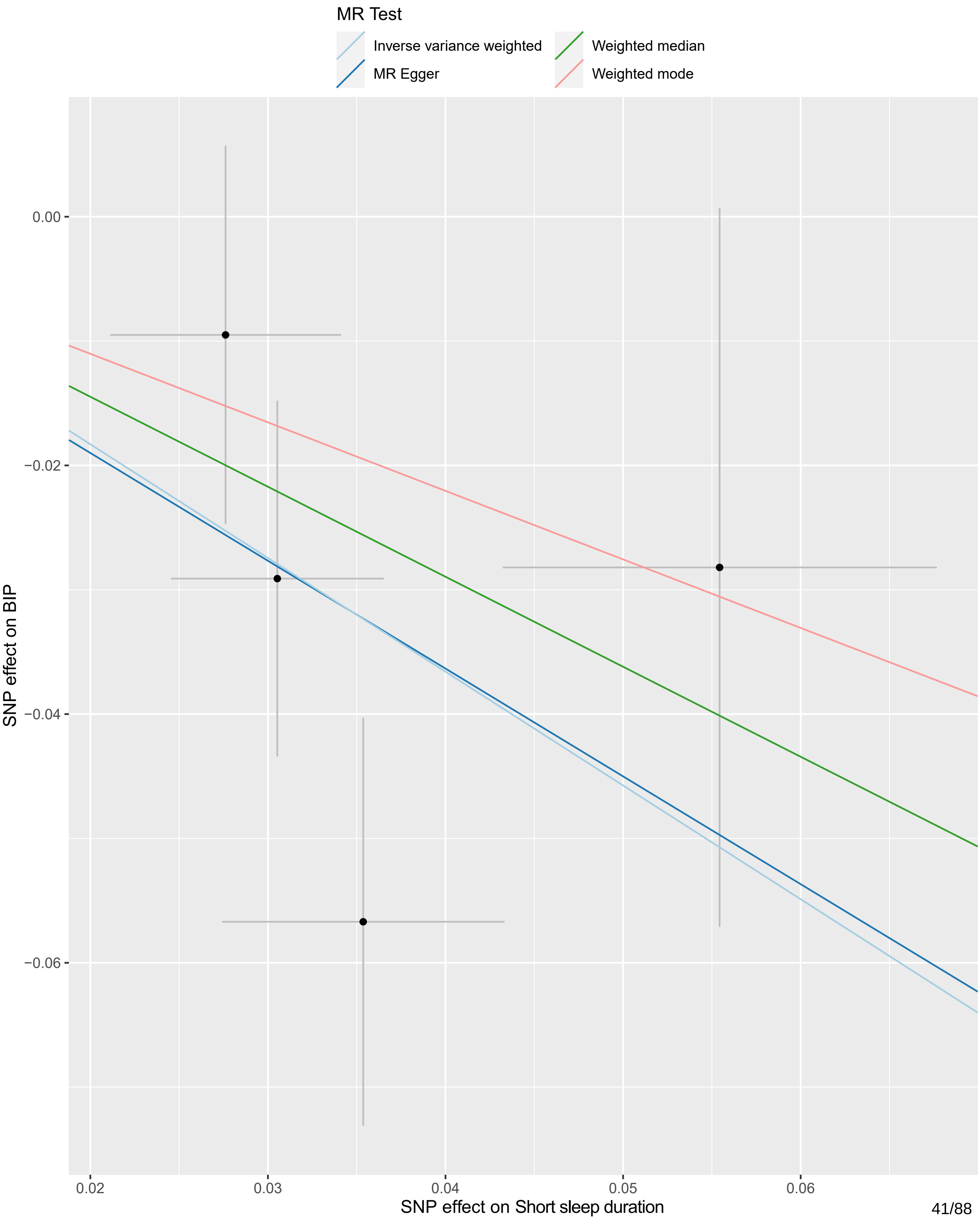
MR Test
Inverse variance weighted
MR Egger
Weighted median
Weighted mode
0.00
−0.02
−0.04
−0.06
SNP effect on BIP
0.02
0.03
0.04
0.05
0.06
SNP effect on Short sleep duration

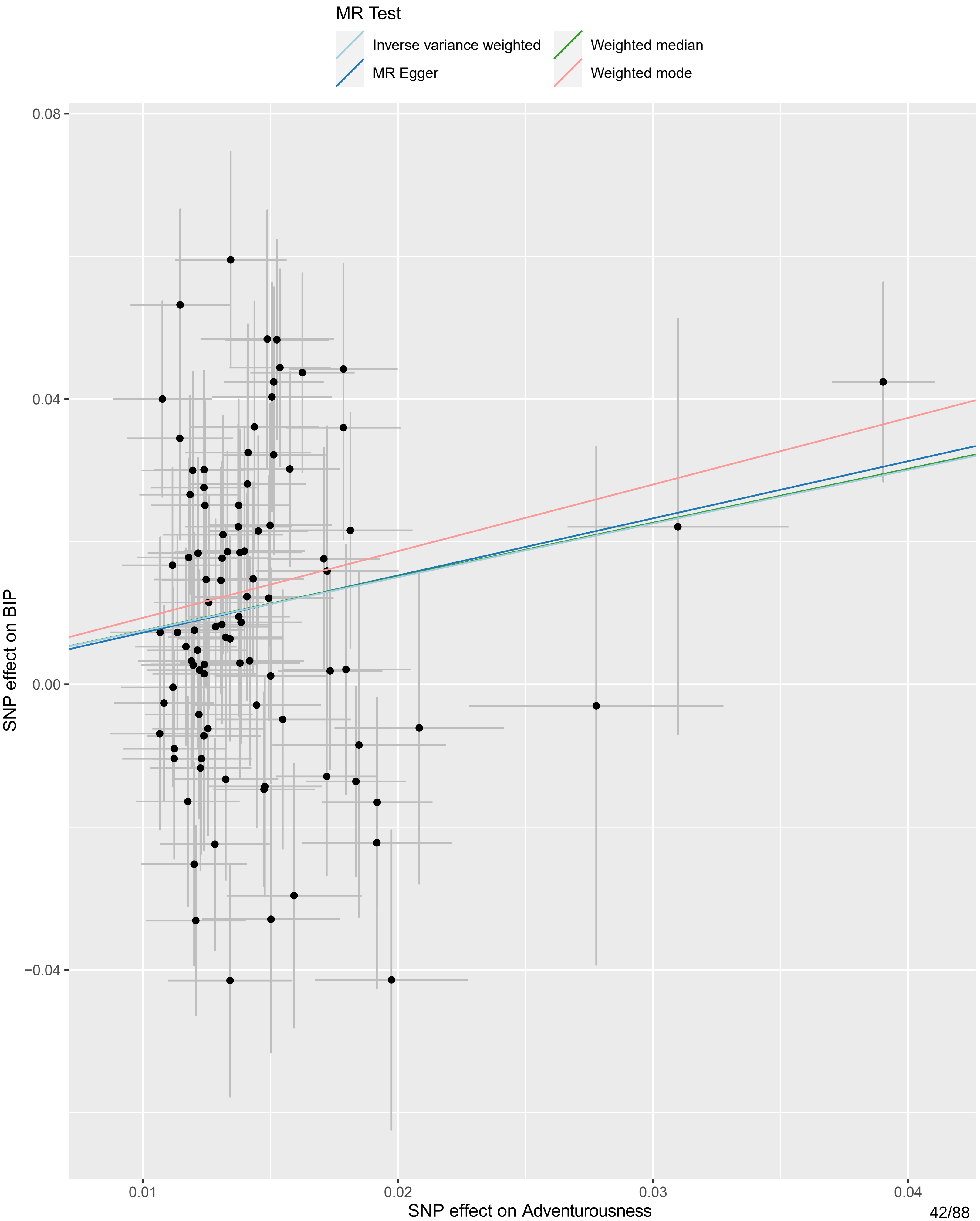
MR Test
Inverse variance weighted
MR Egger
Weighted median
Weighted mode
0.08
0.04
0.00
−0.04
SNP effect on BIP
0.01
0.02
0.03
0.04
SNP effect on Adventurousness

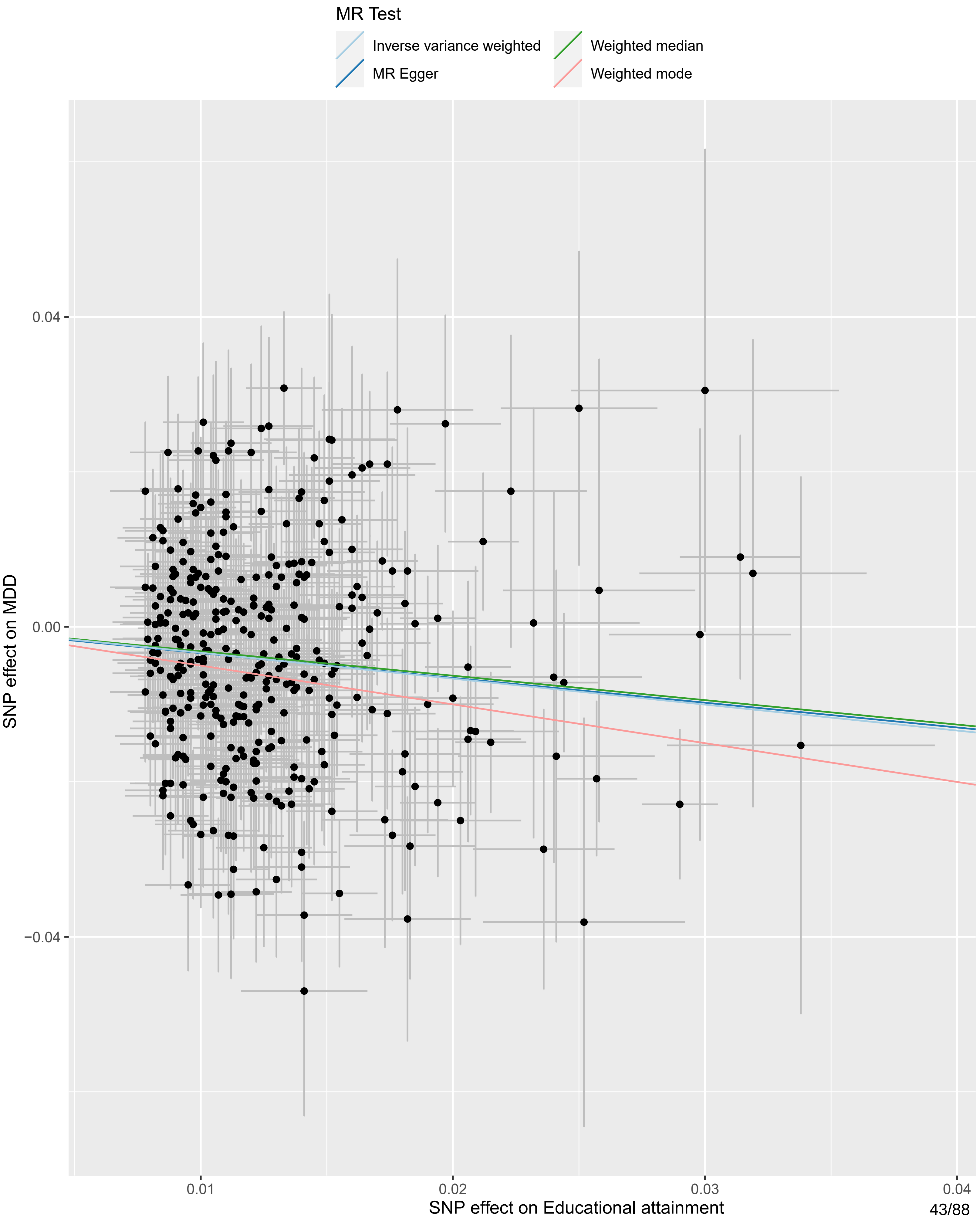

MR Test
Inverse variance weighted
MR Egger
Weighted median
Weighted mode
SNP effect on MDD
0.04
0.00
−0.04
0.01
0.02
0.03
0.04
SNP effect on Educational attainment

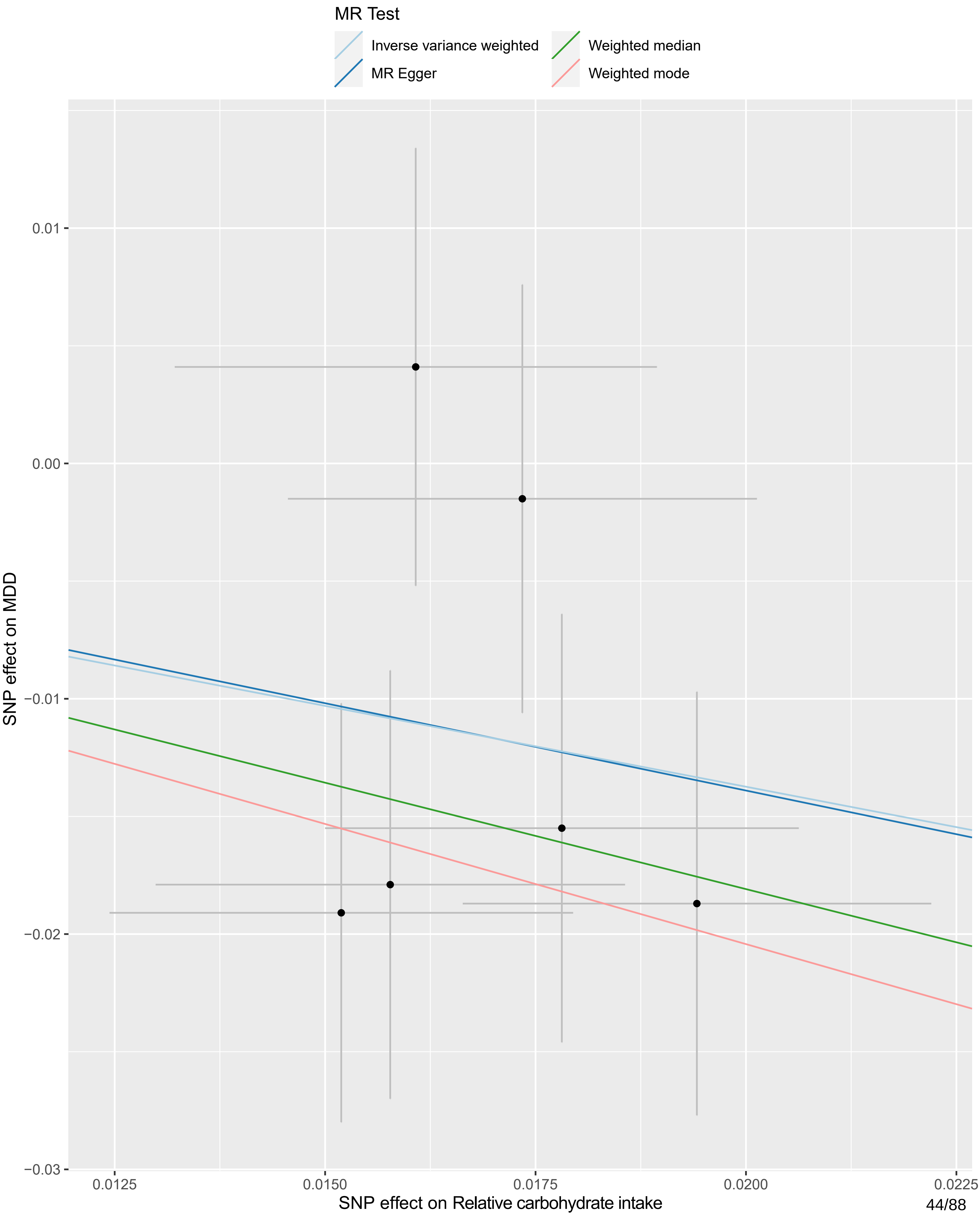
MR Test
Inverse variance weighted
MR Egger
Weighted median
Weighted mode
SNP effect on MDD
0.01
0.00
−0.01
−0.02
−0.03
0.0125
0.0150
0.0175
0.0200
0.0225
SNP effect on Relative carbohydrate intake

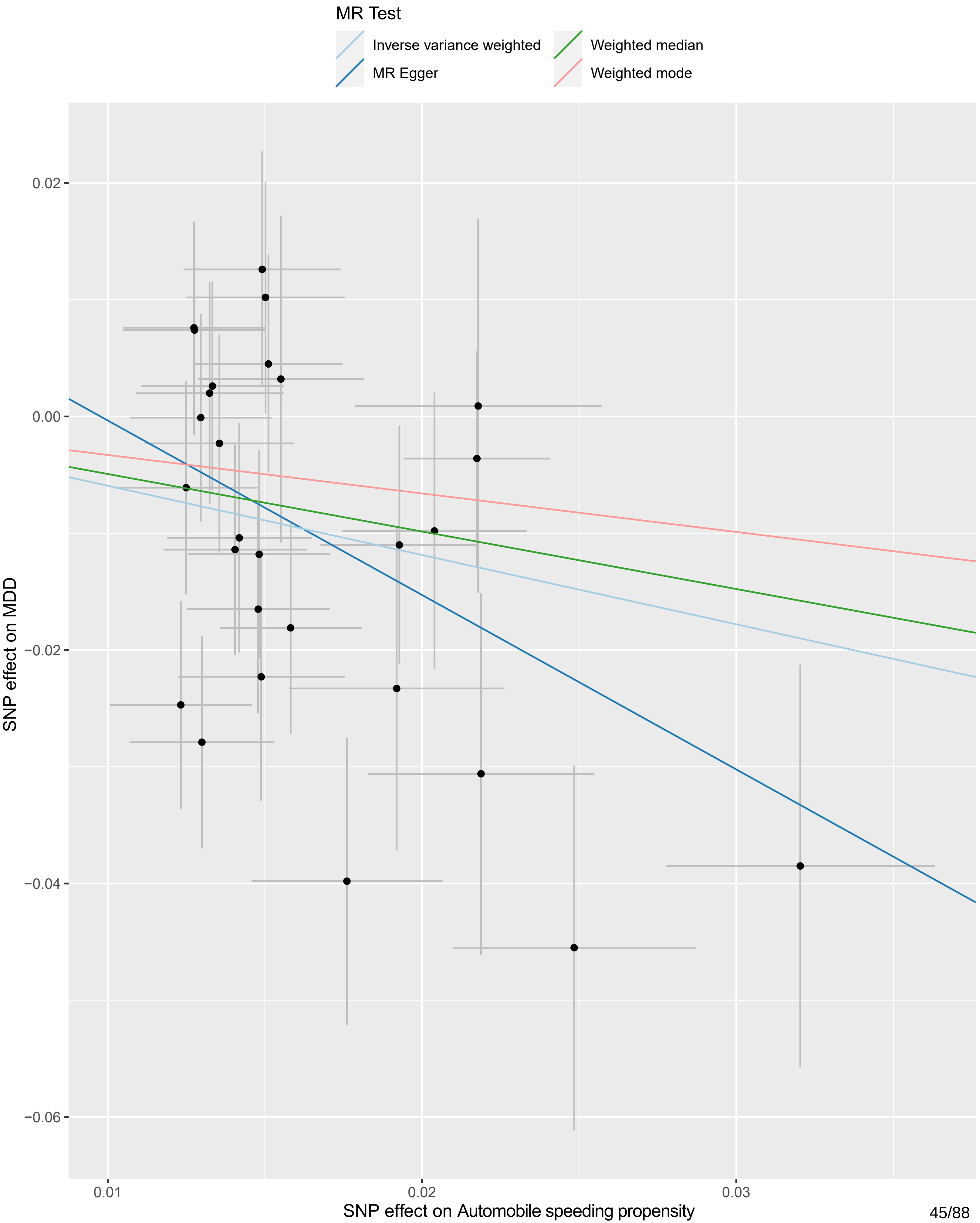

MR Test
Inverse variance weighted
MR Egger
Weighted median
Weighted mode
0.02
0.00
−0.02
−0.04
−0.06
SNP effect on MDD
0.01
0.02
0.03
SNP effect on Automobile speeding propensity

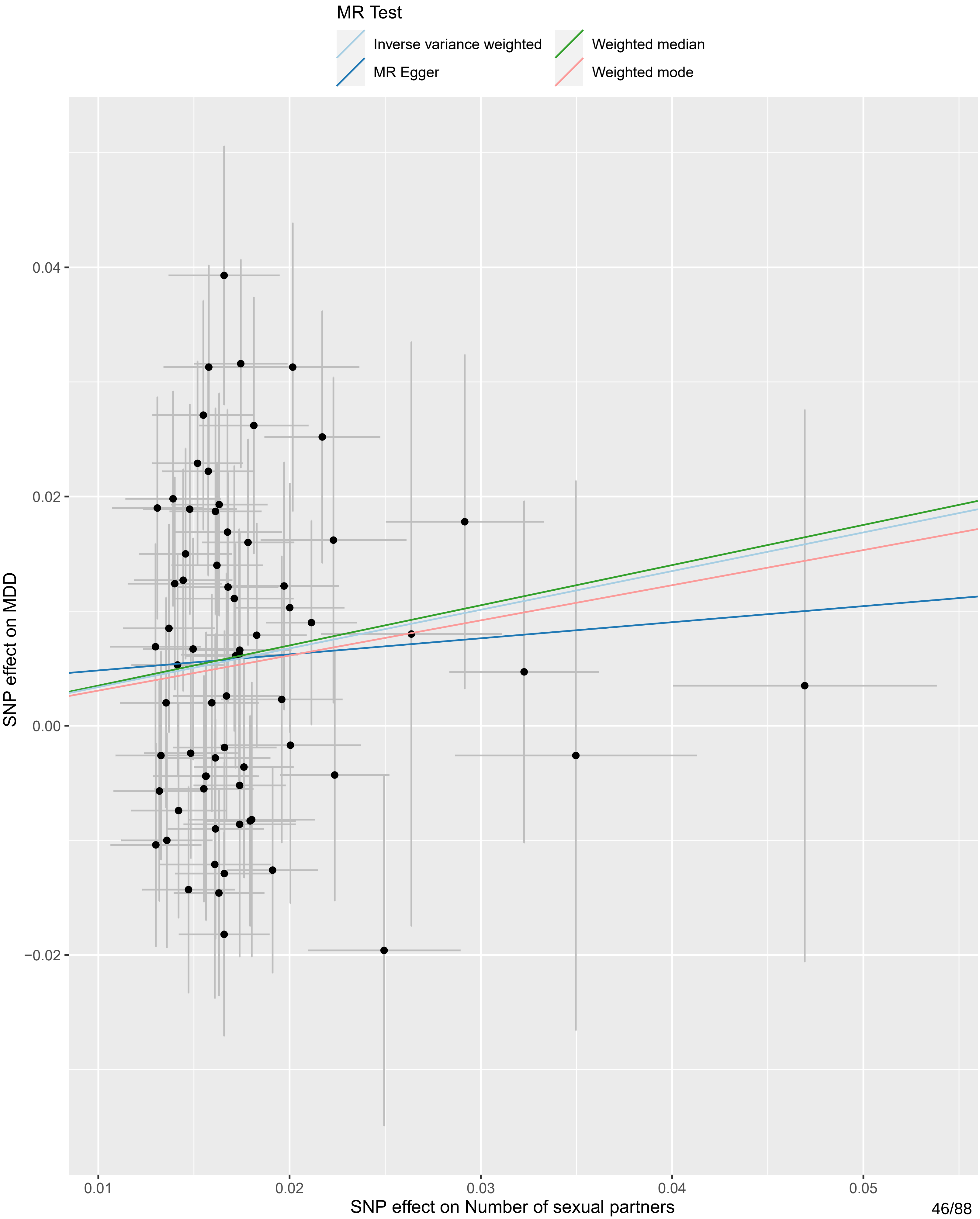
MR Test
Inverse variance weighted
MR Egger
Weighted median
Weighted mode
0.04
0.02
0.00
−0.02
SNP effect on MDD
0.01
0.02
0.03
0.04
0.05
SNP effect on Number of sexual partners

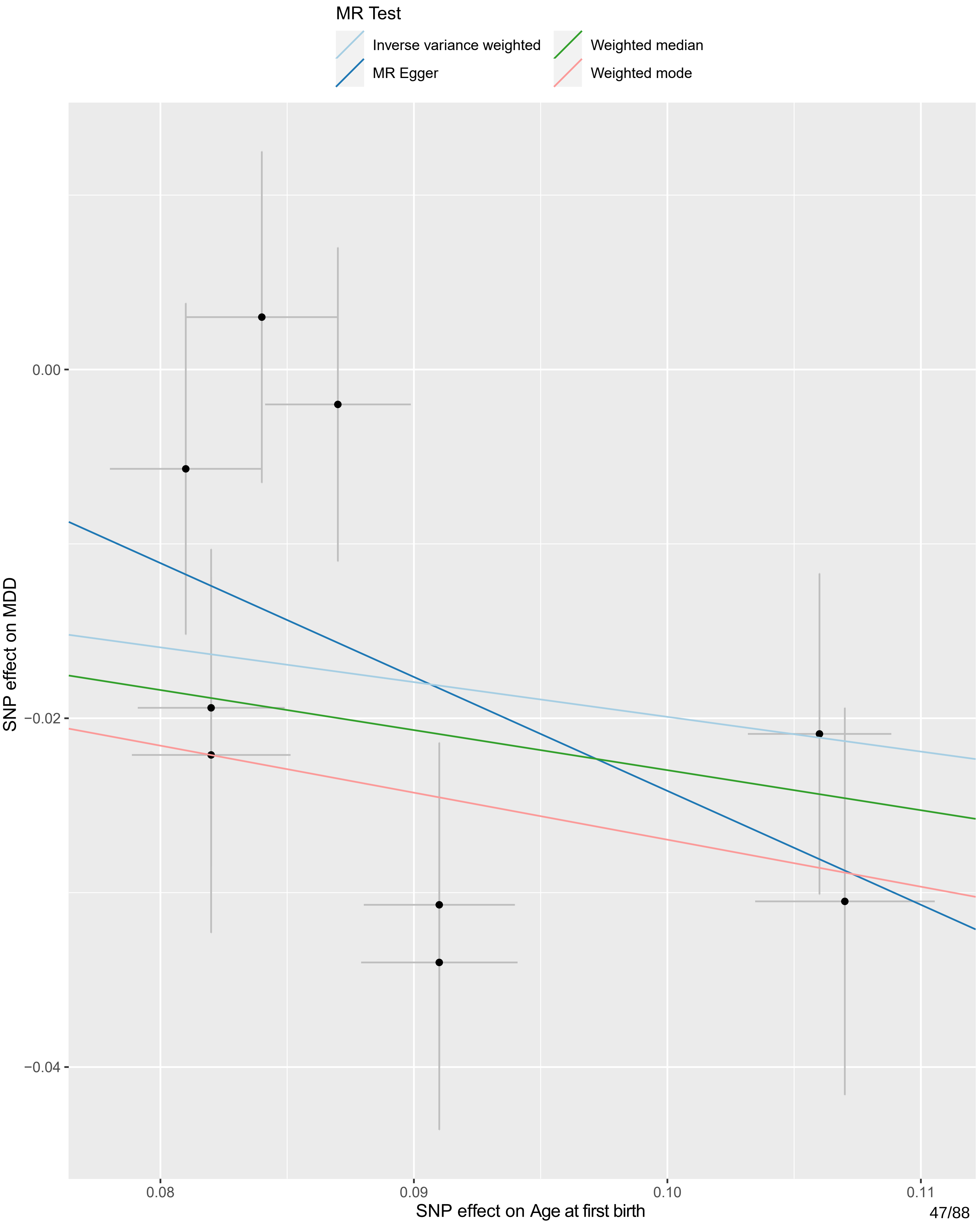

MR Test
Inverse variance weighted
MR Egger
Weighted median
Weighted mode
0.00
−0.02
−0.04
SNP effect on MDD
0.08
0.09
0.10
0.11
SNP effect on Age at first birth

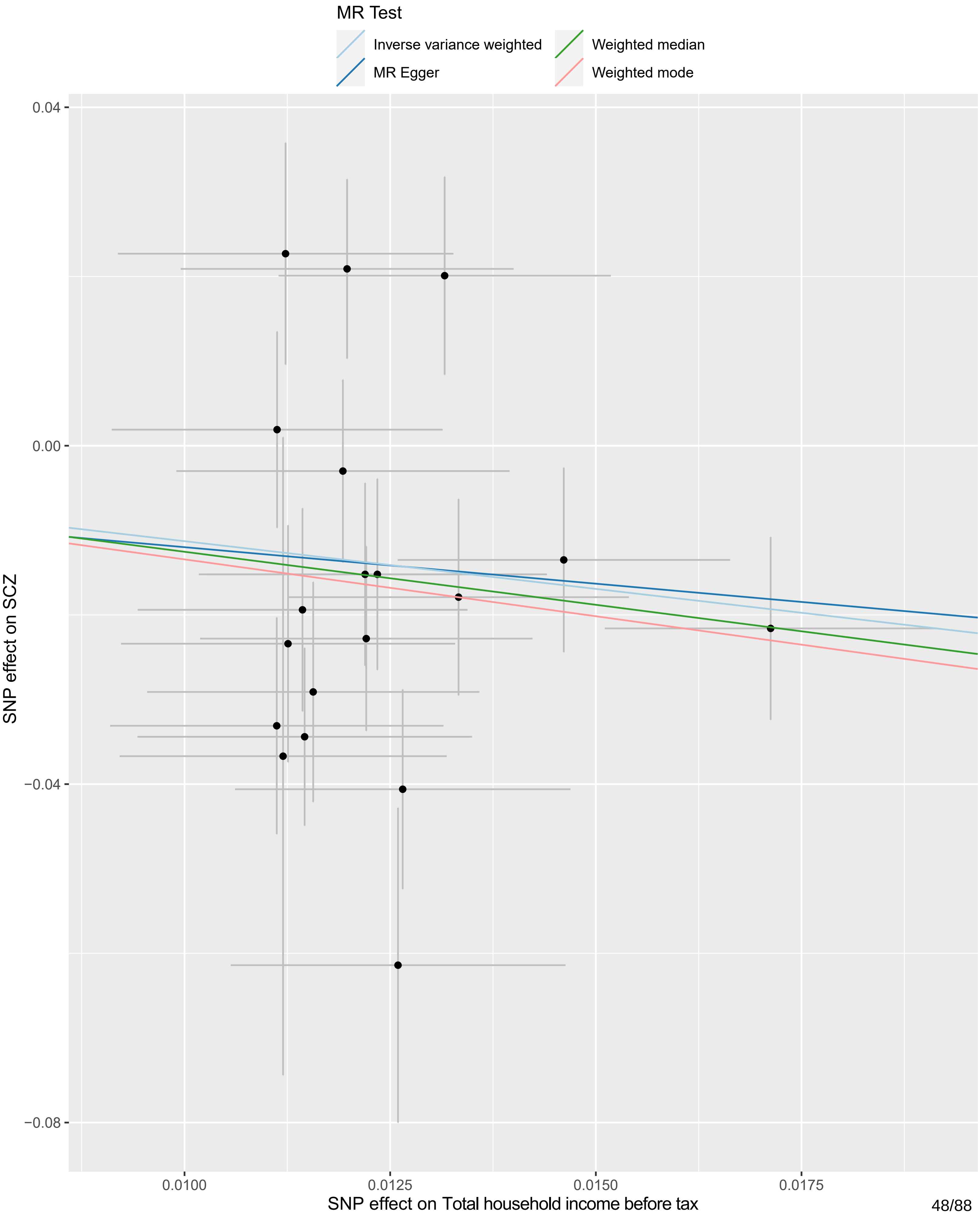

MR Test
Inverse variance weighted
MR Egger
Weighted median
Weighted mode
0.04
0.00
−0.04
−0.08
SNP effect on SCZ
0.0100
0.0125
0.0150
0.0175
SNP effect on Total household income before tax

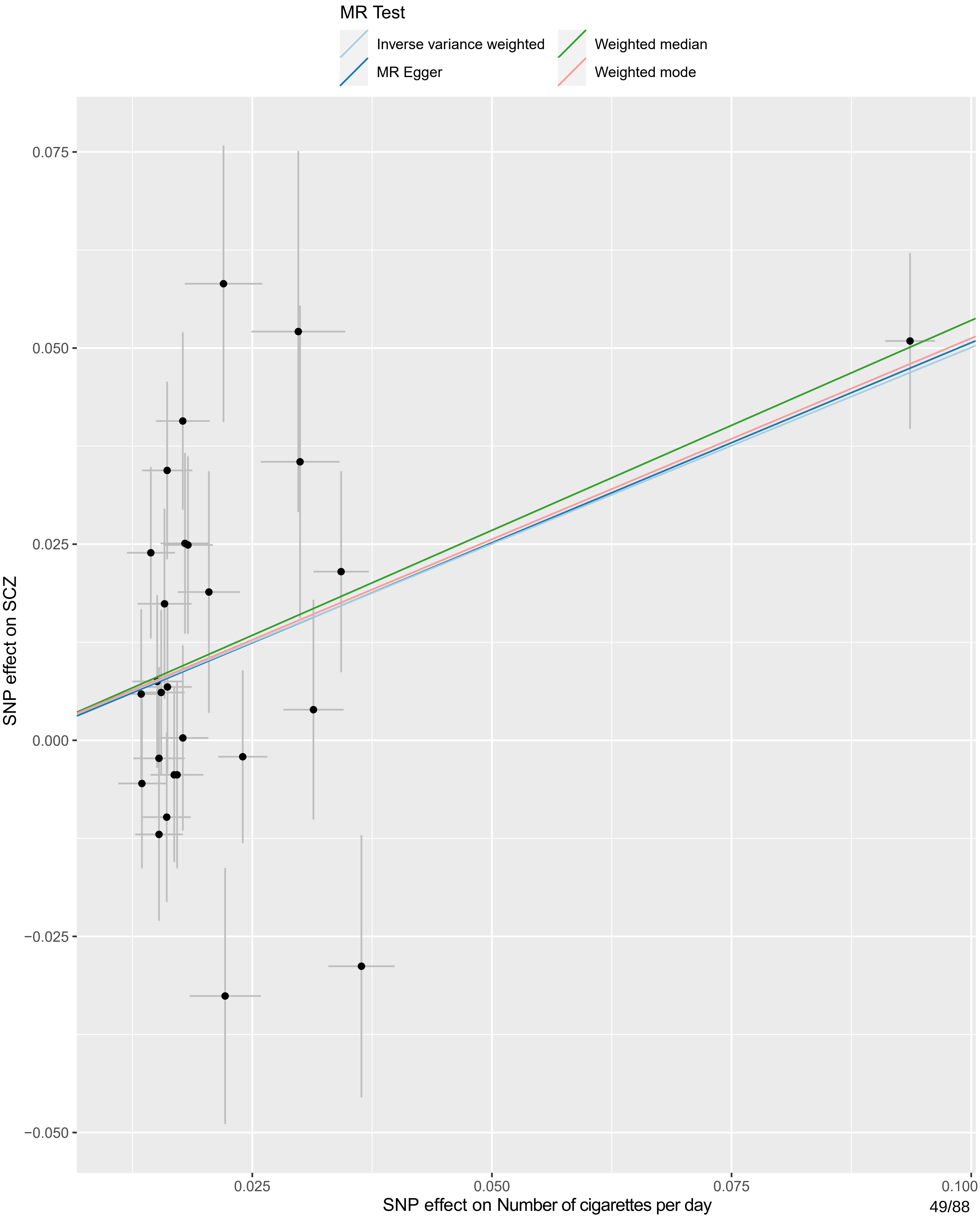

MR Test
Inverse variance weighted
MR Egger
Weighted median
Weighted mode
0.075
0.050
0.025
0.000
−0.025
−0.050
SNP effect on SCZ
0.025
0.050
0.075
0.100
SNP effect on Number of cigarettes per day

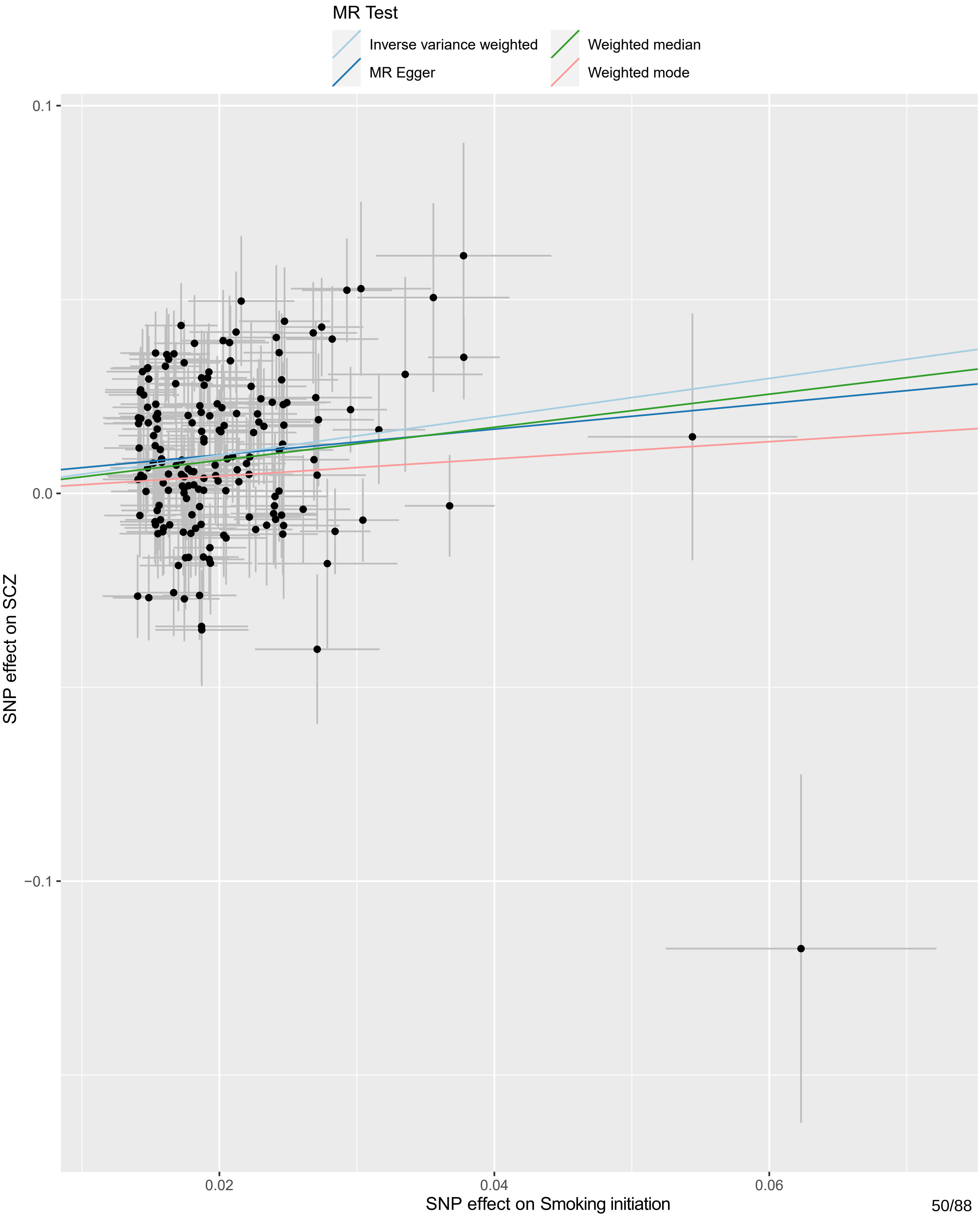

MR Test
Inverse variance weighted
MR Egger
Weighted median
Weighted mode
0.1
0.0
−0.1
SNP effect on SCZ
0.02
0.04
0.06
SNP effect on Smoking initiation

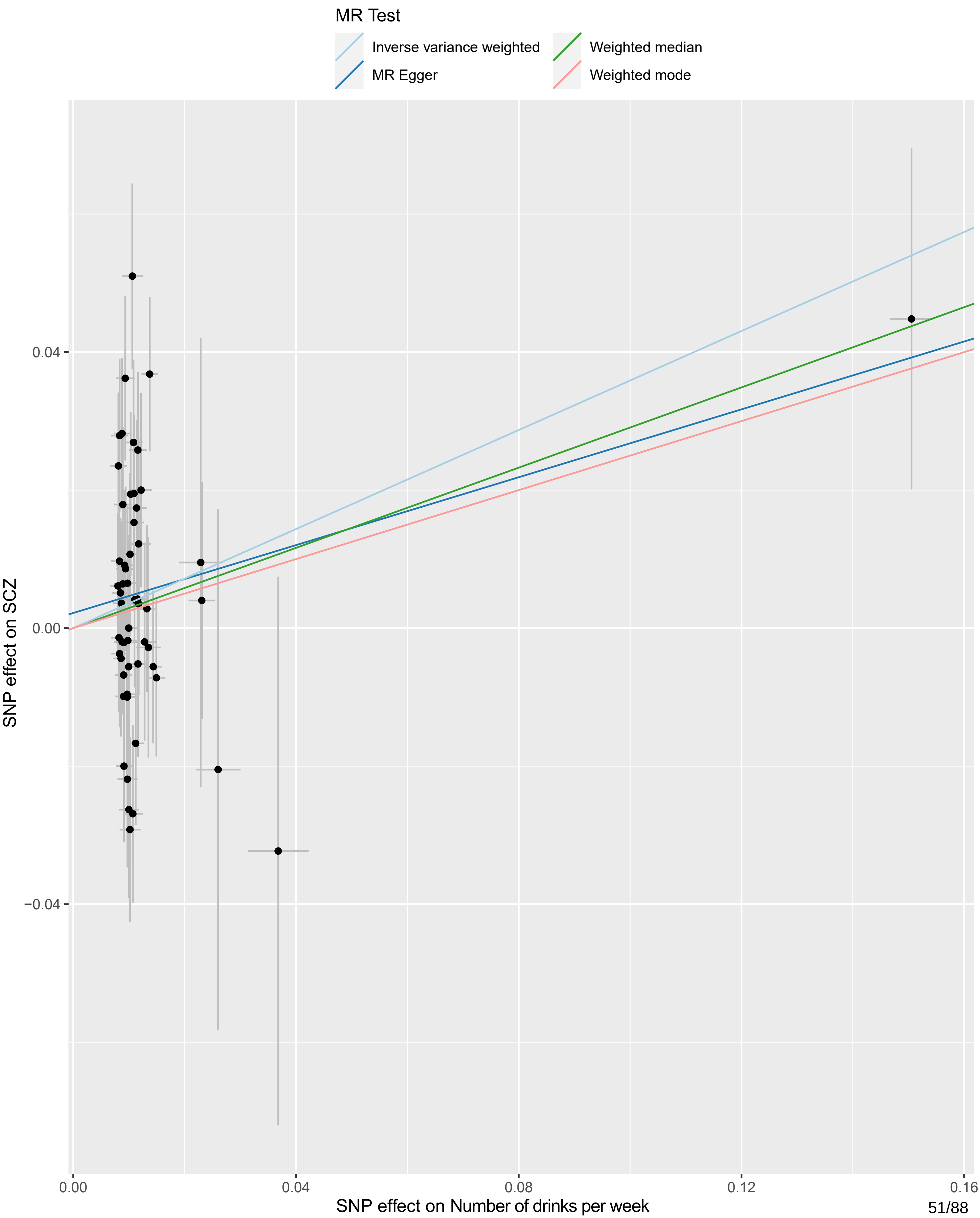
MR Test
Inverse variance weighted
MR Egger
Weighted median
Weighted mode
SNP effect on SCZ
0.04
0.00
−0.04
0.00
0.04
0.08
0.12
0.16
SNP effect on Number of drinks per week

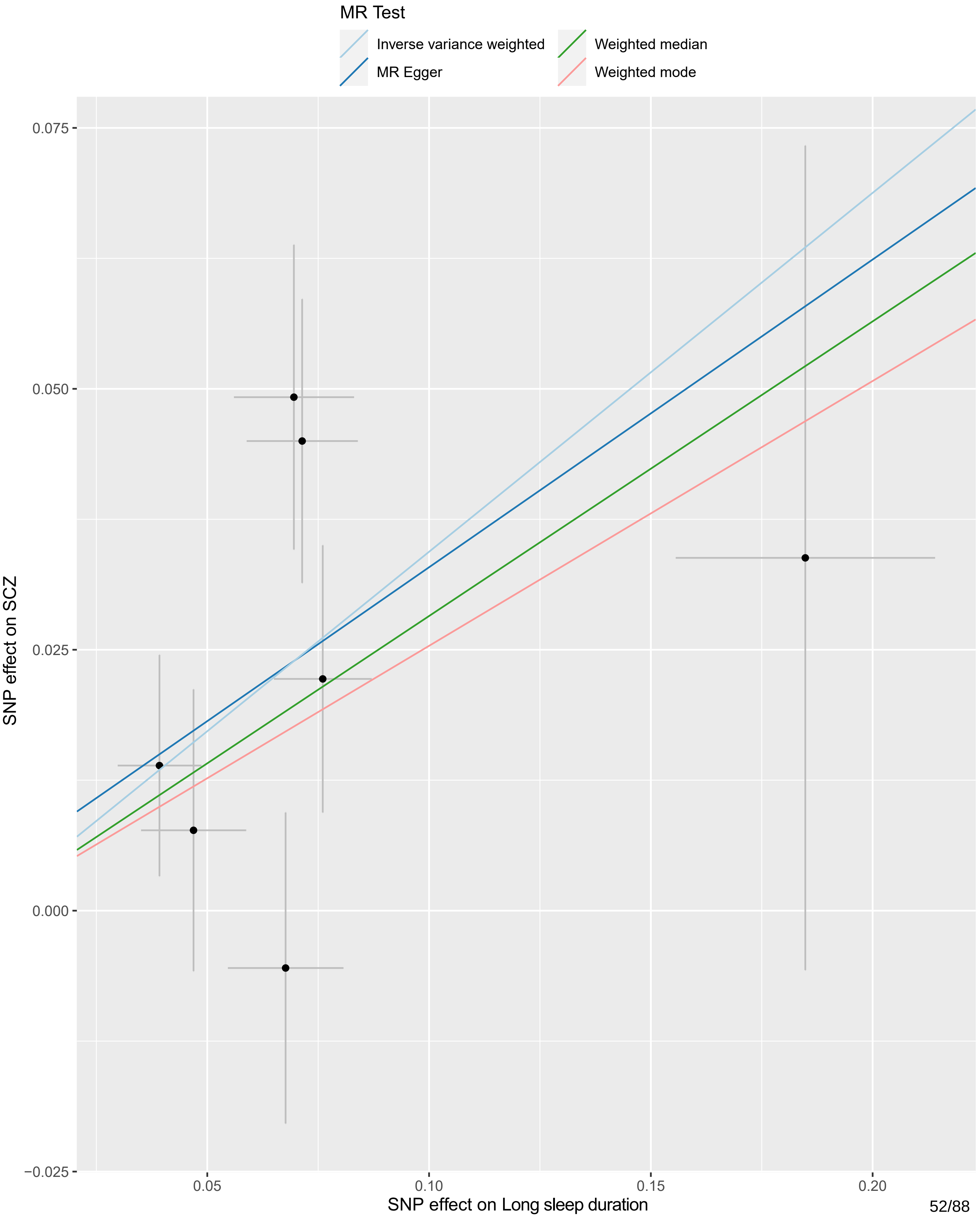
MR Test
Inverse variance weighted
MR Egger
Weighted median
Weighted mode
0.075
0.050
0.025
0.000
−0.025
SNP effect on SCZ
0.05
0.10
0.15
0.20
SNP effect on Long sleep duration

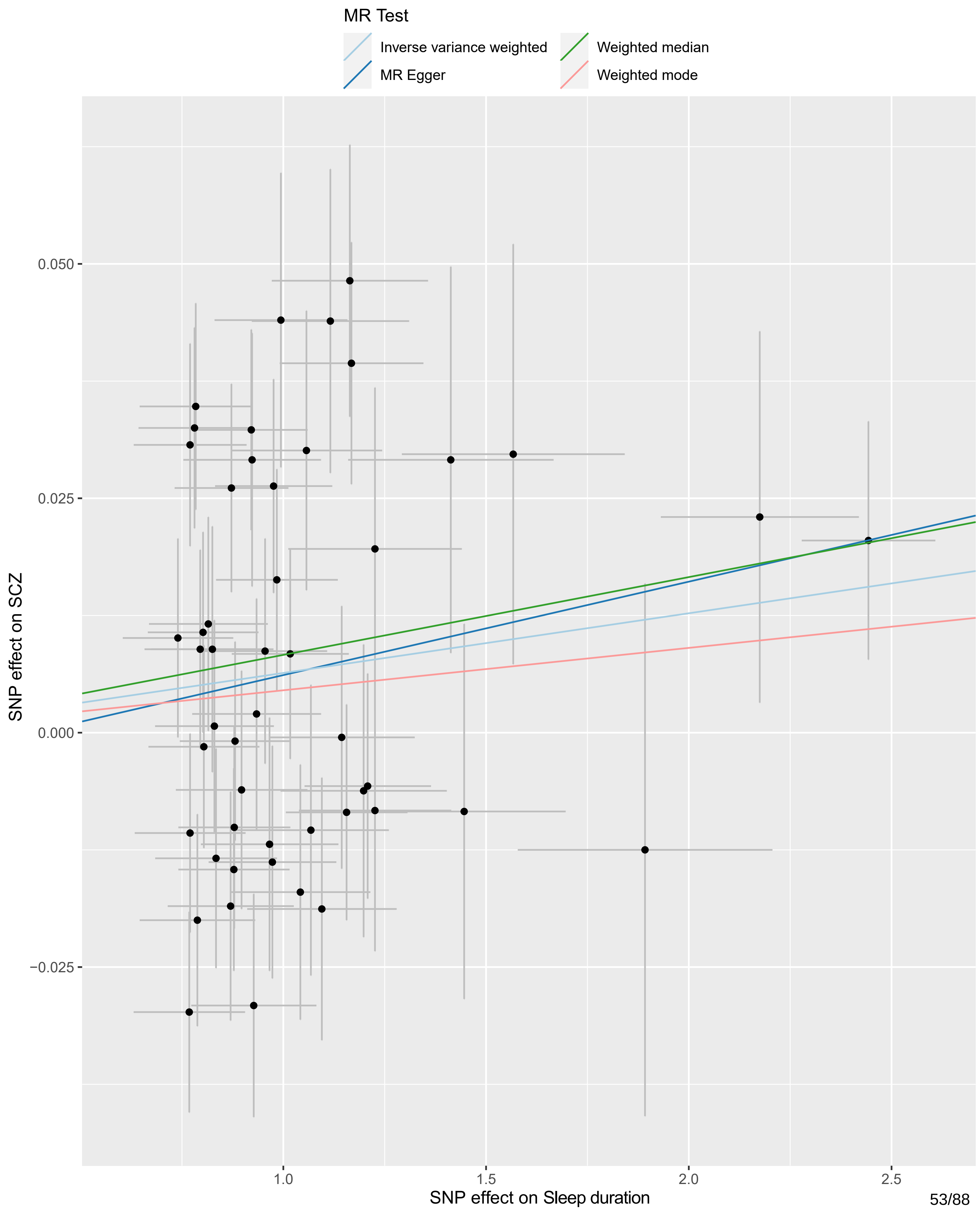
MR Test
Inverse variance weighted
Weighted median
MR Egger
Weighted mode
0.050
0.025
0.000
−0.025
SNP effect on SCZ
1.0
1.5
2.0
2.5
SNP effect on Sleep duration

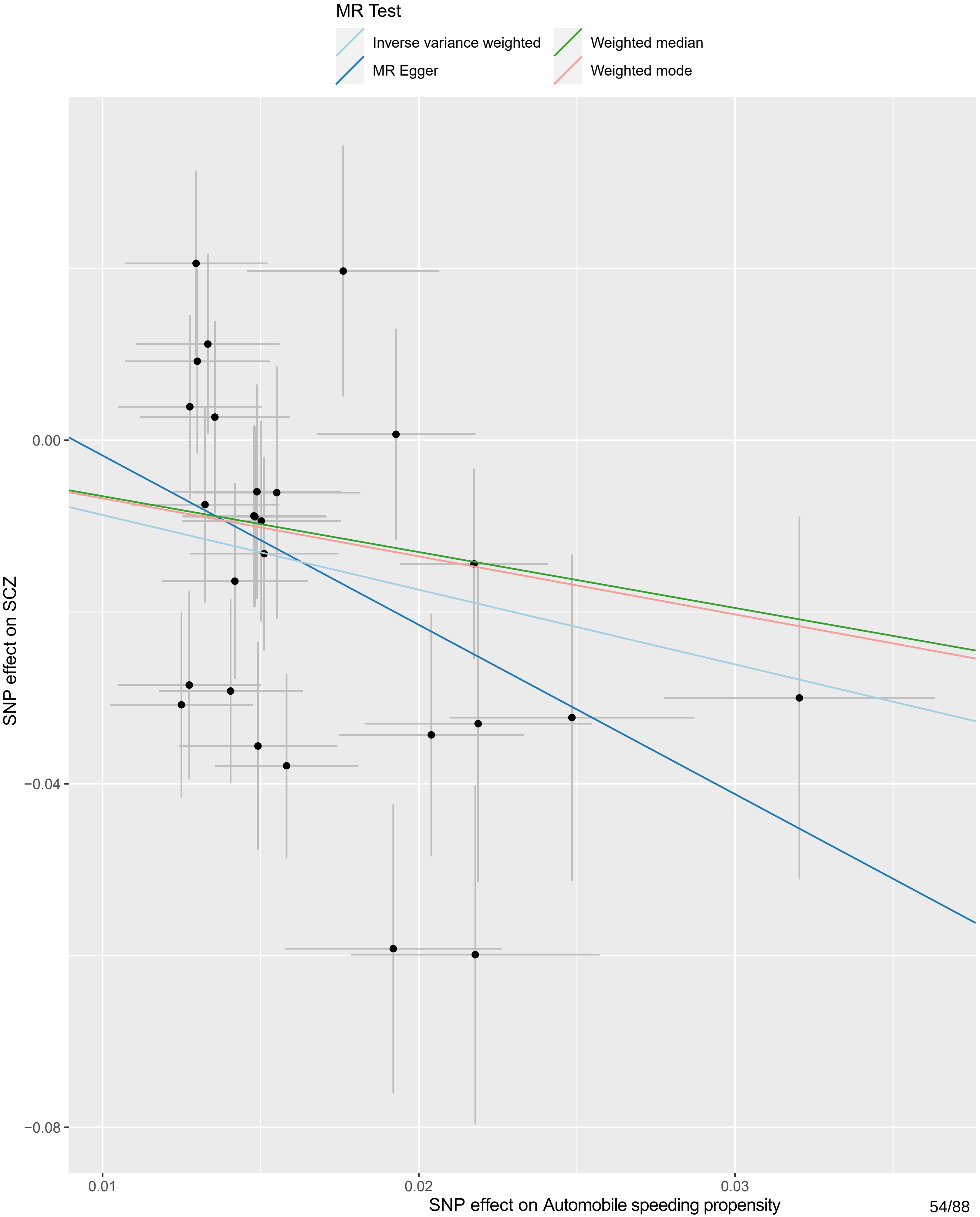

MR Test
Inverse variance weighted
MR Egger
Weighted median
Weighted mode
0.00
−0.04
−0.08
SNP effect on SCZ
0.01
0.02
0.03
SNP effect on Automobile speeding propensity

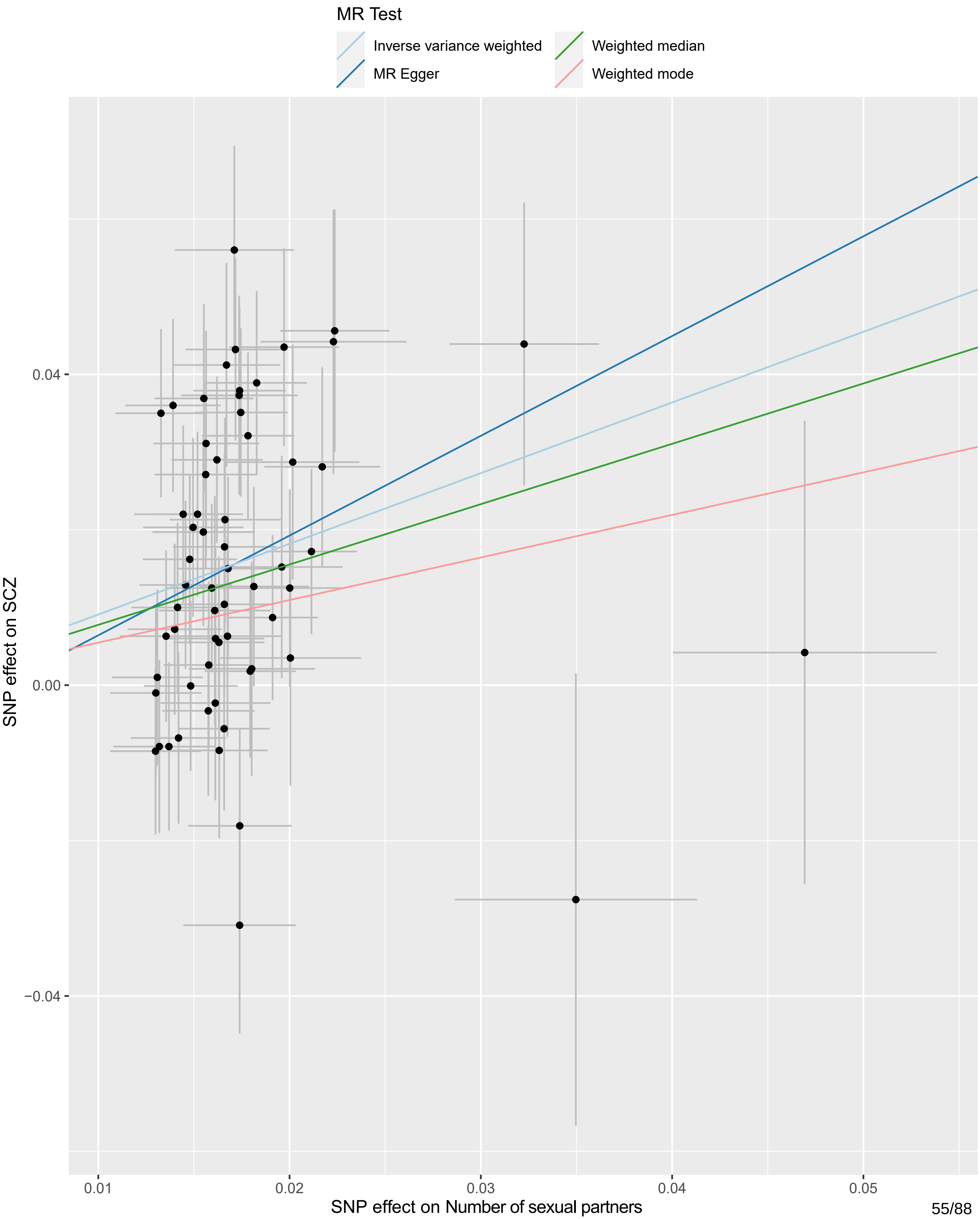
MR Test
Inverse variance weighted
MR Egger
Weighted median
Weighted mode
0.04
0.00
−0.04
SNP effect on SCZ
0.01
0.02
0.03
0.04
0.05
SNP effect on Number of sexual partners

**Figure S2.** Leave-one-out analysis for all significant causal relationships detected in primary analysis. Within each panel, the black points represent the causal estimate of association between a specific exposure and target mental disorder after discarding each SNP in turn. Red points represent the overall causal estimate using the random-effects inverse variance weighted. Horizontal lines denote 95% confidence intervals.

BIP, bipolar disorder; MDD, major depressive disorder; SCZ, schizophrenia.

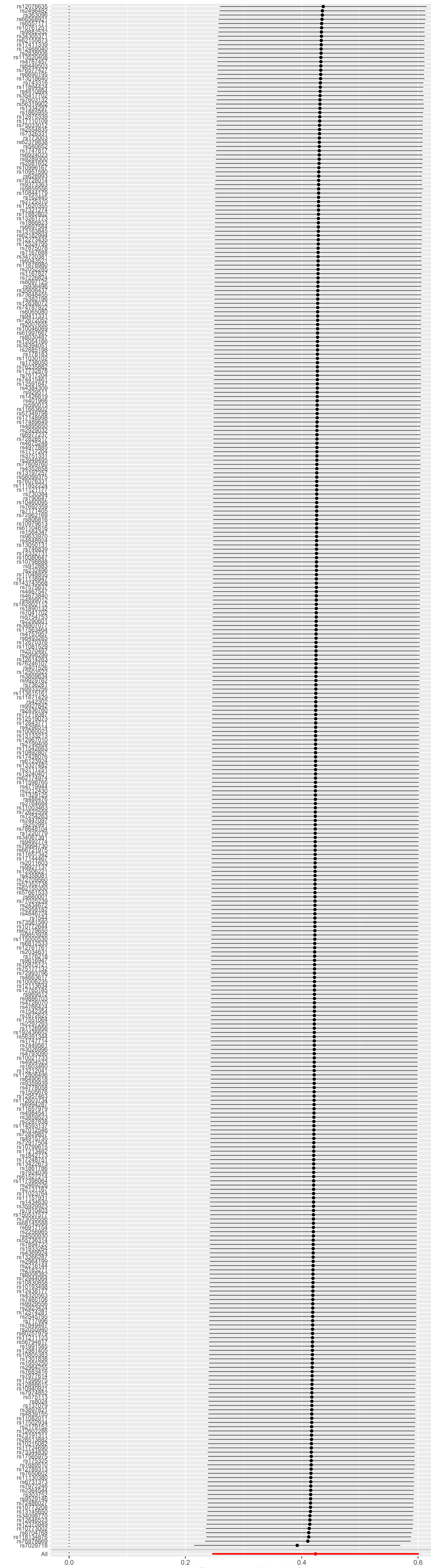

MR leave−one−out sensitivity analysis for
'Educational attainment' on 'BIP'

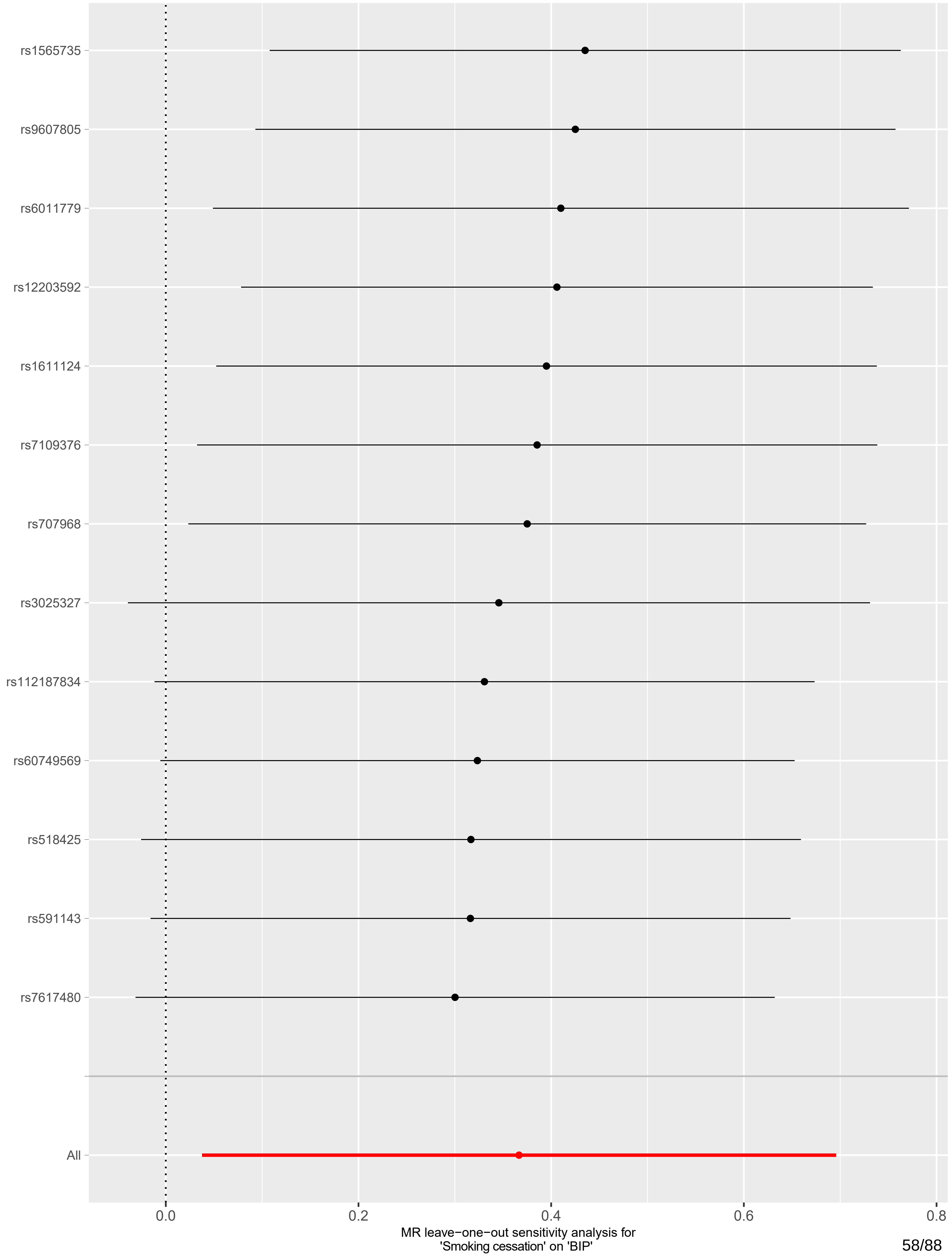

rs1565735
rs9607805
rs6011779
rs12203592
rs1611124
rs7109376
rs707968
rs3025327
rs112187834
rs60749569
rs518425
rs591143
rs7617480
All
0.0
0.2
0.4
0.6
0.8
MR leave−one−out sensitivity analysis for
'Smoking cessation' on 'BIP'

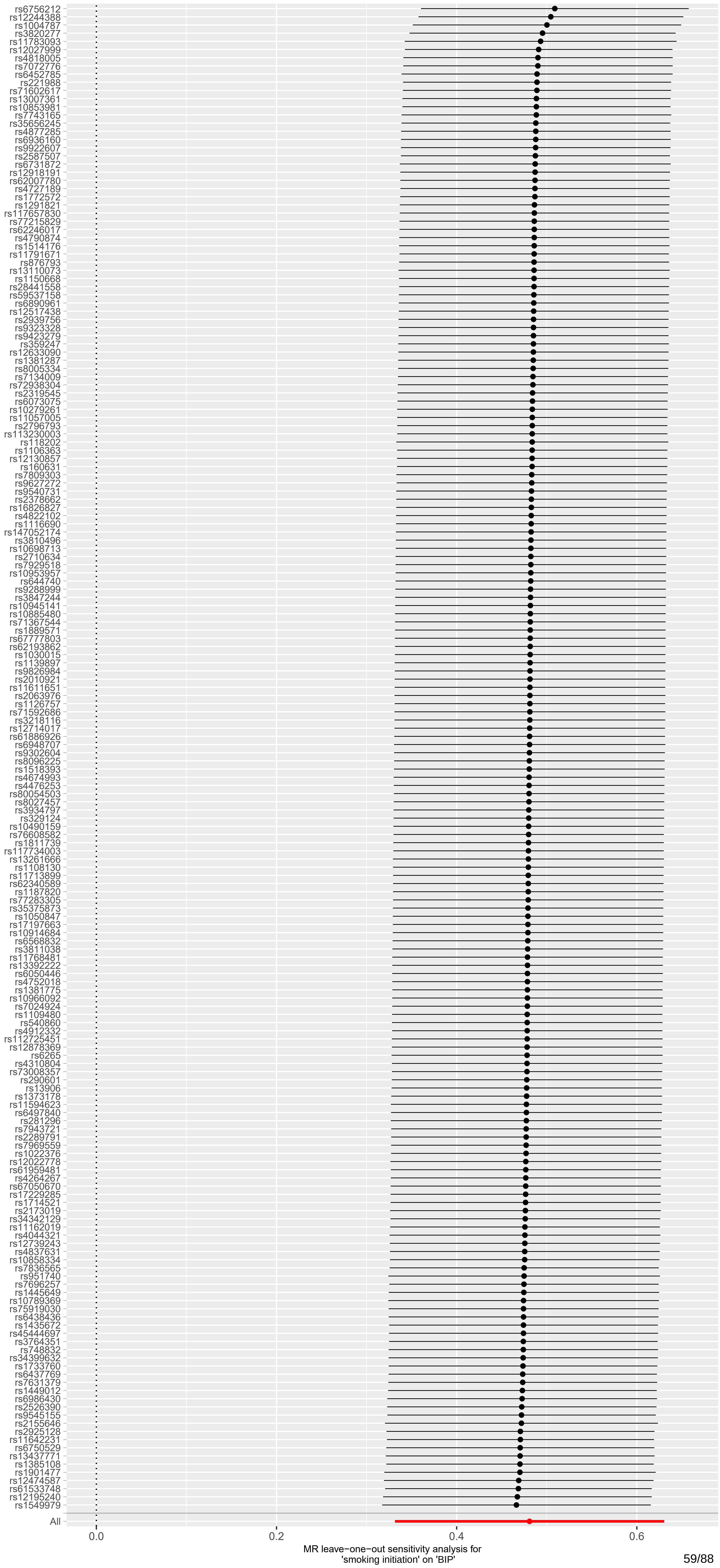
rs6756212
rs12244388
rs1004787
rs3820277
rs11783093
rs12027999
rs4818005
rs7072776
rs6452785
rs221988
rs71602617
rs13007361
rs10853981
rs7743165
rs35656245
rs4877285
rs6936160
rs9922607
rs2587507
rs6731872
rs12918191
rs62007780
rs4727189
rs1772572
rs1291821
rs117657830
rs77215829
rs62246017
rs4790874
rs1514176
rs11791671
rs876793
rs13110073
rs1150668
rs28441558
rs59537158
rs6890961
rs12517438
rs2939756
rs9323328
rs9423279
rs359247
rs12633090
rs1381287
rs8005334
rs7134009
rs72938304
rs2319545
rs6073075
rs10279261
rs11057005
rs2796793
rs113230003
rs118202
rs1106363
rs12130857
rs160631
rs7809303
rs9627272
rs9540731
rs2378662
rs16826827
rs4822102
rs1116690
rs147052174
rs3810496
rs10698713
rs2710634
rs7929518
rs10953957
rs644740
rs9288999
rs3847244
rs10945141
rs10885480
rs71367544
rs1889571
rs67777803
rs62193862
rs1030015
rs1139897
rs9826984
rs2010921
rs11611651
rs2063976
rs1126757
rs71592686
rs3218116
rs12714017
rs61886926
rs6948707
rs9302604
rs8096225
rs1518393
rs4674993
rs4476253
rs80054503
rs8027457
rs3934797
rs329124
rs10490159
rs76608582
rs1811739
rs117734003
rs13261666
rs1108130
rs11713899
rs62340589
rs1187820
rs77283305
rs35375873
rs1050847
rs17197663
rs10914684
rs6568832
rs3811038
rs11768481
rs13392222
rs6050446
rs4752018
rs1381775
rs10966092
rs7024924
rs1109480
rs540860
rs4912332
rs112725451
rs12878369
rs6265
rs4310804
rs73008357
rs290601
rs13906
rs1373178
rs11594623
rs6497840
rs281296
rs7943721
rs2289791
rs7969559
rs1022376
rs12022778
rs61959481
rs4264267
rs67050670
rs17229285
rs1714521
rs2173019
rs34342129
rs11162019
rs4044321
rs12739243
rs4837631
rs10858334
rs7836565
rs951740
rs7696257
rs1445649
rs10789369
rs75919030
rs6438436
rs1435672
rs45444697
rs3764351
rs748832
rs34399632
rs1733760
rs6437769
rs7631379
rs1449012
rs6986430
rs2526390
rs9545155
rs2155646
rs2925128
rs11642231
rs6750529
rs13437771
rs1385108
rs1901477
rs12474587
rs61533748
rs12195240
rs1549979
All
0.0
0.2
0.4
0.6
MR leave−one−out sensitivity analysis for
'smoking initiation' on 'BIP'

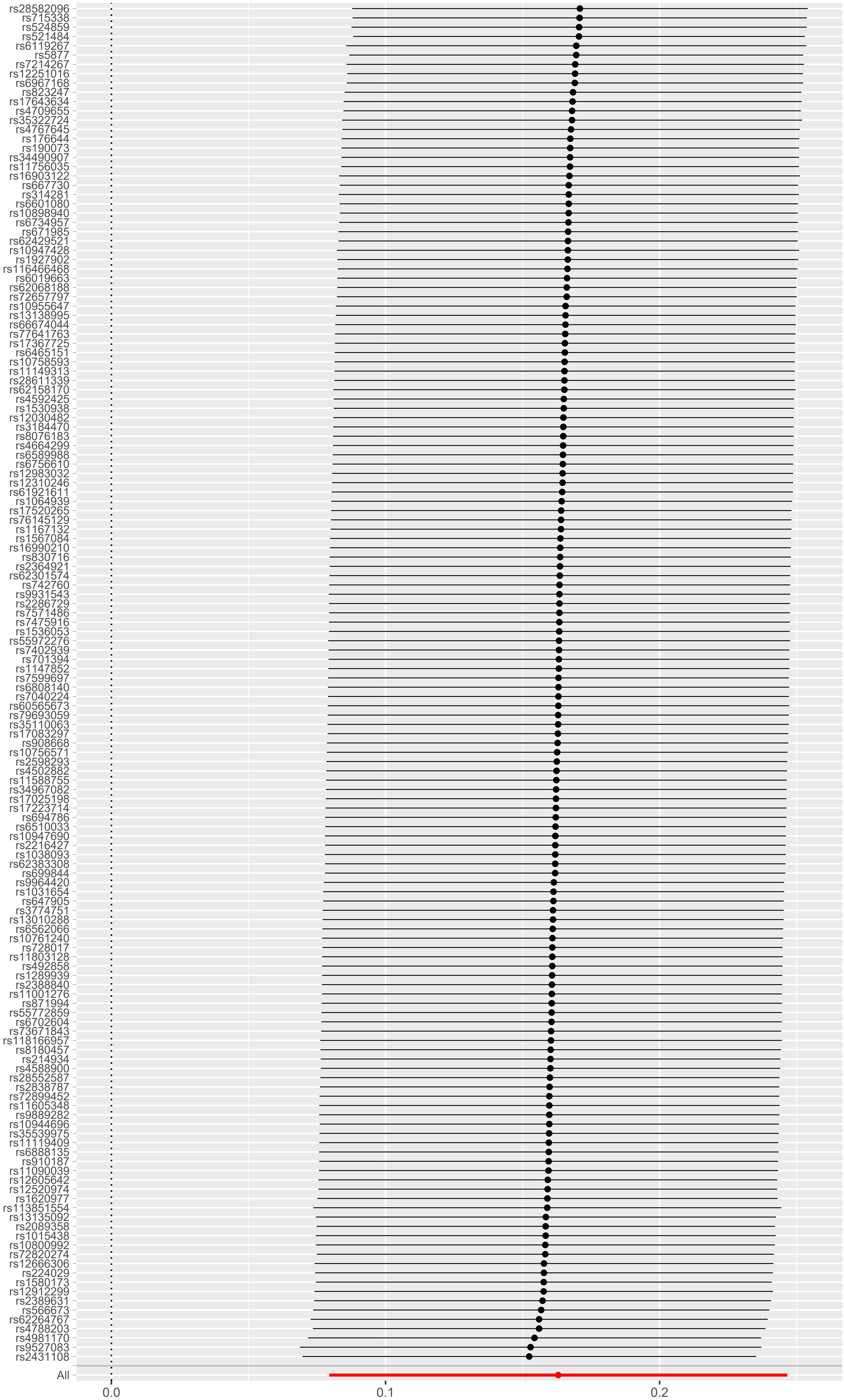


MR leave−one−out sensitivity analysis for
'Insomnia' on 'BIP'

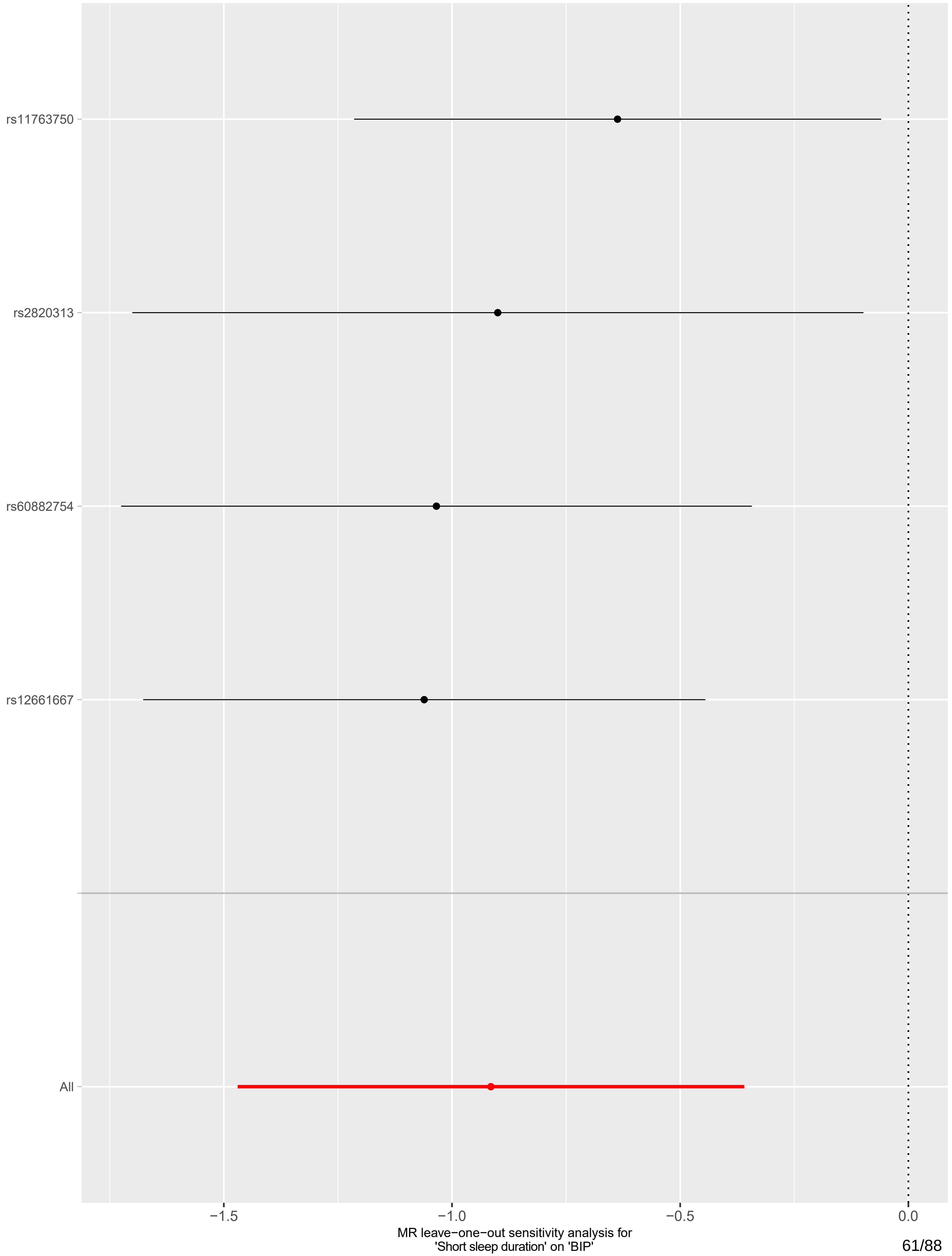
rs11763750
rs2820313
rs60882754
rs12661667
All
−1.5
−1.0
−0.5
0.0
MR leave−one−out sensitivity analysis for
'Short sleep duration' on 'BIP'

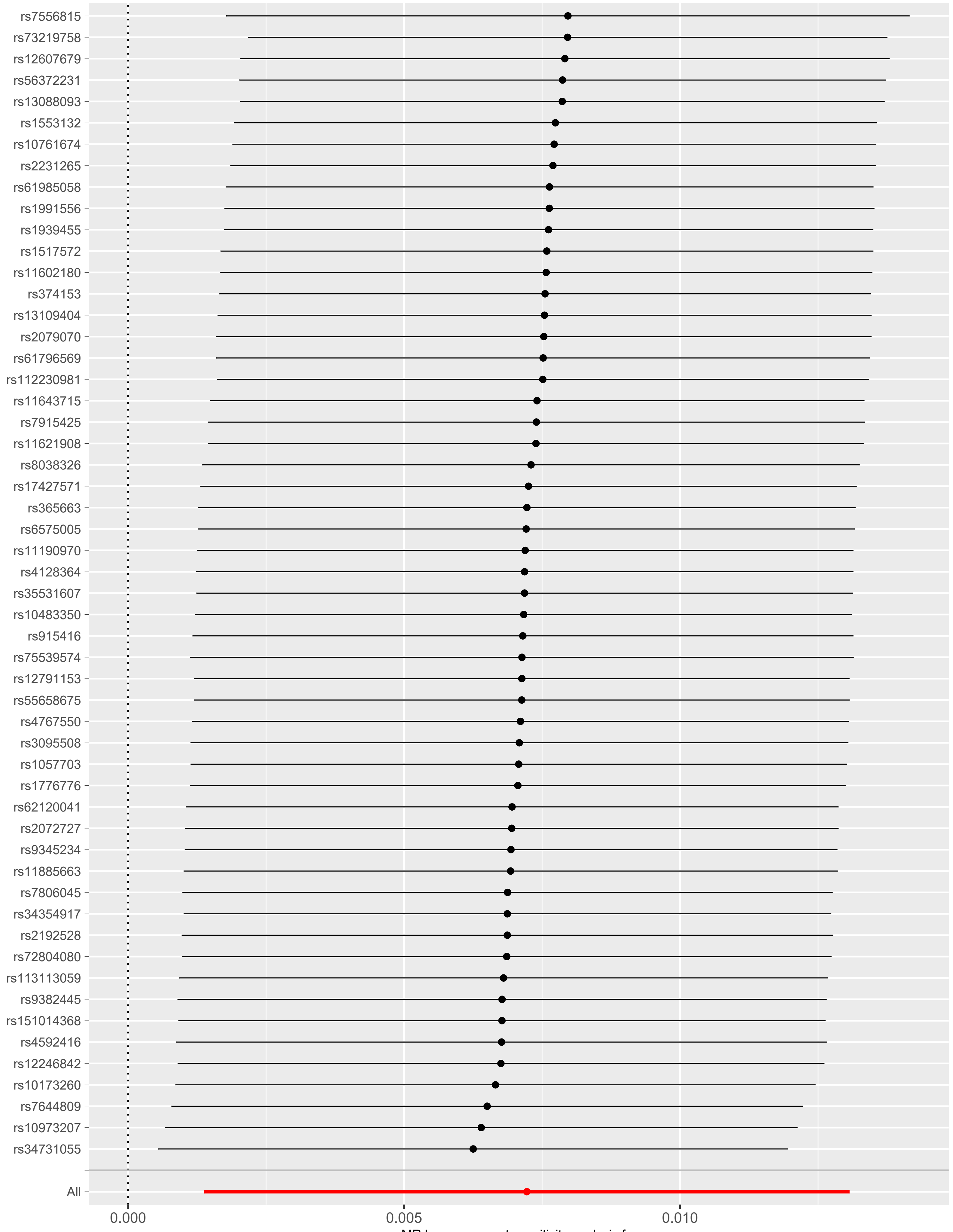

MR leave−one−out sensitivity analysis for
'Sleep duration' on 'BIP'

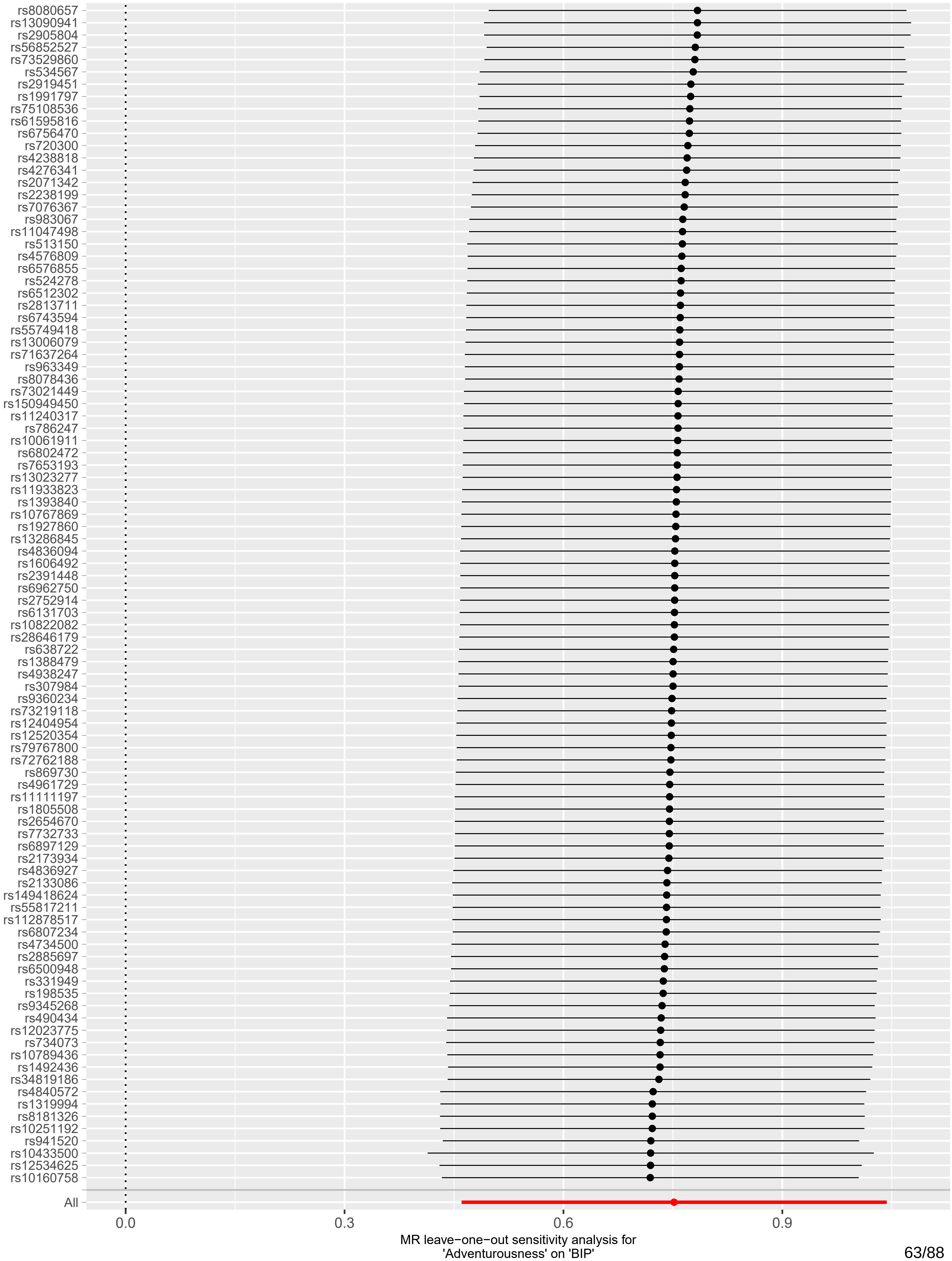
rs8080657
rs13090941
rs2905804
rs56852527
rs73529860
rs534567
rs2919451
rs1991797
rs75108536
rs61595816
rs6756470
rs720300
rs4238818
rs4276341
rs2071342
rs2238199
rs7076367
rs983067
rs11047498
rs513150
rs4576809
rs6576855
rs524278
rs6512302
rs2813711
rs6743594
rs55749418
rs13006079
rs71637264
rs963349
rs8078436
rs73021449
rs150949450
rs11240317
rs786247
rs10061911
rs6802472
rs7653193
rs13023277
rs11933823
rs1393840
rs10767869
rs1927860
rs13286845
rs4836094
rs1606492
rs2391448
rs6962750
rs2752914
rs6131703
rs10822082
rs28646179
rs638722
rs1388479
rs4938247
rs307984
rs9360234
rs73219118
rs12404954
rs12520354
rs79767800
rs72762188
rs869730
rs4961729
rs11111197
rs1805508
rs2654670
rs7732733
rs6897129
rs2173934
rs4836927
rs2133086
rs149418624
rs55817211
rs112878517
rs6807234
rs4734500
rs2885697
rs6500948
rs331949
rs198535
rs9345268
rs490434
rs12023775
rs734073
rs10789436
rs1492436
rs34819186
rs4840572
rs1319994
rs8181326
rs10251192
rs941520
rs10433500
rs12534625
rs10160758
All
0.0
0.3
0.6
0.9
MR leave−one−out sensitivity analysis for
'Adventurousness' on 'BIP'

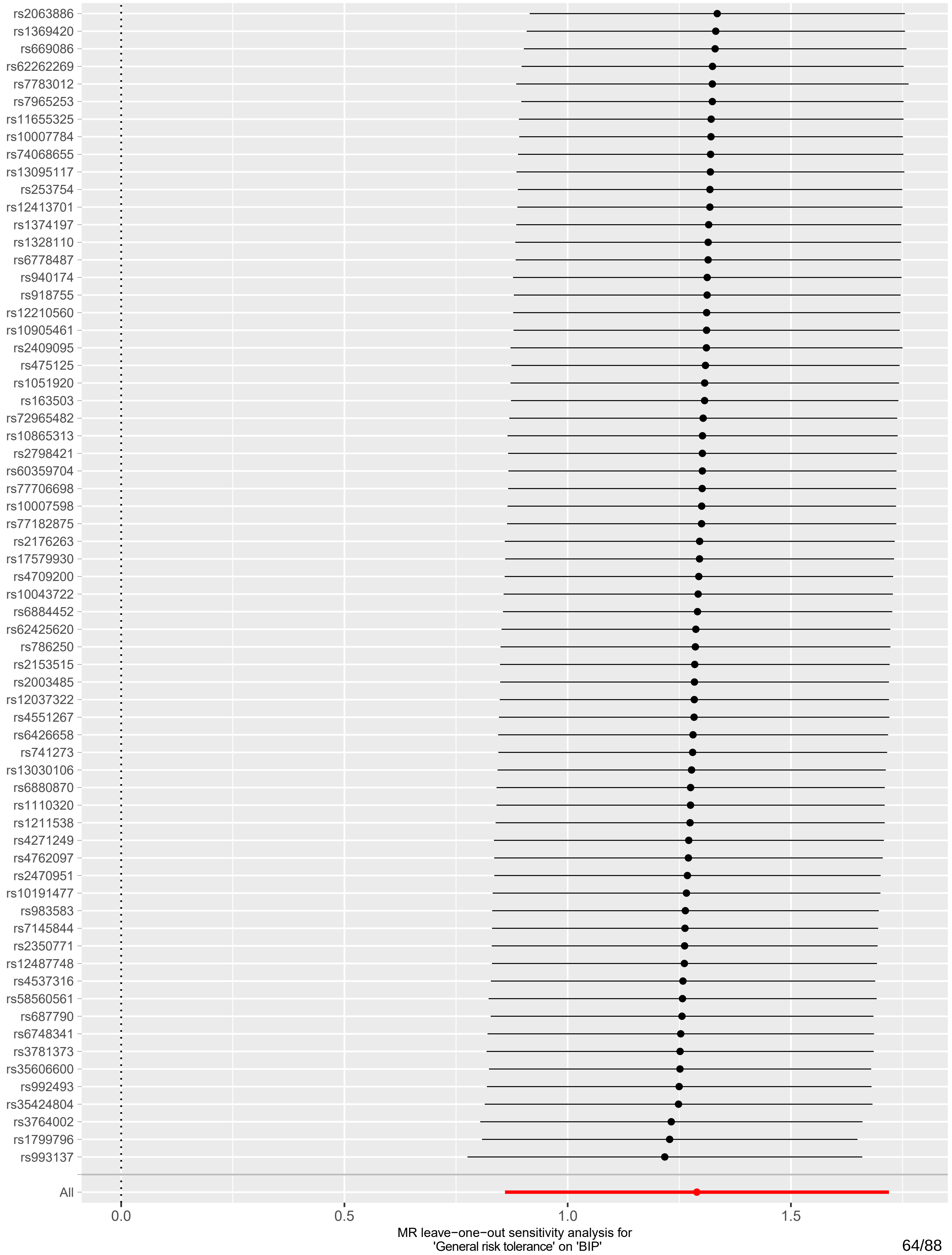

rs2063886
rs1369420
rs669086
rs62262269
rs7783012
rs7965253
rs11655325
rs10007784
rs74068655
rs13095117
rs253754
rs12413701
rs1374197
rs1328110
rs6778487
rs940174
rs918755
rs12210560
rs10905461
rs2409095
rs475125
rs1051920
rs163503
rs72965482
rs10865313
rs2798421
rs60359704
rs77706698
rs10007598
rs77182875
rs2176263
rs17579930
rs4709200
rs10043722
rs6884452
rs62425620
rs786250
rs2153515
rs2003485
rs12037322
rs4551267
rs6426658
rs741273
rs13030106
rs6880870
rs1110320
rs1211538
rs4271249
rs4762097
rs2470951
rs10191477
rs983583
rs7145844
rs2350771
rs12487748
rs4537316
rs58560561
rs687790
rs6748341
rs3781373
rs35606600
rs992493
rs35424804
rs3764002
rs1799796
rs993137
All
0.0
0.5
1.0
1.5
MR leave−one−out sensitivity analysis for
'General risk tolerance' on 'BIP'

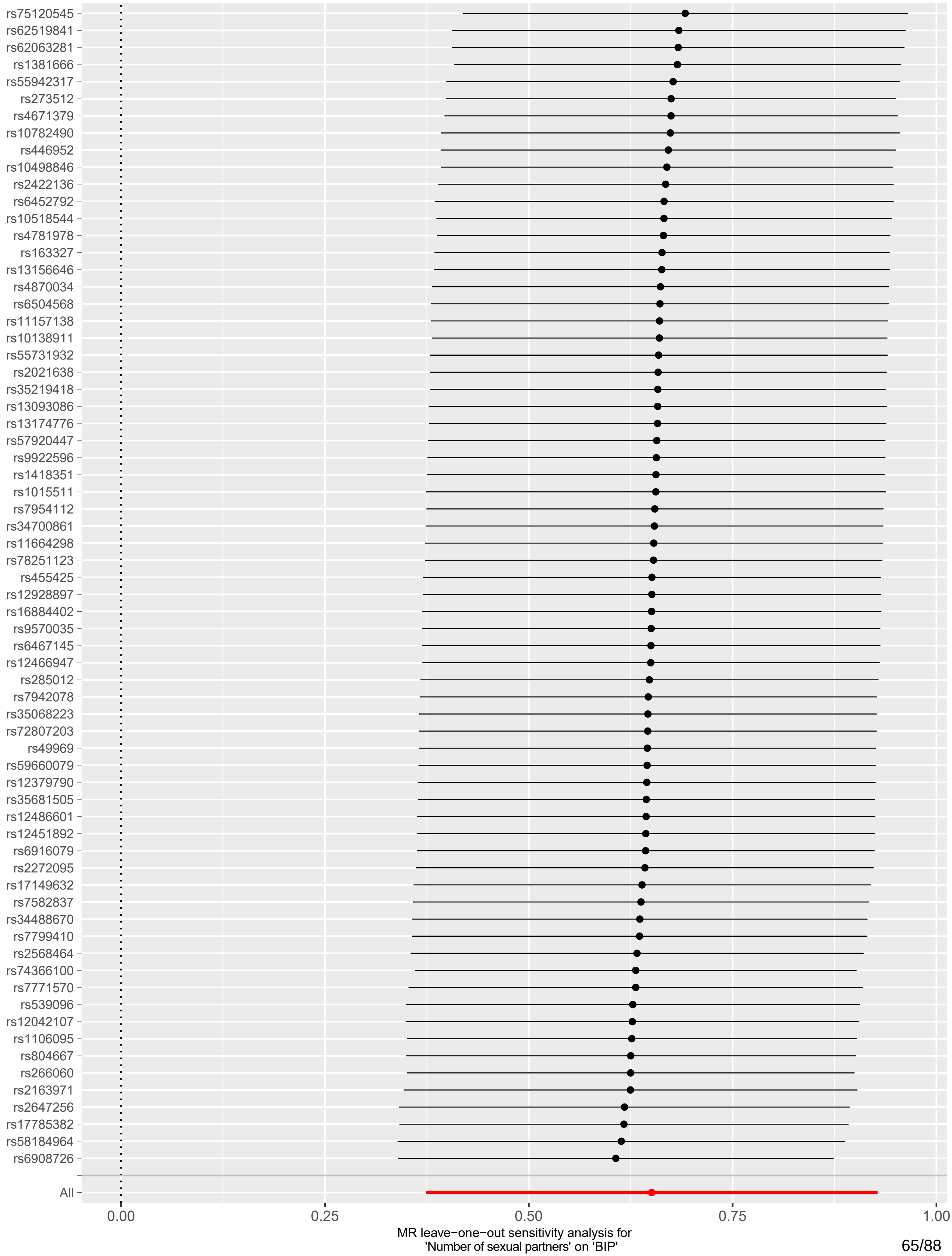

rs75120545
rs62519841
rs62063281
rs1381666
rs55942317
rs273512
rs4671379
rs10782490
rs446952
rs10498846
rs2422136
rs6452792
rs10518544
rs4781978
rs163327
rs13156646
rs4870034
rs6504568
rs11157138
rs10138911
rs55731932
rs2021638
rs35219418
rs13093086
rs13174776
rs57920447
rs9922596
rs1418351
rs1015511
rs7954112
rs34700861
rs11664298
rs78251123
rs455425
rs12928897
rs16884402
rs9570035
rs6467145
rs12466947
rs285012
rs7942078
rs35068223
rs72807203
rs49969
rs59660079
rs12379790
rs35681505
rs12486601
rs12451892
rs6916079
rs2272095
rs17149632
rs7582837
rs34488670
rs7799410
rs2568464
rs74366100
rs7771570
rs539096
rs12042107
rs1106095
rs804667
rs266060
rs2163971
rs2647256
rs17785382
rs58184964
rs6908726
All
0.00
0.25
0.50
0.75
1.00
MR leave−one−out sensitivity analysis for
'Number of sexual partners' on 'BIP'

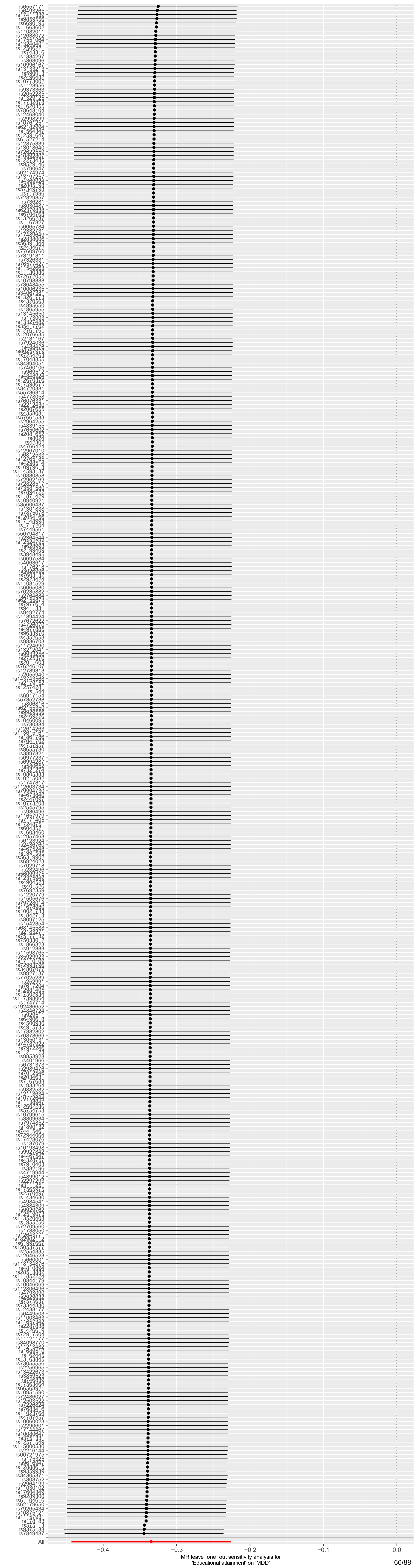
All
-0.4
-0.3
-0.2
-0.1
0.0
MR leave−one−out sensitivity analysis for
'Educational attainment' on 'MDD'

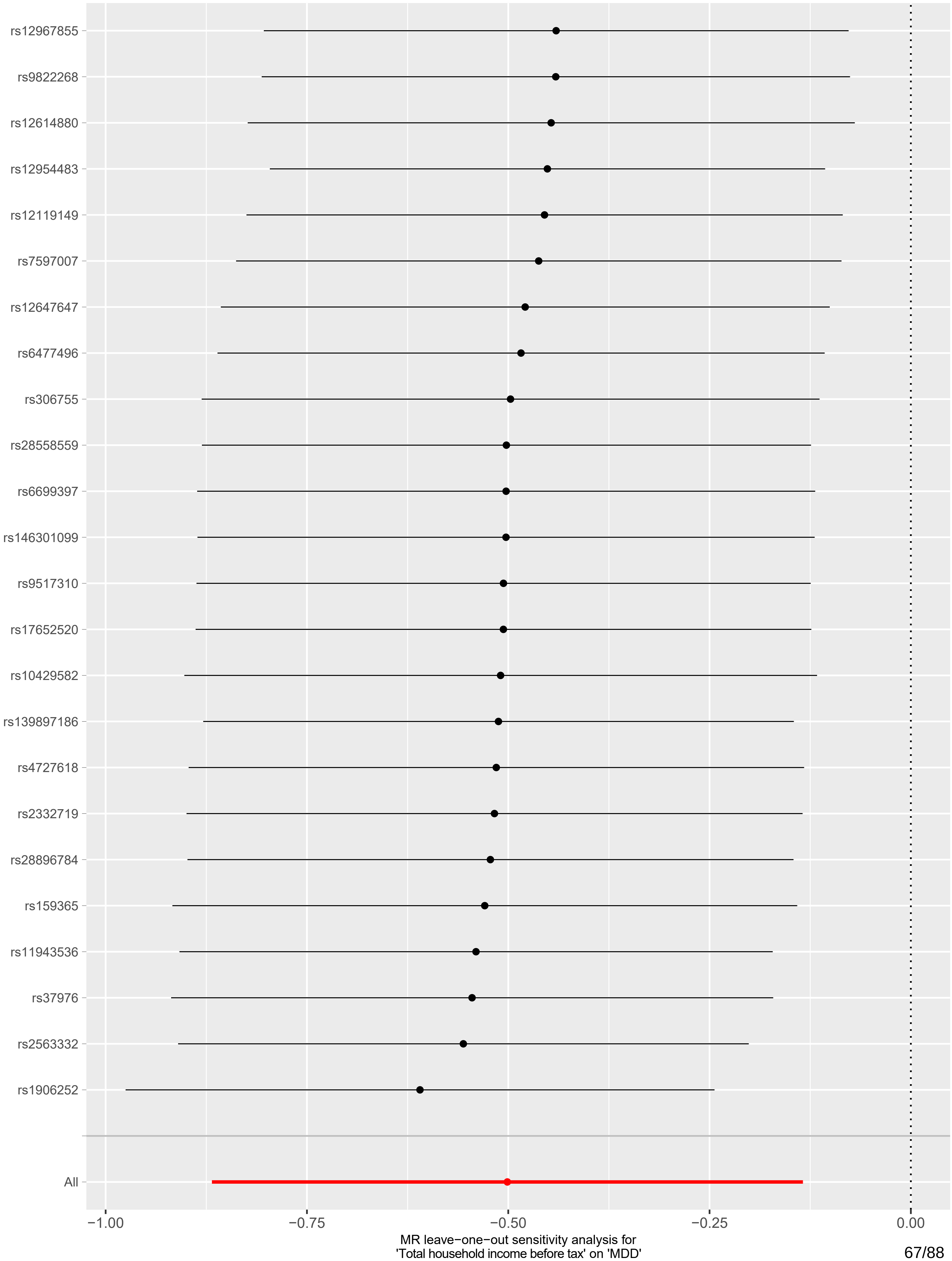
rs12967855
rs9822268
rs12614880
rs12954483
rs12119149
rs7597007
rs12647647
rs6477496
rs306755
rs28558559
rs6699397
rs146301099
rs9517310
rs17652520
rs10429582
rs139897186
rs4727618
rs2332719
rs28896784
rs159365
rs11943536
rs37976
rs2563332
rs1906252
All
−1.00
−0.75
−0.50
−0.25
0.00
MR leave−one−out sensitivity analysis for
'Total household income before tax' on 'MDD'

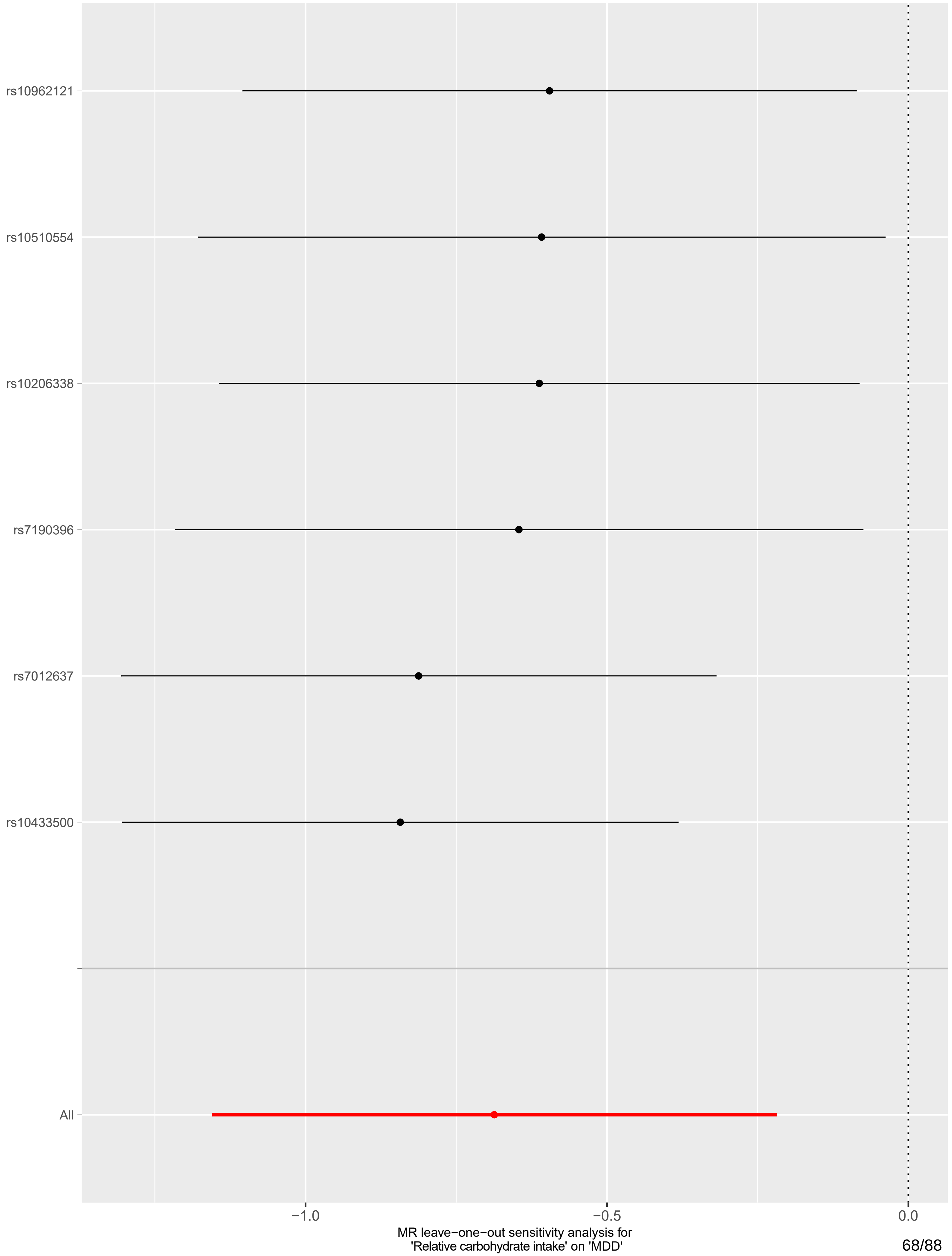

rs10962121
rs10510554
rs10206338
rs7190396
rs7012637
rs10433500
All
−1.0
−0.5
0.0
MR leave−one−out sensitivity analysis for
'Relative carbohydrate intake' on 'MDD'

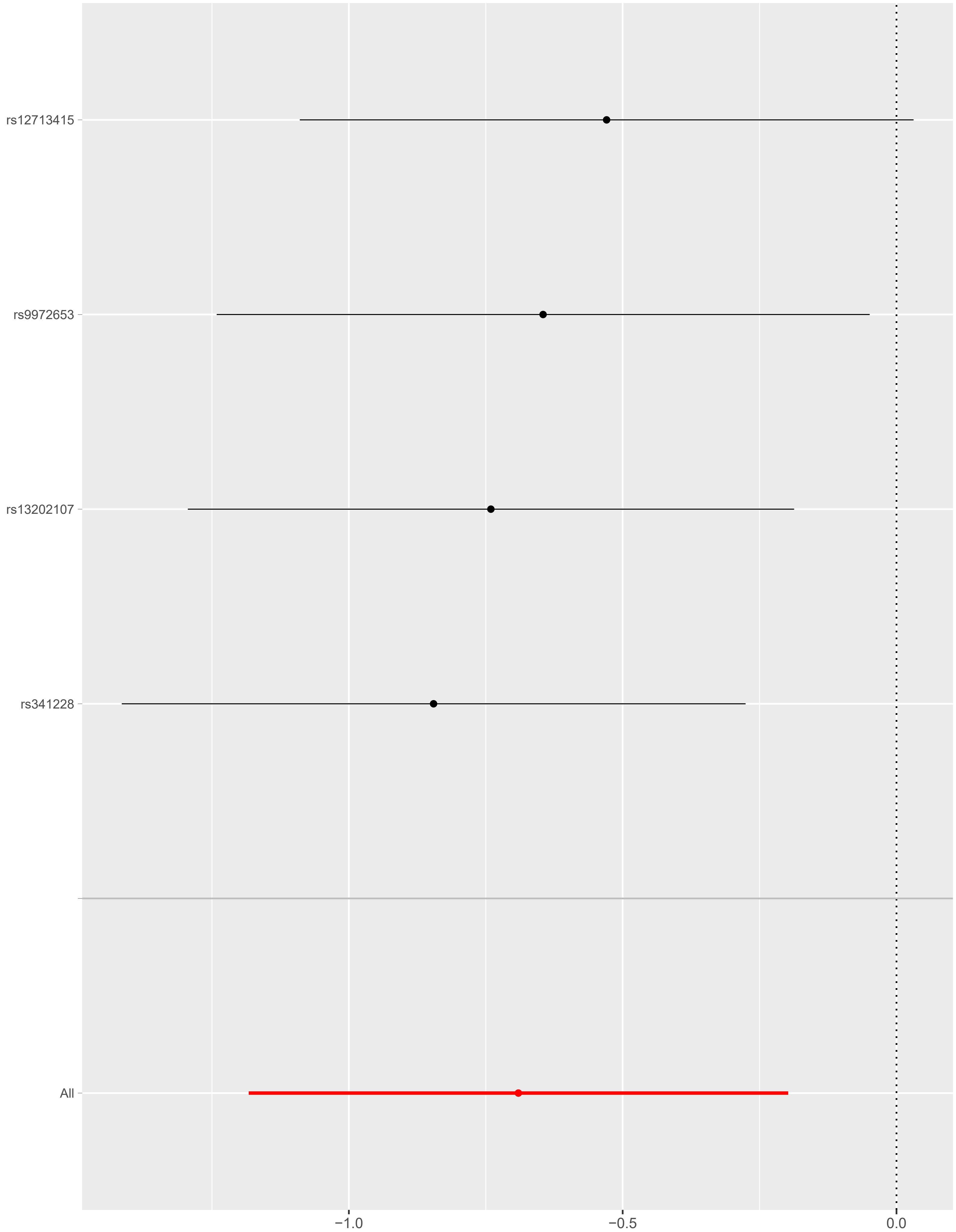

MR leave−one−out sensitivity analysis for
'Relative sugar intake' on 'MDD'

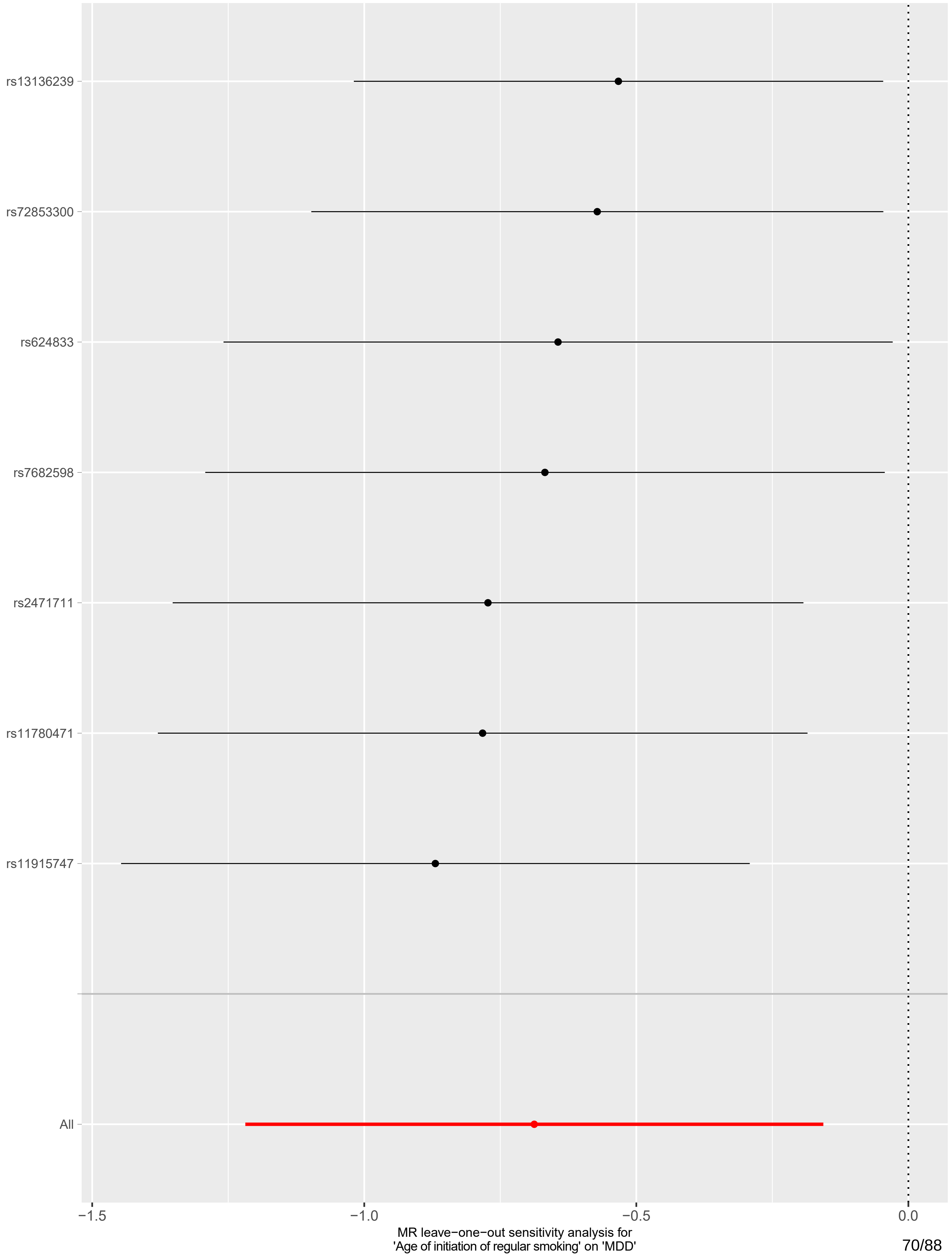

rs13136239
rs72853300
rs624833
rs7682598
rs2471711
rs11780471
rs11915747
All
−1.5
−1.0
−0.5
0.0
MR leave−one−out sensitivity analysis for
'Age of initiation of regular smoking' on 'MDD'

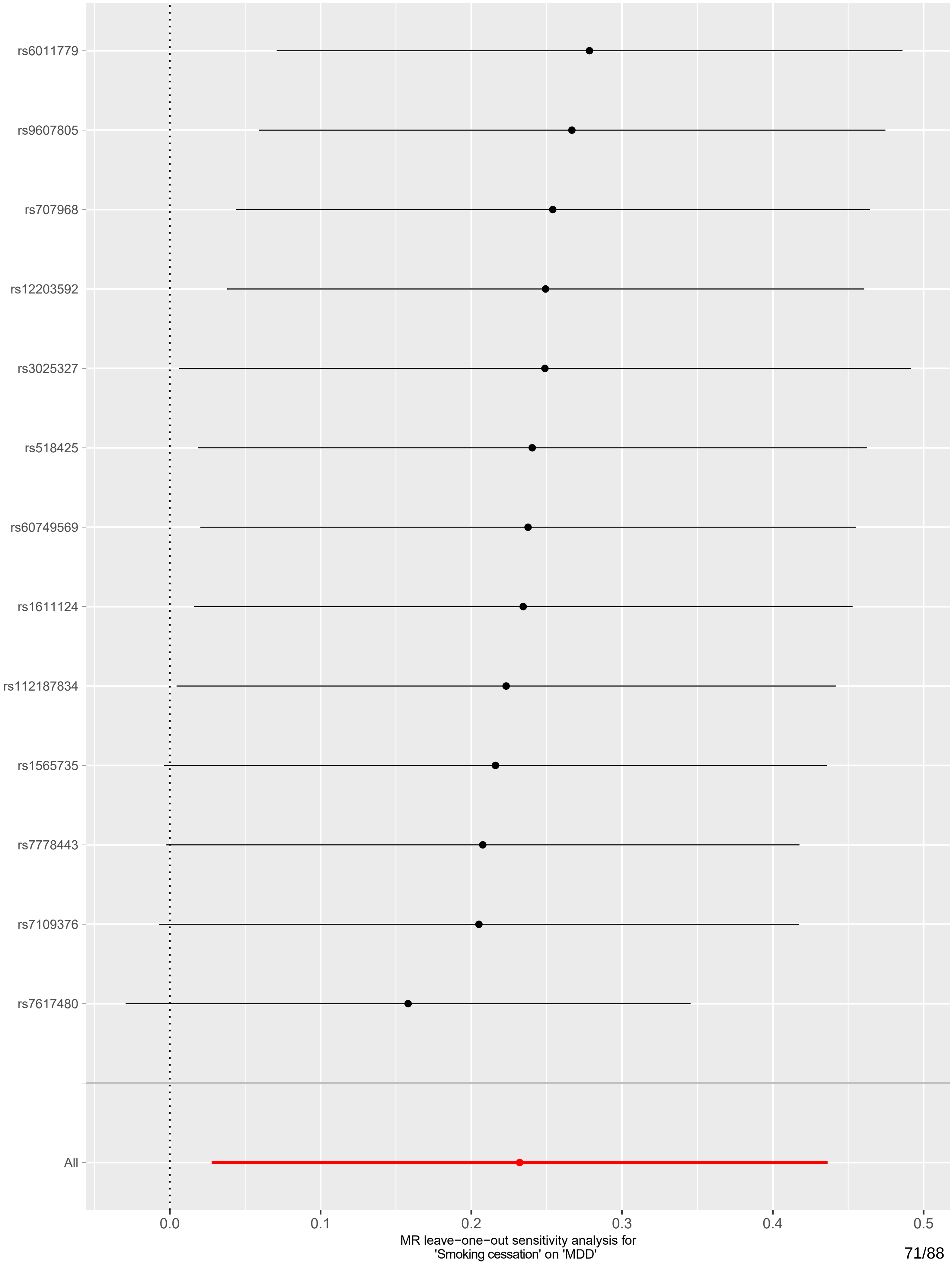

rs6011779
rs9607805
rs707968
rs12203592
rs3025327
rs518425
rs60749569
rs1611124
rs112187834
rs1565735
rs7778443
rs7109376
rs7617480
All
0.0
0.1
0.2
0.3
0.4
0.5
MR leave−one−out sensitivity analysis for
'Smoking cessation' on 'MDD'

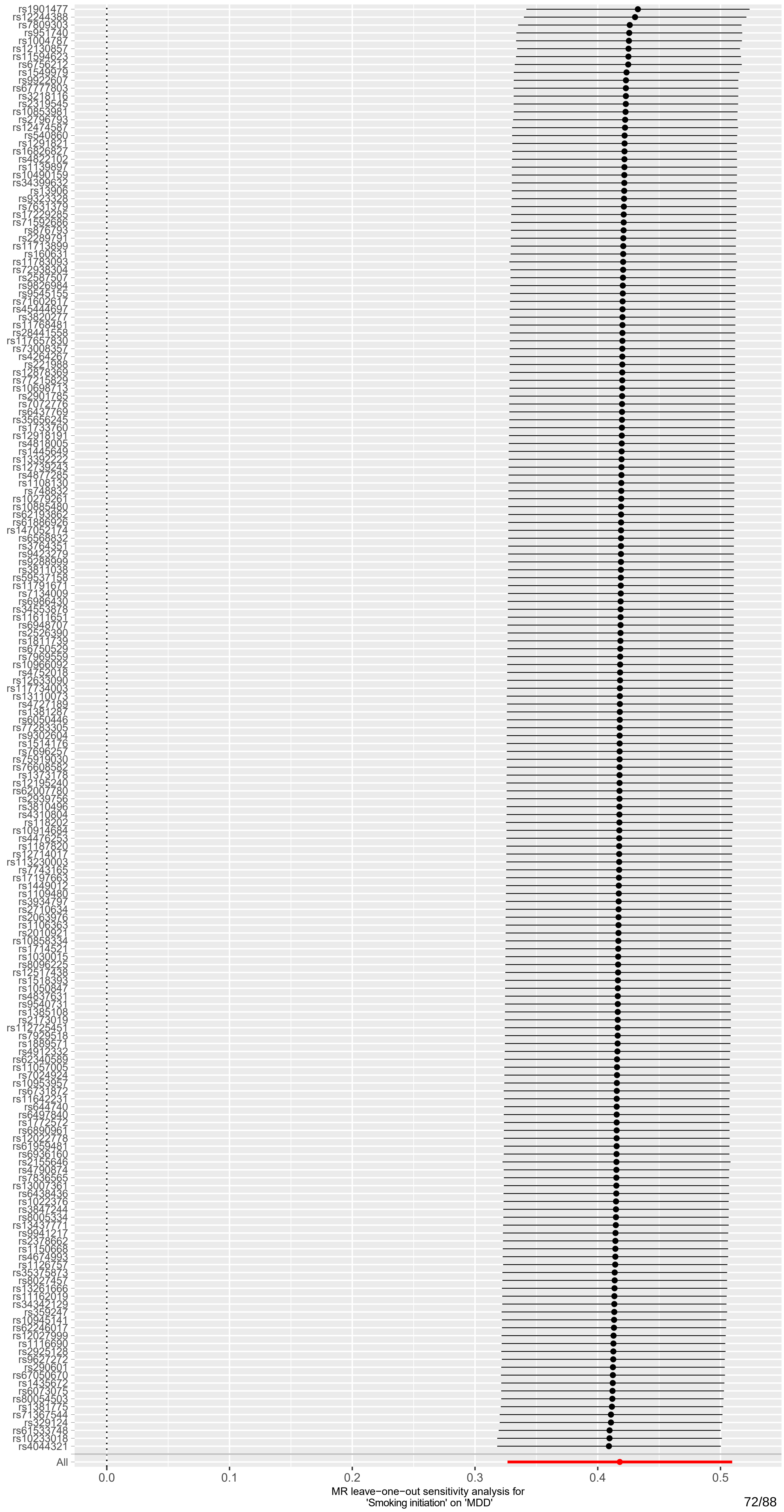
rs1901477
rs12244388
rs7809303
rs951740
rs1004787
rs12130857
rs11594623
rs6756212
rs1549979
rs9922607
rs67777803
rs3218116
rs2319545
rs10853981
rs2796793
rs12474587
rs540860
rs1291821
rs16826827
rs4822102
rs1139897
rs10490159
rs34399632
rs13906
rs9323328
rs7631379
rs17229285
rs71592686
rs876793
rs2289791
rs11713899
rs160631
rs11783093
rs72938304
rs2587507
rs9826984
rs9545155
rs71602617
rs45444697
rs3820277
rs11768481
rs28441558
rs117657830
rs73008357
rs4264267
rs221988
rs12878369
rs77215829
rs10698713
rs2901785
rs7072776
rs6437769
rs35656245
rs1733760
rs12918191
rs4818005
rs1445649
rs13392222
rs12739243
rs4877285
rs1108130
rs748832
rs10279261
rs10885480
rs62193862
rs61886926
rs147052174
rs6568832
rs3764351
rs9423279
rs9288999
rs3811038
rs59537158
rs11791671
rs7134009
rs6986430
rs34553878
rs11611651
rs6948707
rs2526390
rs1811739
rs6750529
rs7969559
rs10966092
rs4752018
rs12633090
rs117734003
rs13110073
rs4727189
rs1381287
rs6050446
rs77283305
rs9302604
rs1514176
rs7696257
rs75919030
rs76608582
rs1373178
rs12195240
rs62007780
rs2939756
rs3810496
rs4310804
rs118202
rs10914684
rs4476253
rs1187820
rs12714017
rs113230003
rs7743165
rs17197663
rs1449012
rs1109480
rs3934797
rs2710634
rs2063976
rs1106363
rs2010921
rs10858334
rs1714521
rs1030015
rs8096225
rs12517438
rs1518393
rs1050847
rs4837631
rs9540731
rs1385108
rs2173019
rs112725451
rs7929518
rs1889571
rs4912332
rs62340589
rs11057005
rs7024924
rs10953957
rs6731872
rs11642231
rs644740
rs6497840
rs1772572
rs6890961
rs12022778
rs61959481
rs6936160
rs2155646
rs4790874
rs7836565
rs13007361
rs6438436
rs1022376
rs3847244
rs8005334
rs13437771
rs9941217
rs2378662
rs1150668
rs4674993
rs1126757
rs35375873
rs8027457
rs13261666
rs11162019
rs34342129
rs359247
rs10945141
rs62246017
rs12027999
rs1116690
rs2925128
rs9627272
rs290601
rs67050670
rs1435672
rs6073075
rs80054503
rs1381775
rs71367544
rs329124
rs61533748
rs10233018
rs4044321
All
0.0
0.1
0.2
0.3
0.4
0.5
MR leave−one−out sensitivity analysis for
'Smoking initiation' on 'MDD'

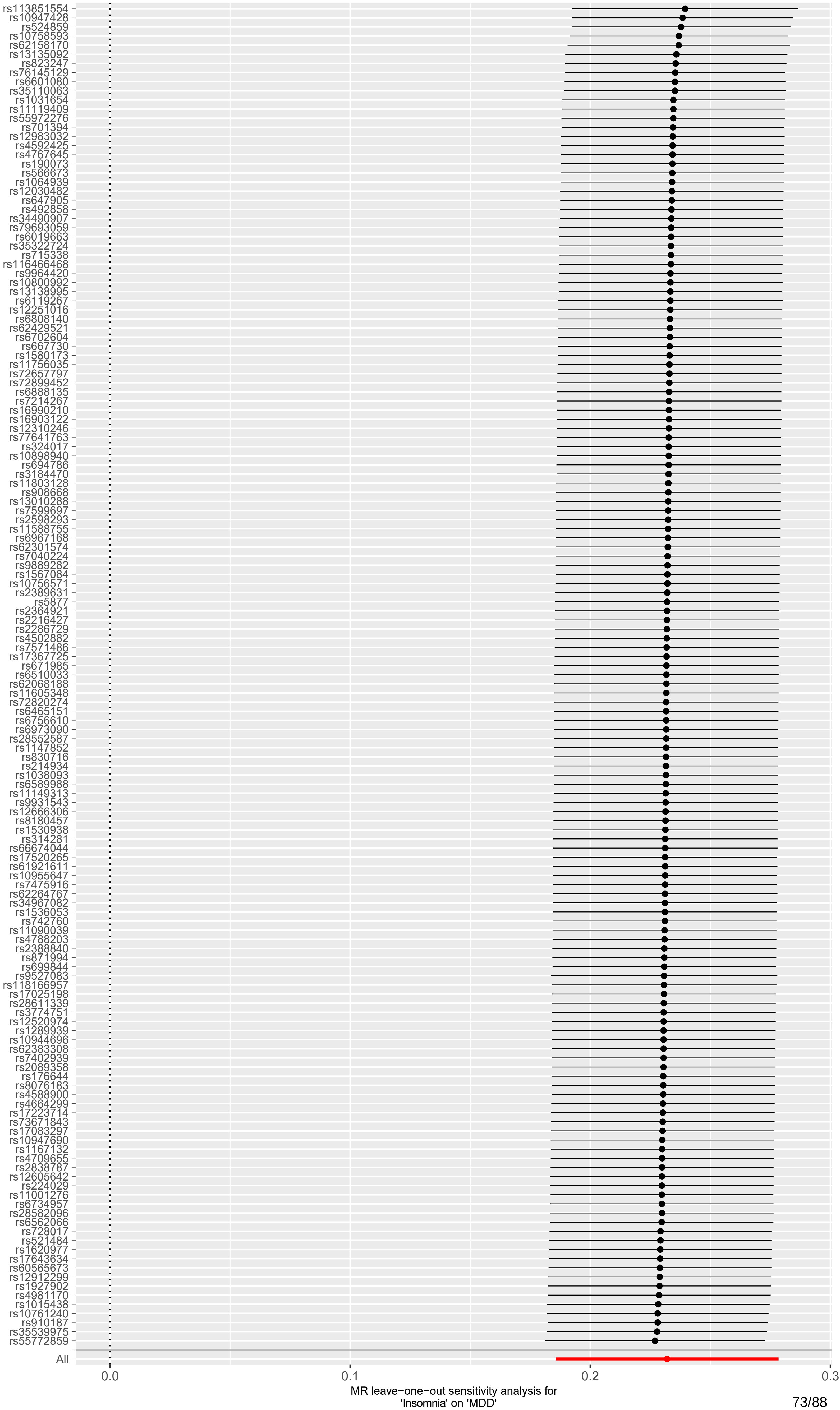
rs113851554
rs10947428
rs524859
rs10758593
rs62158170
rs13135092
rs823247
rs76145129
rs6601080
rs35110063
rs1031654
rs11119409
rs55972276
rs701394
rs12983032
rs4592425
rs4767645
rs190073
rs566673
rs1064939
rs12030482
rs647905
rs492858
rs34490907
rs79693059
rs6019663
rs35322724
rs715338
rs116466468
rs9964420
rs10800992
rs13138995
rs6119267
rs12251016
rs6808140
rs62429521
rs6702604
rs667730
rs1580173
rs11756035
rs72657797
rs72899452
rs6888135
rs7214267
rs16990210
rs16903122
rs12310246
rs77641763
rs324017
rs10898940
rs694786
rs3184470
rs11803128
rs908668
rs13010288
rs7599697
rs2598293
rs11588755
rs6967168
rs62301574
rs7040224
rs9889282
rs1567084
rs10756571
rs2389631
rs5877
rs2364921
rs2216427
rs2286729
rs4502882
rs7571486
rs17367725
rs671985
rs6510033
rs62068188
rs11605348
rs72820274
rs6465151
rs6756610
rs6973090
rs28552587
rs1147852
rs830716
rs214934
rs1038093
rs6589988
rs11149313
rs9931543
rs12666306
rs8180457
rs1530938
rs314281
rs66674044
rs17520265
rs61921611
rs10955647
rs7475916
rs62264767
rs34967082
rs1536053
rs742760
rs11090039
rs4788203
rs2388840
rs871994
rs699844
rs9527083
rs118166957
rs17025198
rs28611339
rs3774751
rs12520974
rs1289939
rs10944696
rs62383308
rs7402939
rs2089358
rs176644
rs8076183
rs4588900
rs4664299
rs17223714
rs73671843
rs17083297
rs10947690
rs1167132
rs4709655
rs2838787
rs12605642
rs224029
rs11001276
rs6734957
rs28582096
rs6562066
rs728017
rs521484
rs1620977
rs17643634
rs60565673
rs12912299
rs1927902
rs4981170
rs1015438
rs10761240
rs910187
rs35539975
rs55772859
All
0.0
0.1
0.2
0.3
MR leave−one−out sensitivity analysis for
'Insomnia' on 'MDD'

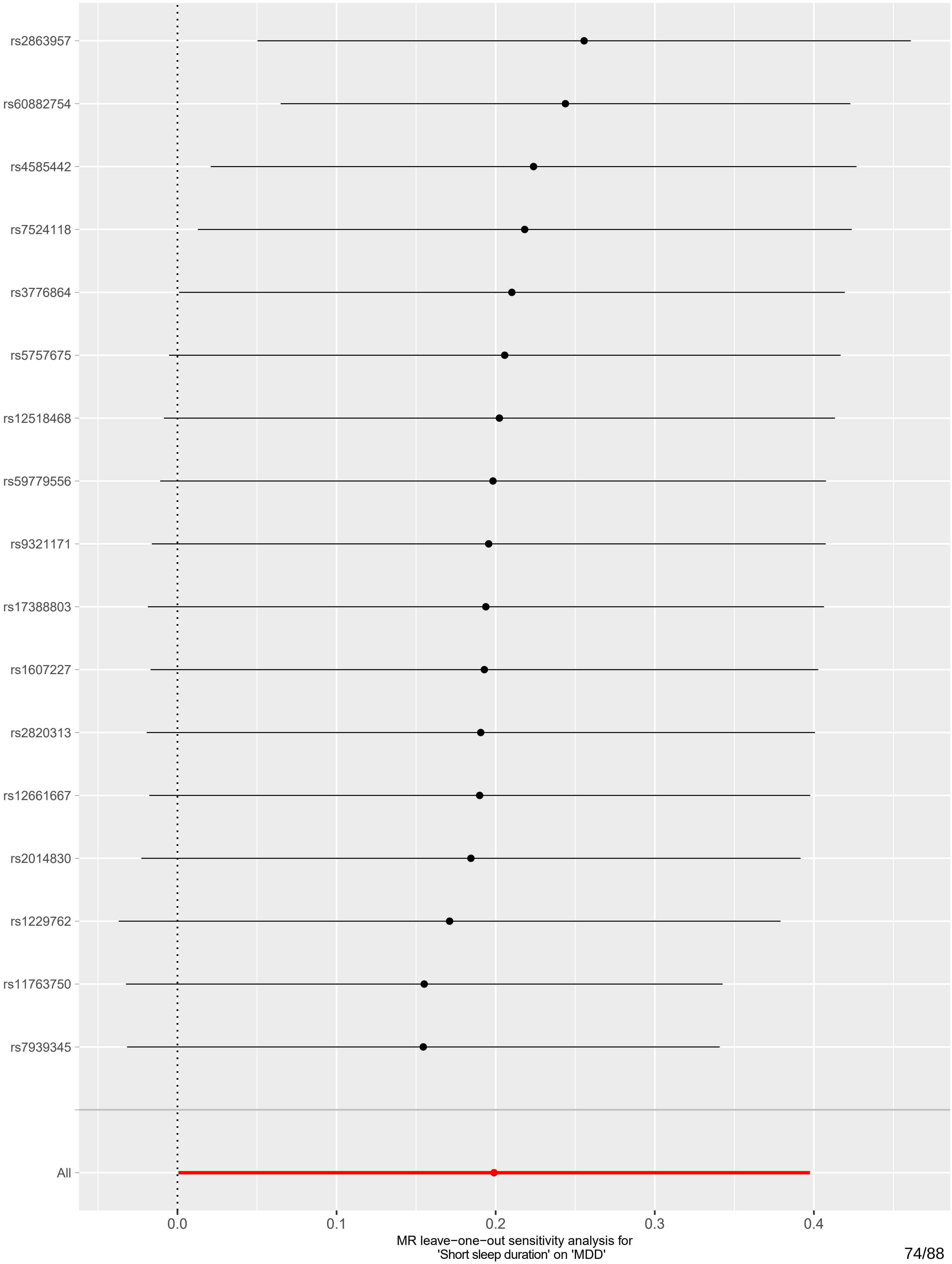

rs2863957
rs60882754
rs4585442
rs7524118
rs3776864
rs5757675
rs12518468
rs59779556
rs9321171
rs17388803
rs1607227
rs2820313
rs12661667
rs2014830
rs1229762
rs11763750
rs7939345
All
0.0
0.1
0.2
0.3
0.4
MR leave−one−out sensitivity analysis for
'Short sleep duration' on 'MDD'

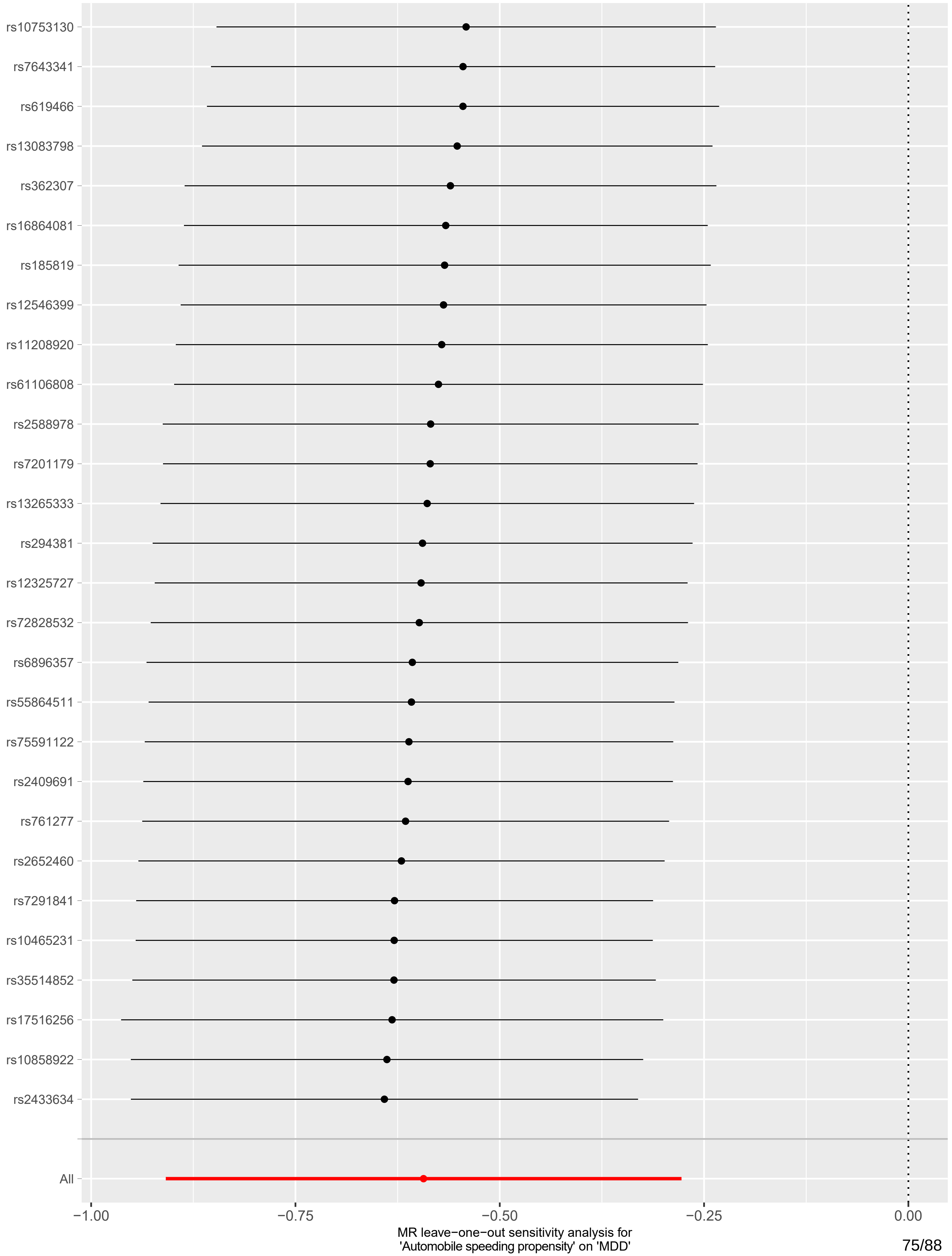

rs10753130
rs7643341
rs619466
rs13083798
rs362307
rs16864081
rs185819
rs12546399
rs11208920
rs61106808
rs2588978
rs7201179
rs13265333
rs294381
rs12325727
rs72828532
rs6896357
rs55864511
rs75591122
rs2409691
rs761277
rs2652460
rs7291841
rs10465231
rs35514852
rs17516256
rs10858922
rs2433634
All
−1.00
−0.75
−0.50
−0.25
0.00
MR leave−one−out sensitivity analysis for
'Automobile speeding propensity' on 'MDD'

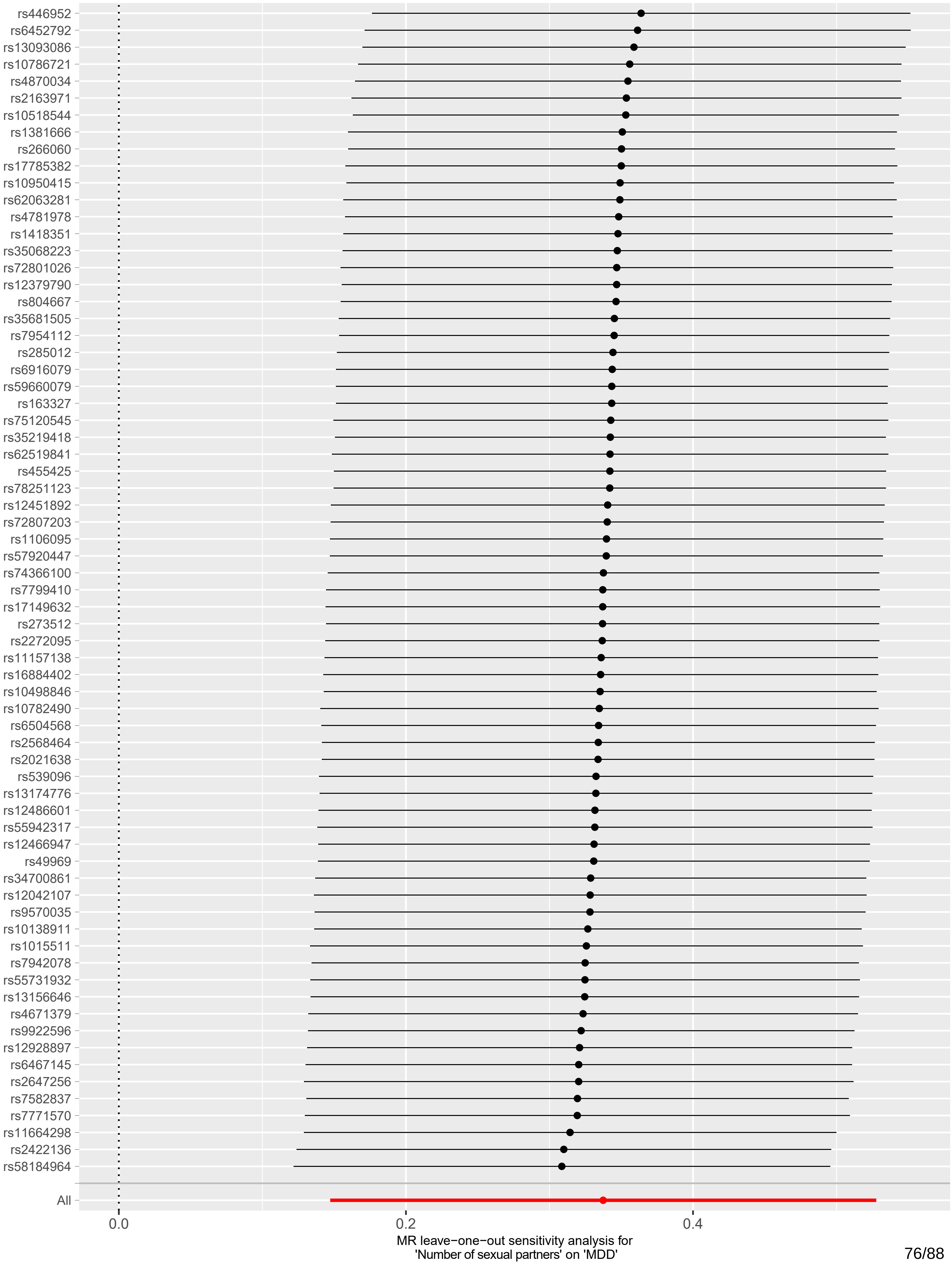

rs446952
rs6452792
rs13093086
rs10786721
rs4870034
rs2163971
rs10518544
rs1381666
rs266060
rs17785382
rs10950415
rs62063281
rs4781978
rs1418351
rs35068223
rs72801026
rs12379790
rs804667
rs35681505
rs7954112
rs285012
rs6916079
rs59660079
rs163327
rs75120545
rs35219418
rs62519841
rs455425
rs78251123
rs12451892
rs72807203
rs1106095
rs57920447
rs74366100
rs7799410
rs17149632
rs273512
rs2272095
rs11157138
rs16884402
rs10498846
rs10782490
rs6504568
rs2568464
rs2021638
rs539096
rs13174776
rs12486601
rs55942317
rs12466947
rs49969
rs34700861
rs12042107
rs9570035
rs10138911
rs1015511
rs7942078
rs55731932
rs13156646
rs4671379
rs9922596
rs12928897
rs6467145
rs2647256
rs7582837
rs7771570
rs11664298
rs2422136
rs58184964
All
0.0
0.2
0.4
MR leave−one−out sensitivity analysis for
'Number of sexual partners' on 'MDD'

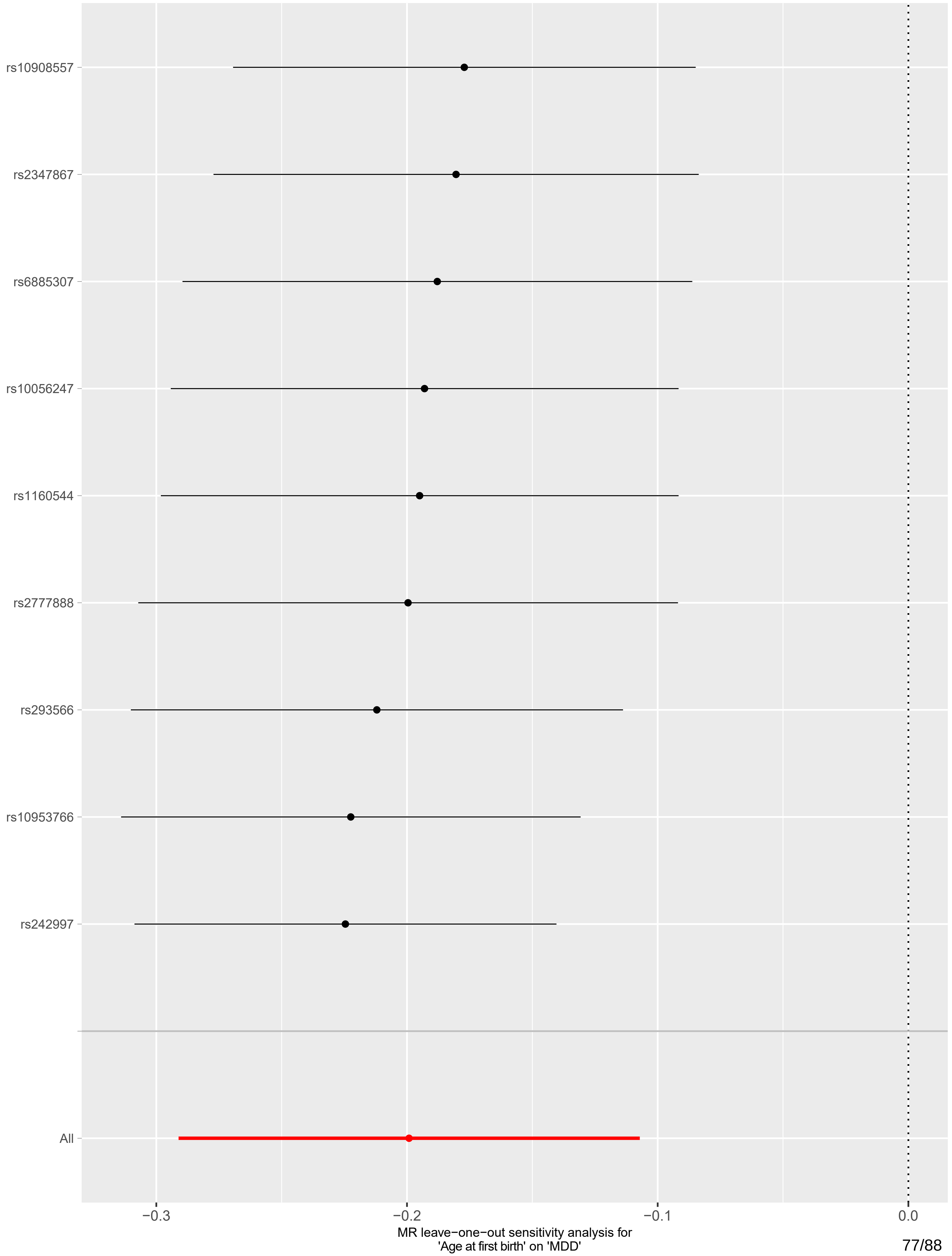
rs10908557
rs2347867
rs6885307
rs10056247
rs1160544
rs2777888
rs293566
rs10953766
rs242997
All
−0.3
−0.2
−0.1
0.0
MR leave−one−out sensitivity analysis for
'Age at first birth' on 'MDD'

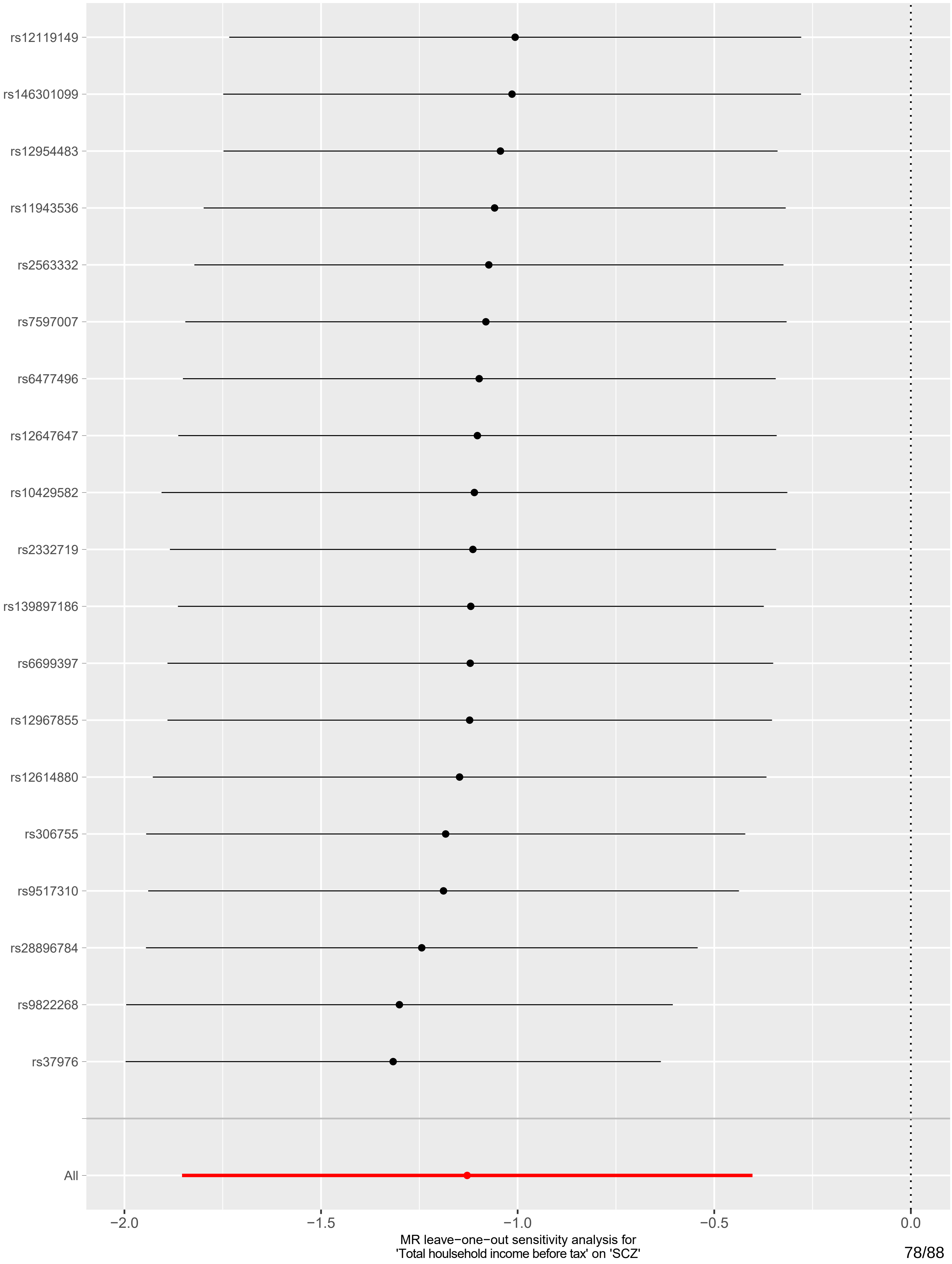
rs12119149
rs146301099
rs12954483
rs11943536
rs2563332
rs7597007
rs6477496
rs12647647
rs10429582
rs2332719
rs139897186
rs6699397
rs12967855
rs12614880
rs306755
rs9517310
rs28896784
rs9822268
rs37976
All
−2.0
−1.5
−1.0
−0.5
0.0
MR leave−one−out sensitivity analysis for
'Total houlsehold income before tax' on 'SCZ'

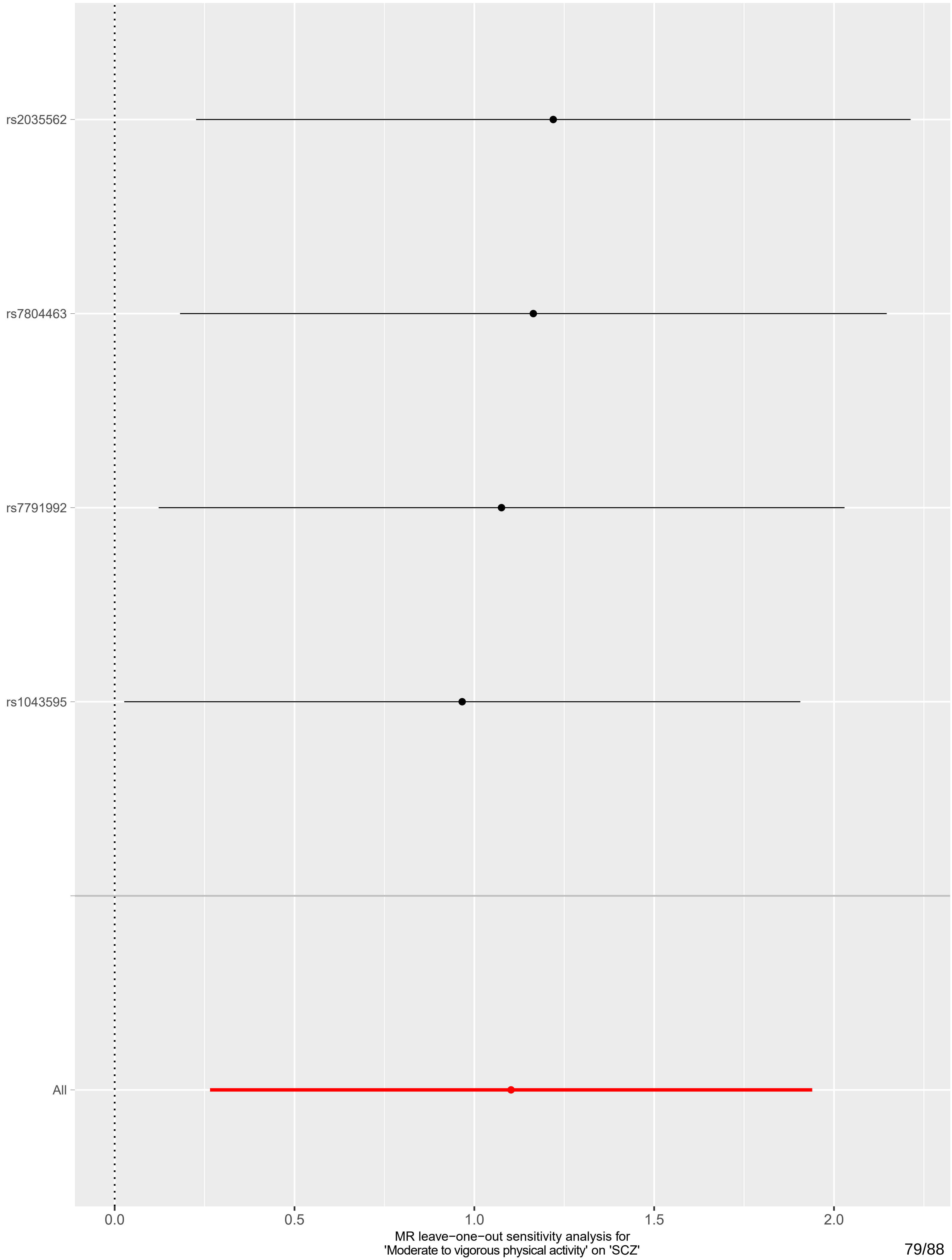
rs2035562
rs7804463
rs7791992
rs1043595
All
0.0
0.5
1.0
1.5
2.0
MR leave−one−out sensitivity analysis for
'Moderate to vigorous physical activity' on 'SCZ'

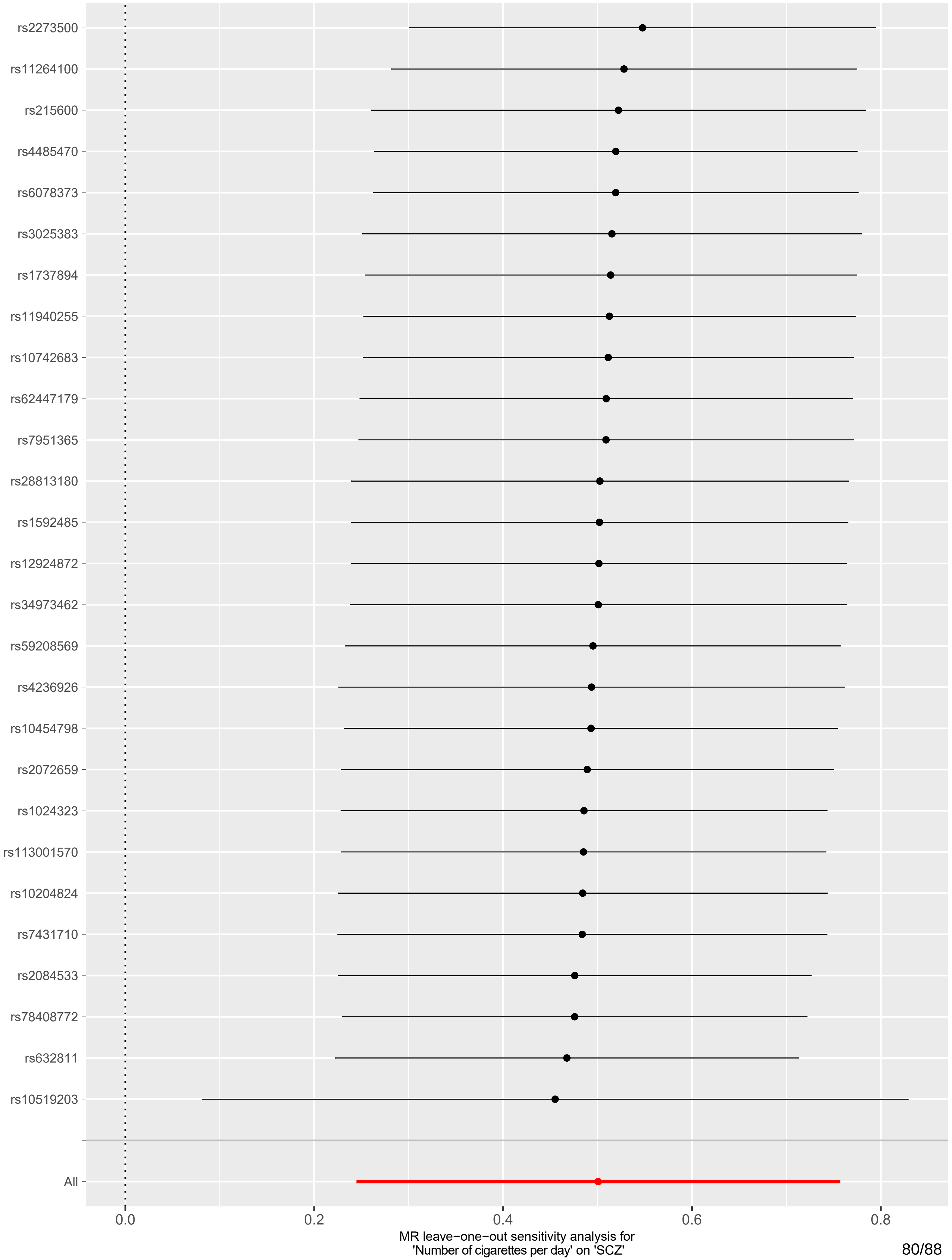
rs2273500
rs11264100
rs215600
rs4485470
rs6078373
rs3025383
rs1737894
rs11940255
rs10742683
rs62447179
rs7951365
rs28813180
rs1592485
rs12924872
rs34973462
rs59208569
rs4236926
rs10454798
rs2072659
rs1024323
rs113001570
rs10204824
rs7431710
rs2084533
rs78408772
rs632811
rs10519203
All
0.0
0.2
0.4
0.6
0.8
MR leave−one−out sensitivity analysis for
'Number of cigarettes per day' on 'SCZ'

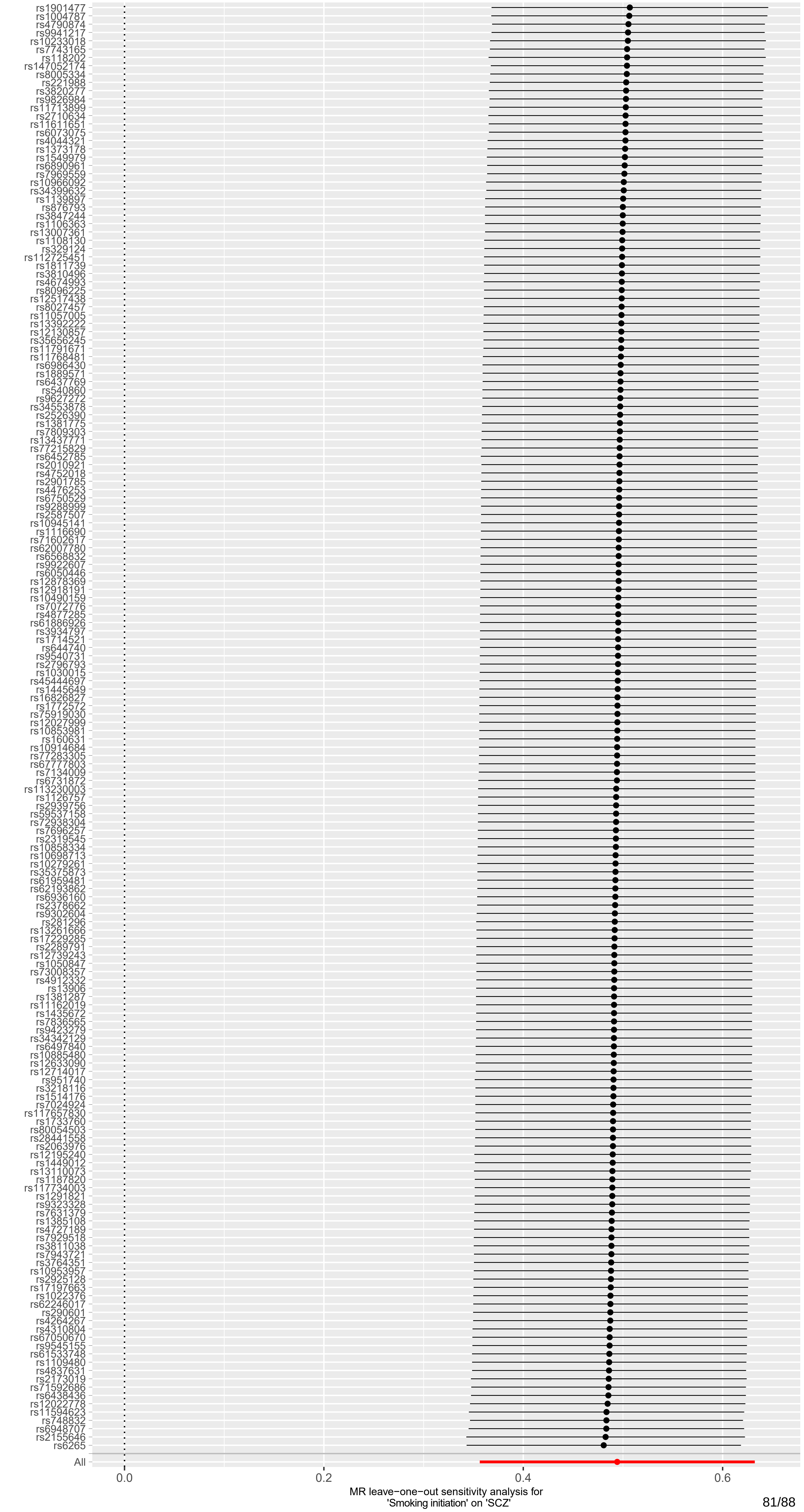
rs1901477
rs1004787
rs4790874
rs9941217
rs10233018
rs7743165
rs118202
rs147052174
rs8005334
rs221988
rs3820277
rs9826984
rs11713899
rs2710634
rs11611651
rs6073075
rs4044321
rs1373178
rs1549979
rs6890961
rs7969559
rs10966092
rs34399632
rs1139897
rs876793
rs3847244
rs1106363
rs13007361
rs1108130
rs329124
rs112725451
rs1811739
rs3810496
rs4674993
rs8096225
rs12517438
rs8027457
rs11057005
rs13392222
rs12130857
rs35656245
rs11791671
rs11768481
rs6986430
rs1889571
rs6437769
rs540860
rs9627272
rs34553878
rs2526390
rs1381775
rs7809303
rs13437771
rs77215829
rs6452785
rs2010921
rs4752018
rs2901785
rs4476253
rs6750529
rs9288999
rs2587507
rs10945141
rs1116690
rs71602617
rs62007780
rs6568832
rs9922607
rs6050446
rs12878369
rs12918191
rs10490159
rs7072776
rs4877285
rs61886926
rs3934797
rs1714521
rs644740
rs9540731
rs2796793
rs1030015
rs45444697
rs1445649
rs16826827
rs1772572
rs75919030
rs12027999
rs10853981
rs160631
rs10914684
rs77283305
rs67777803
rs7134009
rs6731872
rs113230003
rs1126757
rs2939756
rs59537158
rs72938304
rs7696257
rs2319545
rs10858334
rs10698713
rs10279261
rs35375873
rs61959481
rs62193862
rs6936160
rs2378662
rs9302604
rs281296
rs13261666
rs17229285
rs2289791
rs12739243
rs1050847
rs73008357
rs4912332
rs13906
rs1381287
rs11162019
rs1435672
rs7836565
rs9423279
rs34342129
rs6497840
rs10885480
rs12633090
rs12714017
rs951740
rs3218116
rs1514176
rs7024924
rs117657830
rs1733760
rs80054503
rs28441558
rs2063976
rs12195240
rs1449012
rs13110073
rs1187820
rs117734003
rs1291821
rs9323328
rs7631379
rs1385108
rs4727189
rs7929518
rs3811038
rs7943721
rs3764351
rs10953957
rs2925128
rs17197663
rs1022376
rs62246017
rs290601
rs4264267
rs4310804
rs67050670
rs9545155
rs61533748
rs1109480
rs4837631
rs2173019
rs71592686
rs6438436
rs12022778
rs11594623
rs748832
rs6948707
rs2155646
rs6265
All
0.0
0.2
0.4
0.6
MR leave−one−out sensitivity analysis for
'Smoking initiation' on 'SCZ'

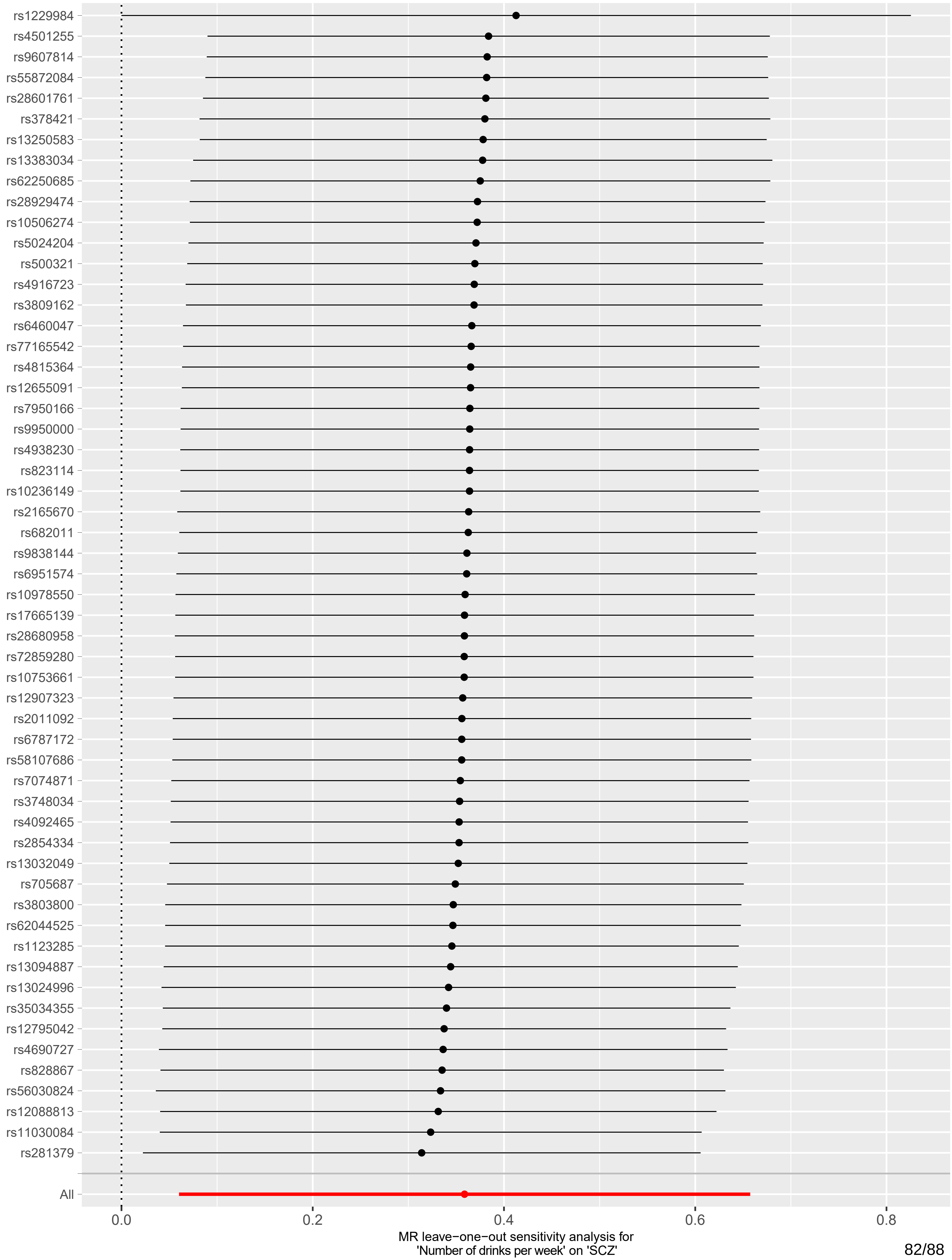
rs1229984
rs4501255
rs9607814
rs55872084
rs28601761
rs378421
rs13250583
rs13383034
rs62250685
rs28929474
rs10506274
rs5024204
rs500321
rs4916723
rs3809162
rs6460047
rs77165542
rs4815364
rs12655091
rs7950166
rs9950000
rs4938230
rs823114
rs10236149
rs2165670
rs682011
rs9838144
rs6951574
rs10978550
rs17665139
rs28680958
rs72859280
rs10753661
rs12907323
rs2011092
rs6787172
rs58107686
rs7074871
rs3748034
rs4092465
rs2854334
rs13032049
rs705687
rs3803800
rs62044525
rs1123285
rs13094887
rs13024996
rs35034355
rs12795042
rs4690727
rs828867
rs56030824
rs12088813
rs11030084
rs281379
All
0.0
0.2
0.4
0.6
0.8
MR leave−one−out sensitivity analysis for
'Number of drinks per week' on 'SCZ'

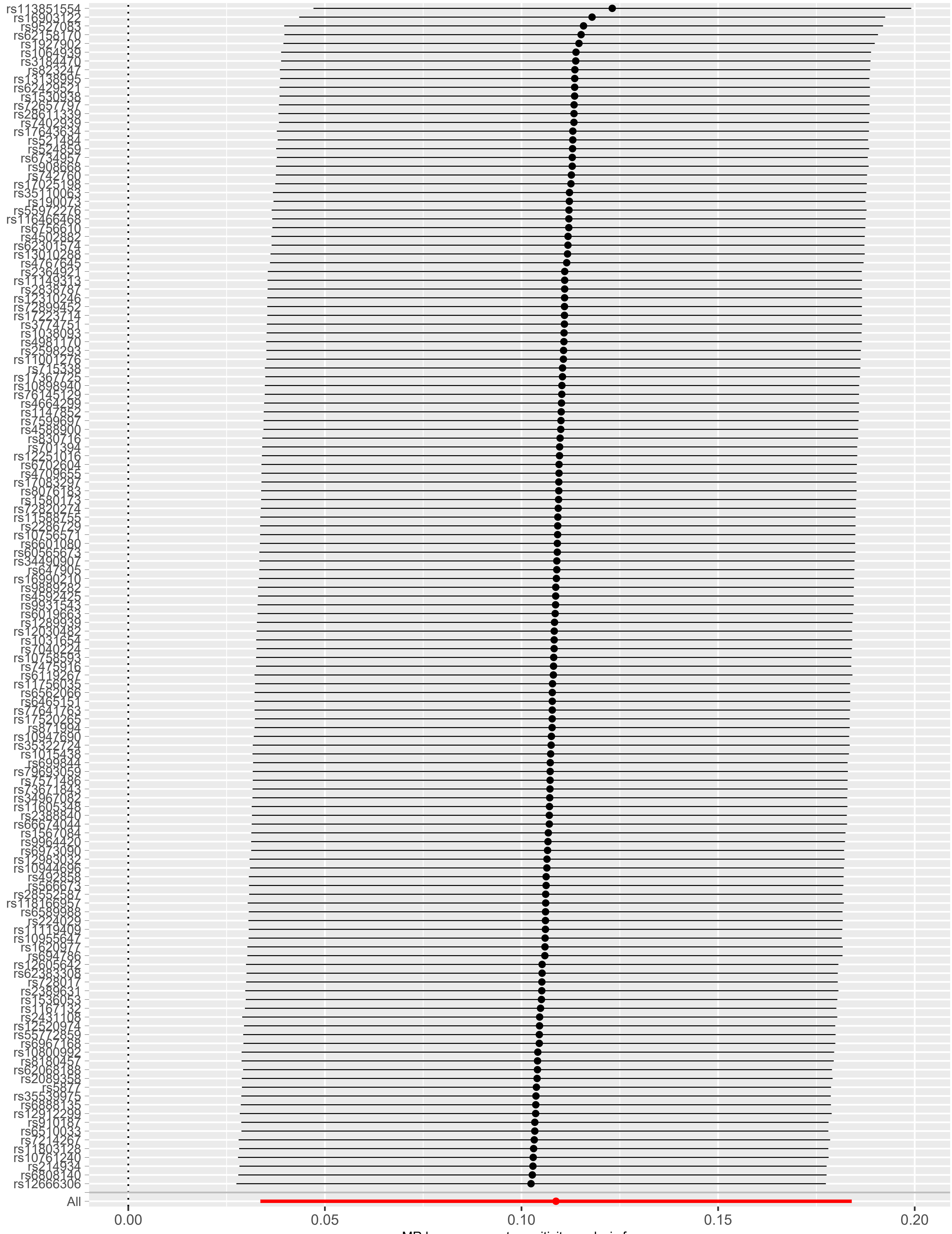


MR leave−one−out sensitivity analysis for
'Insomnia' on 'SCZ'

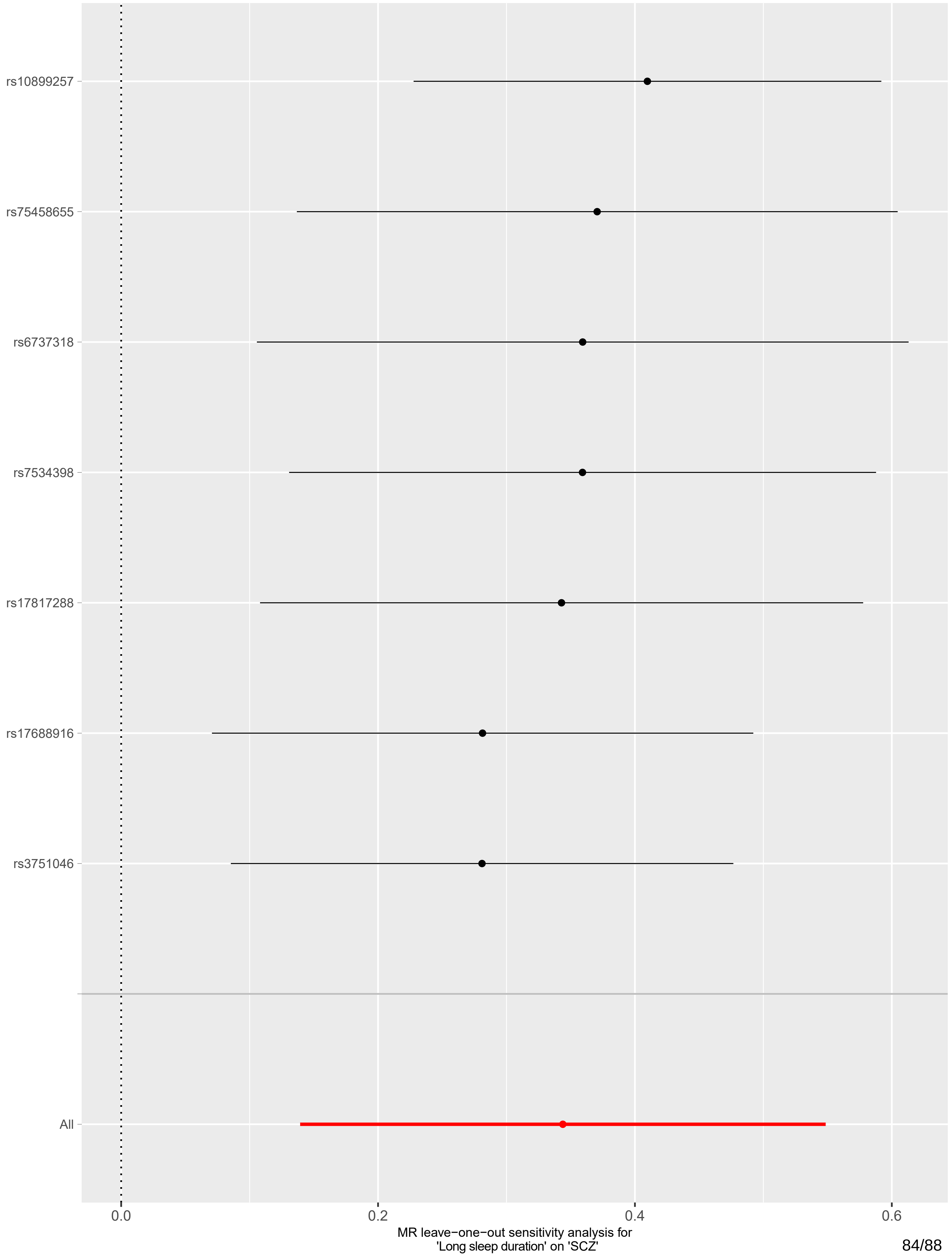

rs10899257
rs75458655
rs6737318
rs7534398
rs17817288
rs17688916
rs3751046
All
0.0
0.2
0.4
0.6
MR leave−one−out sensitivity analysis for
'Long sleep duration' on 'SCZ'

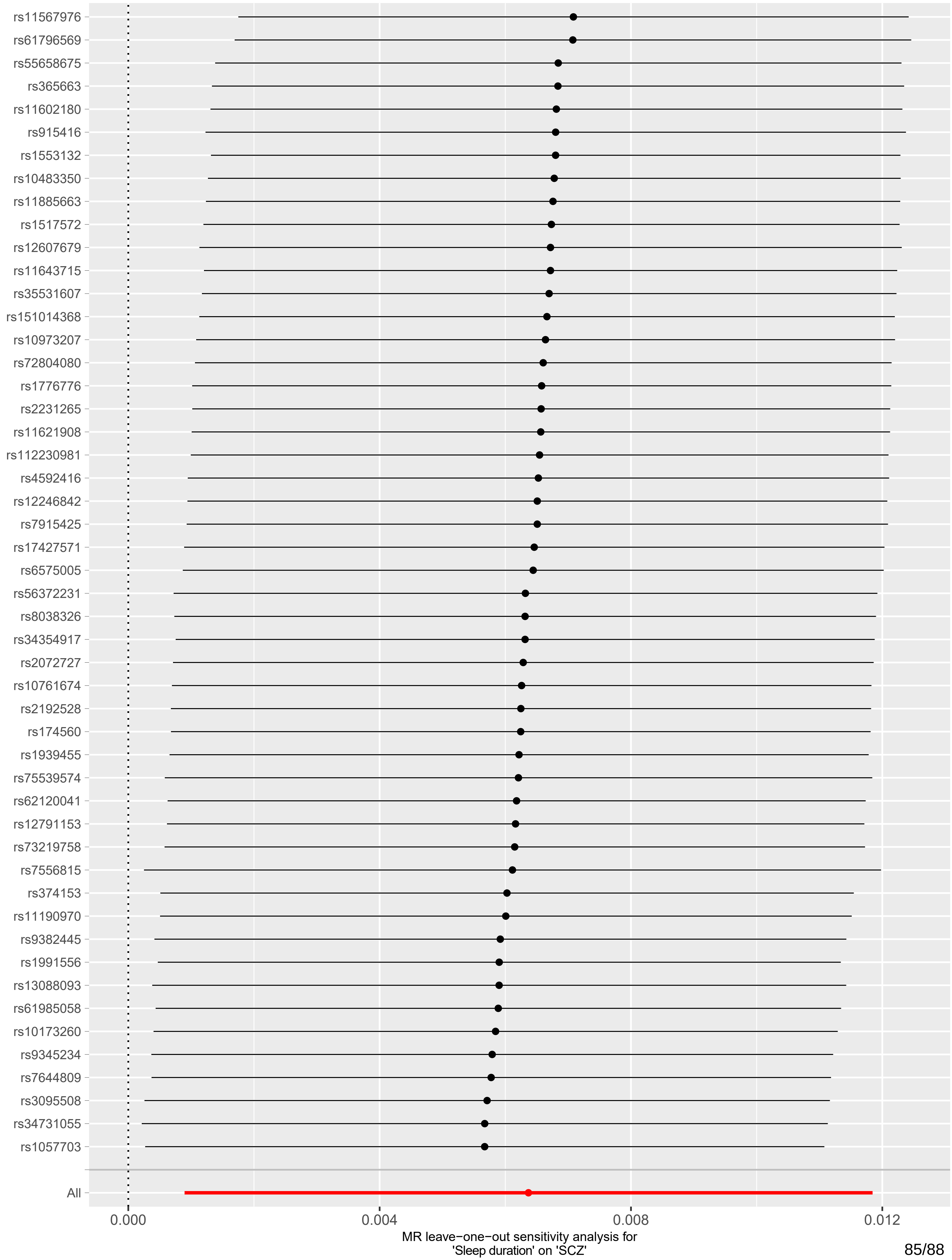
rs11567976
rs61796569
rs55658675
rs365663
rs11602180
rs915416
rs1553132
rs10483350
rs11885663
rs1517572
rs12607679
rs11643715
rs35531607
rs151014368
rs10973207
rs72804080
rs1776776
rs2231265
rs11621908
rs112230981
rs4592416
rs12246842
rs7915425
rs17427571
rs6575005
rs56372231
rs8038326
rs34354917
rs2072727
rs10761674
rs2192528
rs174560
rs1939455
rs75539574
rs62120041
rs12791153
rs73219758
rs7556815
rs374153
rs11190970
rs9382445
rs1991556
rs13088093
rs61985058
rs10173260
rs9345234
rs7644809
rs3095508
rs34731055
rs1057703
All
0.000
0.004
0.008
0.012
MR leave−one−out sensitivity analysis for
'Sleep duration' on 'SCZ'

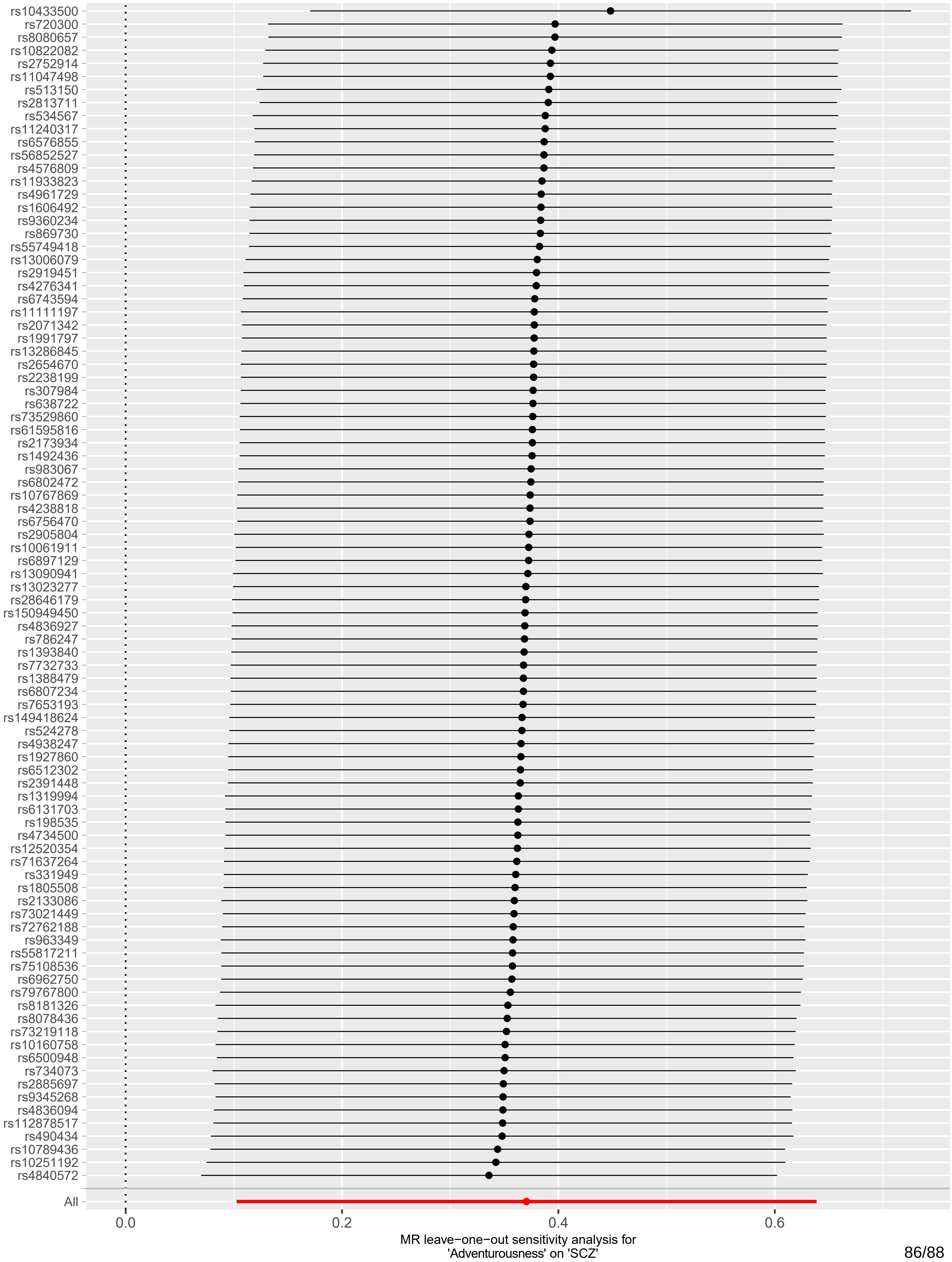
rs10433500
rs720300
rs8080657
rs10822082
rs2752914
rs11047498
rs513150
rs2813711
rs534567
rs11240317
rs6576855
rs56852527
rs4576809
rs11933823
rs4961729
rs1606492
rs9360234
rs869730
rs55749418
rs13006079
rs2919451
rs4276341
rs6743594
rs11111197
rs2071342
rs1991797
rs13286845
rs2654670
rs2238199
rs307984
rs638722
rs73529860
rs61595816
rs2173934
rs1492436
rs983067
rs6802472
rs10767869
rs4238818
rs6756470
rs2905804
rs10061911
rs6897129
rs13090941
rs13023277
rs28646179
rs150949450
rs4836927
rs786247
rs1393840
rs7732733
rs1388479
rs6807234
rs7653193
rs149418624
rs524278
rs4938247
rs1927860
rs6512302
rs2391448
rs1319994
rs6131703
rs198535
rs4734500
rs12520354
rs71637264
rs331949
rs1805508
rs2133086
rs73021449
rs72762188
rs963349
rs55817211
rs75108536
rs6962750
rs79767800
rs8181326
rs8078436
rs73219118
rs10160758
rs6500948
rs734073
rs2885697
rs9345268
rs4836094
rs112878517
rs490434
rs10789436
rs10251192
rs4840572
All
0.0
0.2
0.4
0.6
MR leave−one−out sensitivity analysis for
'Adventurousness' on 'SCZ'

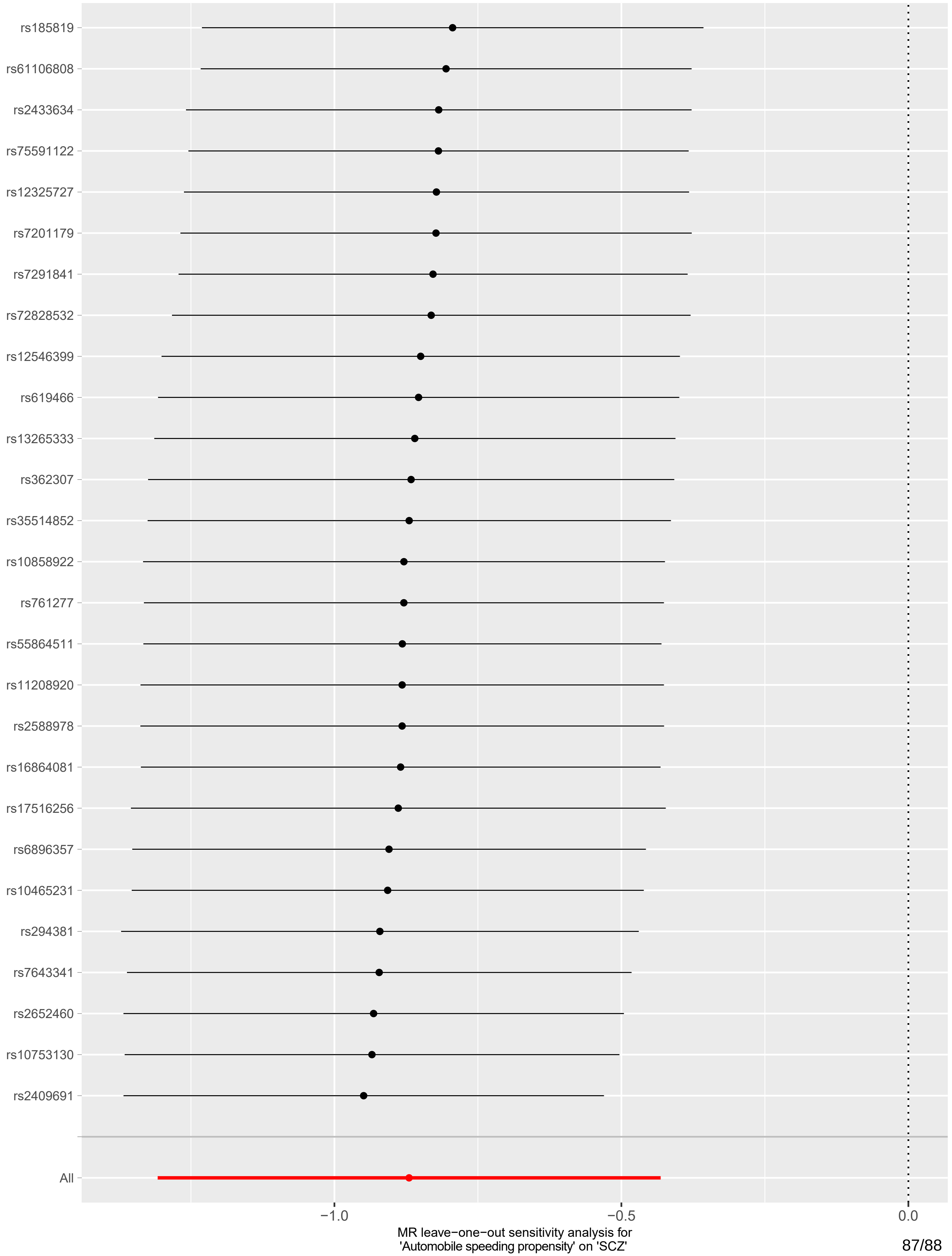
rs185819
rs61106808
rs2433634
rs75591122
rs12325727
rs7201179
rs7291841
rs72828532
rs12546399
rs619466
rs13265333
rs362307
rs35514852
rs10858922
rs761277
rs55864511
rs11208920
rs2588978
rs16864081
rs17516256
rs6896357
rs10465231
rs294381
rs7643341
rs2652460
rs10753130
rs2409691
All
−1.0
−0.5
0.0
MR leave−one−out sensitivity analysis for
'Automobile speeding propensity' on 'SCZ'

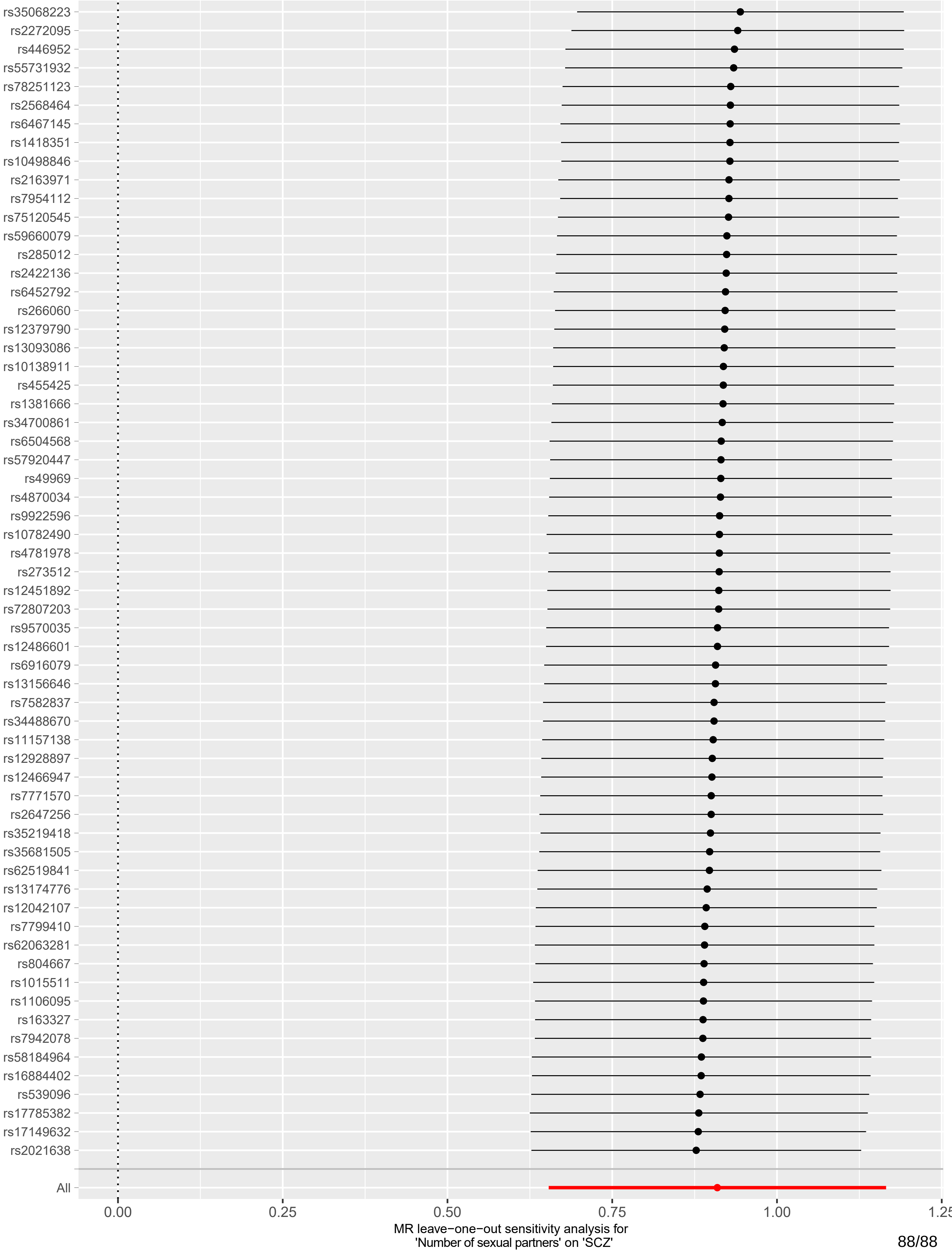
rs35068223
rs2272095
rs446952
rs55731932
rs78251123
rs2568464
rs6467145
rs1418351
rs10498846
rs2163971
rs7954112
rs75120545
rs59660079
rs285012
rs2422136
rs6452792
rs266060
rs12379790
rs13093086
rs10138911
rs455425
rs1381666
rs34700861
rs6504568
rs57920447
rs49969
rs4870034
rs9922596
rs10782490
rs4781978
rs273512
rs12451892
rs72807203
rs9570035
rs12486601
rs6916079
rs13156646
rs7582837
rs34488670
rs11157138
rs12928897
rs12466947
rs7771570
rs2647256
rs35219418
rs35681505
rs62519841
rs13174776
rs12042107
rs7799410
rs62063281
rs804667
rs1015511
rs1106095
rs163327
rs7942078
rs58184964
rs16884402
rs539096
rs17785382
rs17149632
rs2021638
All
0.00
0.25
0.50
0.75
1.00
1.25
MR leave−one−out sensitivity analysis for
'Number of sexual partners' on 'SCZ'
